# Macrogenetics Approach Reveals Spatial Trends and Drivers of Mitochondrial Genetic Diversity at Different Biological Organization Levels in Tropical Western Atlantic Decapods

**DOI:** 10.1002/ece3.71372

**Published:** 2025-05-14

**Authors:** Pedro A. Peres, Fernando L. Mantelatto

**Affiliations:** ^1^ Laboratory of Bioecology and Crustacean Systematics (LBSC), Department of Biology, Faculty of Philosophy, Sciences and Letters at Ribeirão Preto (FFCLRP) University of São Paulo (USP) São Paulo Brazil; ^2^ Institute of Environment, Department of Biology Florida International University Florida USA

**Keywords:** COI, Decapoda, invertebrate, macrogenetics, marine environment, molecular evolution

## Abstract

Recent studies explored whether the latitudinal diversity gradient extends to a latitudinal genetic diversity gradient. There is a knowledge gap concerning the genetic diversity distribution across marine invertebrates, and whether the latitudinal genetic diversity gradient results from congruent intraspecific trends. Here, we tested the hypotheses of the existence of a latitudinal mitochondrial genetic diversity gradient in marine decapods (crabs, hermit crabs, shrimps, lobsters) driven by environmental variables and that this gradient is the result of the accumulation of similar trends at the intraspecific level. We analyzed populational‐level cytochrome oxidase subunit I sequence available for Tropical Western Atlantic species (1883 sequences, 23 species) to investigate the association between mitochondrial genetic diversity versus latitude, and genetic diversity versus six environmental variables (sea surface temperature, dissolved oxygen, chlorophyll‐a, salinity, current velocity, pH). Intraspecific mitochondrial genetic diversity versus latitude analyses were also performed. Our findings indicate higher mitochondrial genetic diversity in lower latitudes (latitudinal genetic diversity gradient) driven by productivity and oxygen levels (only for nucleotide diversity). However, this trend is not caused by the accumulation of intraspecific patterns, which can be variable and species‐specific. Our results indicate that different levels of biological organization can show discordant patterns and suggest caution when interpreting macroscale investigations.

## Graphical abstract

There is a knowledge gap concerning the genetic diversity distribution across marine invertebrates, and whether the latitudinal genetic diversity gradient results from congruent intraspecific trends. Our results indicate higher genetic diversity in lower latitudes is correlated to productivity and oxygen levels, but does not represent the accumulation of intraspecific patterns.

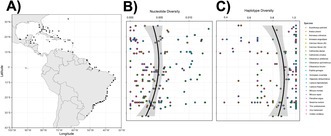

## 1 Introduction

Explaining the patterns and processes of biodiversity is one of the main challenges of ecology and evolution. Within this goal, questions addressing the genetic diversity spatial patterns are still open, and the emerging field of macrogenetics provides a framework to explore this topic (Leigh et al. 2021). In these types of studies, the mitochondrial DNA marker cytochrome c oxidase subunit I (COI) gene is often utilized because of its large availability in public repositories (Porter and Hajibabaei 2018). By generating genetic sequences for codistributed species and/or leveraging thousands of genetic sequences available in public repositories, the geographic distribution of genetic diversity of a taxonomic group across broad geographic scales can be correlated to environmental variables to test predictive hypotheses about the drivers of genetic diversity spatial variation (Miraldo et al. 2016; Manel et al. 2020; French et al. 2023).

Species diversity tends to be higher toward the tropics on a global scale, a well‐documented spatial pattern known as the latitudinal diversity gradient (Hawkins 2001; Hillebrand 2004; Kinlock et al. 2018). Studies addressing whether the latitudinal diversity gradient extends to genetic diversity support a broad‐scale latitudinal genetic diversity gradient (Miraldo et al. 2016; Gratton et al. 2017; Schluter and Pennell 2017). This pattern shows higher genetic diversity at lower latitudes, which decreases toward higher latitudes. However, the latitudinal diversity gradient (i.e., species diversity) is controversial when considering the marine environment. We find varying support in favor of the latitudinal diversity gradient depending on the taxa and region (Hillebrand 2004; Tittensor et al. 2010), but there are also cases showing an inverse latitudinal gradient, in which species diversity is lower at low latitudes (e.g., amphipods—Rivadeneira et al. 2011). Furthermore, there is an indication of a bimodal latitudinal species gradient in marine environments, which shows higher species diversity at intermediate latitudes around 30°–50° north and south (Chaudhary et al. 2016; Chaudhary et al. 2017). If genetic diversity follows the species diversity gradient, we may expect to find marine patterns different from those described for terrestrial environments, at least for a few taxa (Figuerola‐Ferrando et al. 2023).

Latitudinal genetic diversity gradients and drivers have been mainly investigated in terrestrial vertebrates and invertebrates such as mammals, birds, amphibians, and insects (e.g., Adams and Hadly 2013; Miraldo et al. 2016; Schär et al. 2017; Fonseca et al. 2023; French et al. 2023). There is a significant gap in knowledge concerning the patterns and processes of genetic diversity spatial distribution in marine species, and just a few studies exist (see Manel et al. 2020 and Clark and Pinsky 2024 for examples analyzing fish species), and tropical and subtropical marine invertebrate species are even less represented (but see Liggins et al. 2014; Figuerola‐Ferrando et al. 2023). Considering the marine environment, previous evidence indicates sea temperature is the main driver of this pattern in marine fish, being positively correlated to regions of high genetic diversity (Manel et al. 2020). The evolutionary speed hypothesis states that temperature influences substitution rates, leading to higher diversification in low latitudes, representing the mechanism behind this trend (Dowle et al. 2013; Mittelbach et al. 2007). But other environmental variables can also be important, like productivity and depth, which can be important in generating higher species and genetic diversity in sessile benthic taxa (Figuerola‐Ferrando et al. 2023) and investigations looking at a broad range of marine taxa (Chaudhary et al. 2016). Therefore, general mechanisms behind marine taxa trends are less clear and currently seem to be taxa/region/dataset specific, requiring further studies.

Many macrogenetics studies looking at latitudinal genetic diversity gradients use a total genetic diversity metric (“a sum or mean of genetic diversity across all species and their populations within a given area,” Lawrence and Fraser 2020), which does not capture intraspecific trends. Although the same forces may govern intra‐ and interspecific genetic diversity (Antonovics 2003; Vellend et al. 2014), another main open question is whether the latitudinal genetic diversity gradient results from the accumulation of intraspecific patterns or represents an emergent pattern resulting from the combination of species‐specific trends (French et al. 2023). At the intraspecific level, multiple hypotheses exist to explain genetic diversity spatial variation besides a latitudinal gradient. The central–marginal hypothesis (Eckert et al. 2008; Sagarin and Gaines 2002) states that genetic diversity would be spatially distributed according to the species' distributional range, being higher at the center of the species distribution because this region would also represent a peak in abundance. The central–marginal hypothesis and the latitudinal genetic diversity gradient can be combined (Guo 2012), resulting in higher genetic diversity at the central areas of the distribution than at the marginal populations, and populations on the lower latitudes side show higher genetic diversity than higher‐latitude populations. Adding uncertainty to the issue, marine species might not show any genetic diversity spatial distribution trend due to connectivity among populations in this complex system. The marine species hypothesis states that common features in marine species might hamper the emergence of genetic diversity spatial trends (Liggins et al. 2015). Features like large population sizes and high migration rates among populations caused by pelagic larvae dispersal would prevent specific population dynamics that could generate some of the spatial patterns described (Palumbi 1994). Therefore, the marine species hypothesis can function as a null hypothesis when investigating the spatial distribution of genetic diversity by representing a lack of any spatial trend. These potential explanations illustrate how convoluted the spatial distribution of genetic diversity can be and that many questions remain to be answered.

Although progress has been made in investigating genetic diversity spatial trends and drivers, previous studies have investigated total genetic diversity without including the intraspecific component (e.g., Manel et al. 2020), combining marine species from multiple regions (Figuerola‐Ferrando et al. 2023), or not looking at the marine environment (e.g., Fonseca et al. 2023). Additionally, studies tend to focus on global scales (e.g., Manel et al. 2020) or in temperate regions (Schär et al. 2017). Considering the gaps presented, a potential way to further our understanding of the connections between intraspecific and total genetic diversity spatial distribution in the marine environment is to focus on invertebrate species from the same taxa, from a specific region, with overlapping distribution. Such framework allows accessing intraspecific and total genetic diversity trends and drivers and eliminates bias regarding pooling phylogenetically distant species under distinct historical contexts and extremely different environmental variables. Decapods are one of the most abundant invertebrates in marine habitats (e.g., crabs, hermit crabs, shrimps, and lobsters), with around 17,200 extant species (Davis et al. 2022; De Grave et al. 2023). These species show a variable number of larval stages and dispersal potential (Anger 2001), thus representing a potential marine invertebrate model to address the questions in hand. Under this framework, the Tropical Western Atlantic is ideal for discussing latitudinal gradients due to its north–south arrangement and lack of extreme longitudinal variation along its coastal environments. The only assessment of the latitudinal diversity gradient (species diversity) for decapods indicates a bimodal distribution, with species diversity peaking not in the Equator but in the tropical north and south regions around 25° (Rivadeneira and Poore 2020). An investigation of some mangrove crab families using distribution points across the globe shows a latitudinal diversity gradient (i.e., peaking on the Equator; Sharifian et al. 2020). Investigations on crabs and hermit crabs indicate these groups show higher species diversity as latitude decreases along the temperate Western Atlantic and Southeastern Pacific regions (South America coast) (Macpherson 2002; Astorgaet al. 2003; Fernández et al. 2009; Levinton and Mackie 2013; Pappalardo and Fernández 2014). In many of the mentioned examples, the factors shaping species diversity spatial distribution are unclear. Currently, no studies have investigated genetic diversity spatial trends across decapod species under a macrogenetics framework. Whether decapod trends in species diversity are similar to genetic diversity trends remains unknown.

Here, we employed a macrogenetics approach to address (1) the existence of a latitudinal genetic diversity gradient in marine decapods and whether environmental factors are driving such a pattern. We hypothesize that (1a) total genetic diversity level follows a general latitudinal diversity gradient (peak in the Equator) and is mostly explained by sea temperature; or (1b) genetic diversity follows alternative distribution trends found in other marine species and decapods. We also investigated (2) if this gradient results from accumulating similar trends at the intraspecific level. In this case, we hypothesize (2a) intraspecific trends follow the total genetic diversity trend; or (2b) intraspecific trends can be variable.

## 2 Materials and Methods

### 2.1 Decapod Species Genetic Data

**FIGURE 1:**
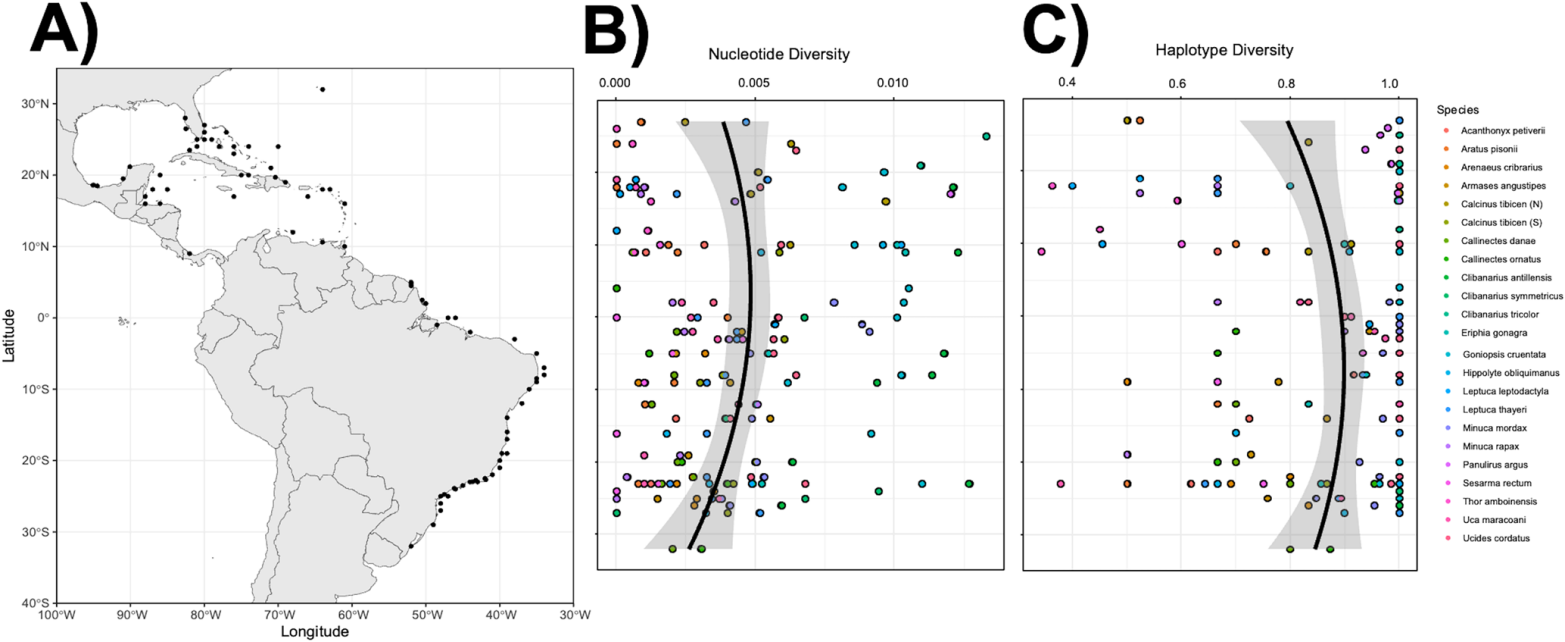
(A) Map showing the latitudinal range analyzed. Dots represent all sites from where specimens were collected. (B) The association between latitude and total nucleotide genetic diversity indicating higher diversity toward the equator. (C) The association between latitude and total haplotype genetic diversity indicating higher diversity toward the equator. For graphs (B, C), different colors represent different species, and a trend line with standard error is indicated. Graphs (B, C) are oriented following the latitudes indicated in Figure A.

Unlike groups commonly used in macrogenetics studies, decapod (and many invertebrates) sequences could not be retrieved from automated searches and required manual data mining due to some limitations. For instance, automated searches in Barcode of Life Data System (BOLD) did not retrieve sequences from a great part of the Southwestern Atlantic that we knew existed based on our knowledge of previous studies; many entries in National Center for Biotechnology Information (NCBI) are not georeferenced and could not be used in our study considering our explicit use of geographical information in our analyses (see below); many authors submit unique haplotypes, limiting the use of these sequences for populational‐level studies unless the original study and metadata are found. Therefore, we performed a manual exhaustive search on Google Scholar in April/2024 using the keywords combination “Western Atlantic”/“Tropical Western Atlantic” + “cytochrome oxidase”/”COI” + “decapod*” and examined all studies. We selected species that (1) had most of their natural distribution in the Tropical Western Atlantic (data from outside the tropics was included when it represented the edges of the species distribution; invasive species were not included); (2) had sampling locations available in the published paper so we could retrieve latitude information; (3) had at least three sequences available per latitudinal band (one degree) and data distributed across four or more different latitudinal bands; and (4) had individual haplotype data that allowed us the reconstruct haplotype frequencies per latitudinal band, or from which unique haplotypes were deposited and we got haplotype frequencies directly from the corresponding author upon contact. The reasoning behind these steps was to guarantee we analyzed tropical species with overlapping distribution, minimized the impact of sample size on the genetic diversity estimation (Goodall‐Copestake et al. 2012), and maximized the number of sampling locations following thresholds used in similar studies (French et al. 2023; Schär et al. 2017). In the case of studies concluding a species was represented by cryptic species or a complex, we evaluated each monophyletic group separately following the inclusion criteria mentioned above. For instance, *Callinectes ornatus* (Peres et al. 2022; Peres and Mantelatto 2020) and *Calcinus tibicen* (Mandai et al. 2018) were thought to be distributed along the Western Atlantic, but genetic analyses showed they are represented by two separated lineages. Lineages were evaluated separately leading to the inclusion of just one lineage of *C. ornatus* , and both lineages of *C. tibicen* (see Table 1 for their geographical distribution). This manual exhaustive search followed by filtering steps and inclusion criteria were preferred over pulling sequences directly from public databases for better control of the sequences included with the goal of ensuring the most representative and geographically distributed sample to test our hypotheses, using the most reliable dataset we could get. Our final dataset consisted of 23 species and 1883 COI sequences from a latitudinal range of 36° N to 32° S (Tables 1, S1 and Figure 1A), representing species from shallow and coastal water habitats (intertidal, infralittoral, or mangrove). All sequences were visually inspected in the alignment using Geneious Prime 2022.2.2 (https://www.geneious.com), and short or low‐quality sequences were not used. GenBank accession numbers and locations for each individual used can be found in Table S1.

**TABLE 1:** Species analyzed to investigate the association between mitochondrial genetic diversity and latitude, and mitochondrial genetic diversity and sea surface temperature.

Species		NLS	PLD	LR	*N*	Reference
Crabs						
*Acanthonyx petiverii*		3	~15	10 N–23S	41	Tamburus and Mantelatto (2016), present study
*Aratus pisonii*		5	~28	27 N–23S	61	Buranelli and Mantelatto 2019
*Arenaeus cribrarius*		9	~48	36 N–26S	44	Zupolini et al. (2017), present study
*Armases angustipes*		5	28	2S–25S	66	Marochi et al. 2017
*Callinectes danae*		9	NA	9 N–32S	54	Peres and Mantelatto 2020
*Callinectes ornatus*		9	NA	4 N–32S	44	Peres and Mantelatto (2020), Peres et al. (2022)
*Eriphia gonagra*		5	NA	10 N–23S	40	Present study
*Goniopsis cruentata*		7	NA	20 N–25S	62	Buranelli and Mantelatto 2019
*Leptuca leptodactyla*		6 to 7	~24	19 N–23S	86	Laurenzano et al. (2016)
*Leptuca thayeri*		6	30	27 N–27S	61	Buranelli and Mantelatto 2019
*Minuca mordax*		6 to 7	~25	2 N–29S	113	Martins et al. (2024)
*Minuca rapax*		6	~31	28 N–25S	57	Thurman et al. (2019)
*Sesarma rectum*		4	16	10 N–24S	42	Buranelli and Mantelatto 2017
*Uca maracoani*		6	NA	19 N–25S	103	Marochi et al. (2022)
*Ucides cordatus*		7	58	23 N–23S	52	Buranelli et al. (2019)
Hermit crabs						
*Calcinus tibicen* (N)		7 to 9	32 to 42	27 N–9 N	36	Mandai et al. (2018)
*Calcinus tibicen* (S)		7 to 9	32 to 42	3S–27S	28	Mandai et al. (2018)
*Clibanarius antillensis*		6 to 7	~43	18 N–26S	37	Nishikawa et al. (2021).
*Clibanarius symmetricus*		5 to 6	~26	0–27S	22	Negri et al. (2014)
*Clibanarius tricolor*				25 N–12 N	167	Stark et al. (2021)
Shrimps						
*Hippolyte obliquimanus*		NA	NA	10 N–27S	39	Terossi and Mantelatto (2012)
*Thor amboinensis*		9	~28	32 N–9 N	302	Titus et al. (2018).
Lobster						
*Panulirus argus*		10	140 to 198	26 N–17 N	326	Naro‐Maciel et al. (2011)

Abbreviations: LR, latitudinal range analyzed; N, number of sequences used; NLS, number of larval stages (zoea + megalopa); PLD, pelagic larval duration in days.

### 2.2 Genetic Diversity

For each sampling location, we considered only absolute latitude values and pooled locations within the same latitudinal band (one degree) for the subsequent analyses (i.e., if one sampling location was at 23°34’S and the other at 23°12’S, both were considered from the 23° latitudinal band), resulting in 43 unique latitudinal bands. We estimated nucleotide and haplotype diversity for each latitudinal band for each species using DnaSP (Rozas et al. 2017). These two metrics were selected because they represent different facets of genetic diversity. Nucleotide diversity represents the average number of nucleotide differences per site between two randomly selected sequences within a specific set of sequences (Nei 1987). Haplotype diversity represents the probability that two randomly selected haplotypes differ, not accounting for the magnitude of their differences (Nei 1987). A total of 191 genetic diversity estimates (nucleotide and haplotype diversity) were calculated across the 43 latitudinal bands ranging from 36° N to 32° S.

### 2.3 Environmental Data

We extracted six environmental variables from Bio‐ORACLE v2 (Assis et al. 2018): mean sea surface temperature (BO_sstmean), dissolved oxygen (BO_dissox), salinity (BO_salinity), current velocity at mean depth (BO2_curvelmean_bdmean), mean chlorophyll‐a (BO_chlomean), pH (BO22_ph) (Figure S1). Variables were selected due to what they represent to habitat suitability and their known role in driving species distribution, which could potentially influence genetic diversity as well (Lawrence and Fraser 2020; Melo‐Merino et al. 2020; Mittelbach et al. 2007). Specifically: temperature, oxygen, salinity, and pH represent physiological tolerances and metabolic rates; current velocity represents dispersal and habitat selection (e.g., settlement preference); chlorophyll‐a is a proxy for productivity. Data was collected using latitude/longitude associated with each individual. When different points from the same latitudinal band were used, we calculated the average of these values.

### 2.4 Latitude Versus Genetic Diversity

Only latitudes with data for more than one species were included in the total genetic diversity analyses to avoid biases. Before running the analyses, we used linear regressions to investigate the association between the number of individuals used to calculate each genetic diversity metric and latitudinal band. The normality, homoscedasticity, and independence of residuals were visually inspected on residual plots (Boldina and Beninger 2016). After excluding outliers (i.e., intraspecific latitudinal bands, *n* = 16 for nucleotide diversity; *n* = 17 for haplotype diversity) to meet assumptions, we found that the number of sequences per latitudinal band did not interfere with genetic diversity estimations (nucleotide diversity, *p* = 0.0644; haplotype diversity = 0.0654; data not shown).

We investigated the spatial relationship between genetic diversity and latitude by performing generalized linear mixed models (GLMM) comparing null, first‐, second‐, third‐, and fourth‐order polynomial models. We used latitude as the predictor variable, genetic diversity (nucleotide or haplotype diversity) as the response variable, and species as random factors. Because we compared different models, we used a maximum‐likelihood approach to estimate the parameters in the GLMMs (Fox et al. 2015). We used the Nelder–Mead optimization routine in our model to avoid convergence failure using the function control = lmerControl (optimizer = “Nelder_Mead”). Latitude was scaled (standard deviation = 1) and centered (mean = 0) before running the analysis to guarantee the independence of the predictor variables' terms in polynomial models (Schielzeth 2010). This analysis was run using the package *lme4* (Bates et al. 2015). Models (null, first, second, third, and fourth order) were compared under an information–theoretic approach using the Akaike information criterion (AIC). The model showing the lowest AIC and ∆AIC > 2 was selected as the best fit. In the case of more than one model having the lowest AICs and ∆AIC < 2, we opted for the less complex option. We reported marginal and conditional R^2^ for significant mixed models (R^2^_GLMM_—Nakagawa and Schielzeth 2013; Johnson 2014) implemented in the package *MuMIn* (Bartoń 2022). Marginal R^2^ represents the variance explained by fixed factors; conditional R^2^ represents the variance explained by both fixed and random factors. Bootstrapping was conducted to assess the stability and variability of the predictor variable estimates by calculating bias and standard error in the selected model. We used the bootMer function from the lme4 package to perform 1000 bootstrap replicates, extracting the predictor variable estimates using FUN = fixef.

The association between latitude and genetic diversity (nucleotide diversity or haplotype diversity) was also investigated within each species (intraspecific). No latitudinal bands were excluded from these analyses because we were focusing on species‐specific trends. We performed linear models and compared null, first‐, second‐, third‐, and fourth‐order polynomial regressions followed by AIC model comparison. Adjusted‐R^2^ is reported unless the best model was represented by the null model.

### 2.5 Environmental Variables Versus Genetic Diversity

We investigated the association between the six environmental variables mean sea surface temperature, dissolved oxygen, salinity, current velocity at mean depth, mean chlorophyll‐a, and pH versus genetic diversity by fitting GLMMs using environmental variables as the predictor variable, genetic diversity (nucleotide diversity or haplotype diversity) as the response variable, and species as the random factor. Environmental variables were scaled and centered before analyses. Multicollinearity among environmental variables was assessed using Pearson's correlation and variance inflation factor (VIF). Correlation coefficients > 0.7 and VIF > 5 were used to detect collinearity between environmental variables. Mean sea surface temperature and dissolved oxygen showed collinearity (inverse association), so we ran two different GLMMs excluding one of the variables in each set (Set 1: oxygen + salinity + current velocity + chlorophyll‐a + pH; Set 2: sea surface temperature + salinity + current velocity + chlorophyll‐a + pH). Sets 1 and 2 were compared using AIC values, and the lowest AIC and ∆AIC > 2 were selected as the best fit. In the case of the haplotype diversity analysis, models had ∆AIC < 2 (i.e., both showing good fit), so we moved forward using Sets 1 and 2 in this case. We employed the *dredge* function from the *MuMIn* package (Bartoń 2022) for model selection after detecting the best set of environmental variables. This function fits models for all possible subsets of the global model, allowing for automated selection across combinations of predictor variables (i.e., environmental variables). These models are then ranked according to their AICc values, with those having a ΔAICc < 2 being considered the most suitable for explaining the relationship between the response and predictor variables in the top models (Burnham and Anderson 2002). When multiple models had ΔAICc < 2, we applied the model.avg function to perform model averaging and assess the importance of each predictor based on Akaike weights (w). A predictor with a weight of *w* = 1 indicates its inclusion in all candidate models (Burnham and Anderson 2002). We calculated a confidence interval (95%) for variables included in the best models. This analysis was only calculated considering total genetic diversity because drivers of intraspecific patterns are beyond the scope and power of our dataset, as intraspecific results could be misguided by not all species having a large number of data points compared to the number of environmental variables tested.

All analyses were run in R ver.4.0.2.

## 3 Results

A second‐order polynomial model explains the nucleotide and haplotype diversity versus latitude relationship as a general trend in total genetic diversity analyses (Table 2 and Figure 1B,C). Nucleotide diversity is higher in lower latitudes, being slightly higher in the Caribbean region (5° N–15° N). Haplotype diversity is higher in lower latitudes (around the Equator). When looking at the estimates from bootstrapping for nucleotide diversity, we found a bias of 6.64401e‐05 and a standard error of 0.00199, indicating a reliable estimate; therefore, the bootstrap process did not substantially alter the original estimates. The estimates from bootstrapping for haplotype diversity showed a bias of 0.00395 and a standard error of 0.15170. Because the standard error is relatively large in this case, bootstrapping suggests some degree of uncertainty in this predictor variable estimate for haplotype diversity. In both cases, the combination of latitude and species (conditional‐R^2^) explains more of the results than only latitude (marginal‐R^2^).

**TABLE 2:** Results of the mixed models comparing null, first‐, second‐, third‐, and fourth‐order polynomial regressions—Latitude versus total mitochondrial genetic diversity.

Latitude								
	Model	df	AIC	Estimate	SE	*t*‐value	R^2^m	R^2^c
ND	Null	3	−1553.921					
	Linear	4	−1555.173					
	**Second order**	**5**	**−1559.18**	**−4.90E‐03**	**1.97E‐03**	**−2.488**	**0.01619947**	**0.747839**
	Third order	6	−1559.971					
	Fourth order	7	−1558.948					
HD	Null	3	−139.5476					
	Linear	4	−138.3875					
	**Second order**	**5**	**−143.0154**	**−0.3855**	**0.1469**	**−2.625**	**0.0382959**	**0.444783**
	Third order	6	−142.1394					
	Fourth order	7	−144.0229					

*Note:* Selected models are in bold. Parameters are only shown for the selected model.

Abbreviations: df, degrees of freedom; HD, haplotype diversity; ND, nucleotide diversity; R^2^c, conditional R^2^; R^2^m, marginal‐R^2^; SE, standard error.

**FIGURE 2:**
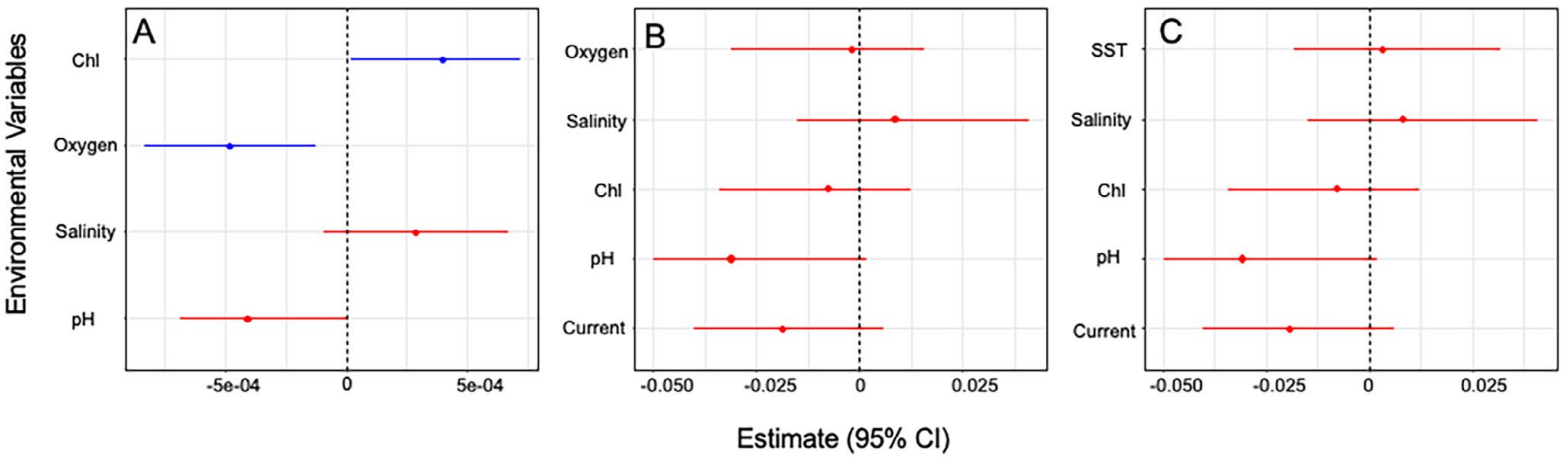
Results of association between environmental variables and (A) nucleotide diversity and (B, C) haplotype diversity. Two analyses were run for haplotype diversity to avoid fitting collinear variables in the same model (see methods for more details). Lines represent the estimate and 95% confidence interval. Red lines indicate confidence intervals that include zero, indicating that this variable potentially does not explain mitochondrial genetic diversity even if included in the best‐fitted model.

When investigating the association between environmental variables and genetic diversity, four models had the best fit (Set 2) in the case of nucleotide diversity. The contribution (weight, w) of the variables included in these models is as follows: dissolved oxygen (*w* = 1), chlorophyll‐a (*w* = 0.82), pH (*w* = 0.79), and salinity (*w* = 0.3). Although they were present in the top best models, only dissolved oxygen and chlorophyll‐a are the main environmental variables driving high total genetic diversity considering confidence intervals (Figure 2A). In the case of haplotype diversity, nine models had the best fit in both Sets 1 and 2. All variables were present in at least one of these nine models in each set, including a null model (no environmental variables). The contribution of each variable in Set 1 models was: pH (*w* = 0.8), current (*w* = 0.54), chlorophyll‐a (*w* = 0.19), salinity (*w* = 0.18), and sea surface temperature (*w* = 0.07). The contribution of each variable in Set 2 models was: pH (*w* = 0.8), current (*w* = 0.54), chlorophyll‐a (*w* = 0.19), salinity (*w* = 0.18), and dissolved oxygen (w = 0.08). In both cases (Set 1 and 2), the confidence interval for all variables crossed 0, indicating potentially no explanatory power (Figure 2B,C).

**FIGURE 3:**
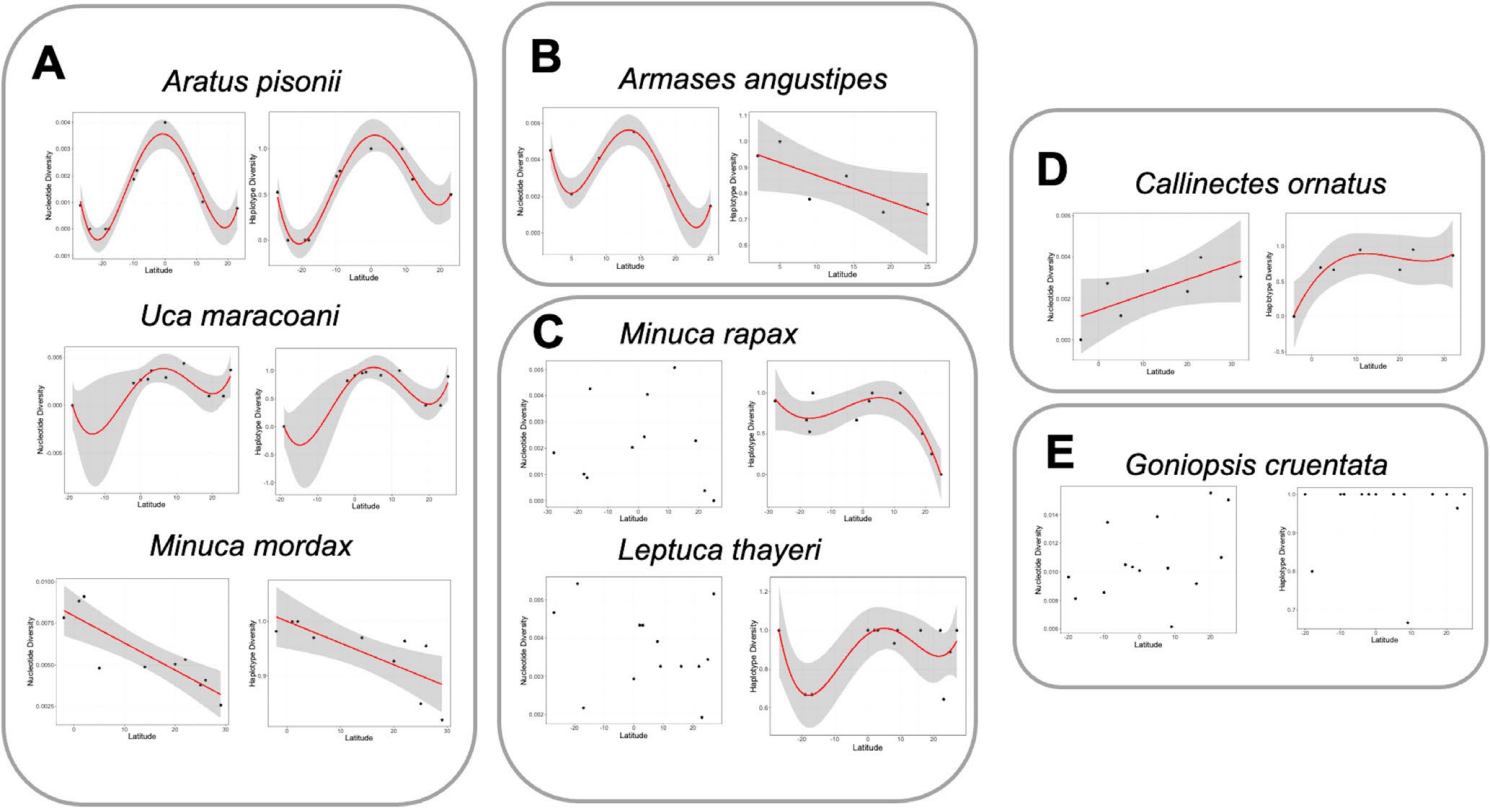
Examples of multiple scenarios found in intraspecific analyses testing the association between mitochondrial genetic diversity (nucleotide and haplotype diversity) versus latitude (A–E). (A) Higher genetic diversity in lower latitudes. (B) Mismatch between genetic diversity metrics: Nucleotide diversity depicts a combination of the latitudinal genetic diversity gradient with the central–marginal pattern; haplotype diversity depicts a latitudinal genetic diversity gradient. (C) Mismatch between genetic diversity metrics: Nucleotide diversity shows no association with latitude; haplotype diversity shows alternative trends. (D) Inverse latitudinal genetic diversity gradient. (E) No association between genetic diversity and latitude following the expectations of the marine species hypothesis. A trend line with standard error is indicated in all graphs.

We detected variable trends when looking at the association between genetic diversity and latitude at the intraspecific level. We selected eight of the 23 species to represent potential outcomes and discuss intraspecific genetic diversity explanations (latitudinal genetic diversity gradient, inverse latitudinal genetic diversity gradient, central–marginal hypothesis, and marine species hypothesis) and other findings (mismatch between nucleotide and haplotype diversity patterns) (Figures 3, S2 and Table S2). Most species fall within one of these scenarios, but discussing each species pattern is out of the scope of this paper. All results can be found in the Supplementary Files. *Aratus pisonii* and *Uca maracoani* show higher genetic diversity at the Equator (latitudinal genetic diversity gradient) but also higher genetic diversity in tropical regions outside of the Equator at the edge of the species distribution (following some marine species diversity gradient). *Minuca mordax* shows a latitudinal genetic diversity gradient when looking at both nucleotide and haplotype diversity. *Armases angustipes* nucleotide diversity peaks in the center of the species distribution (central–marginal hypothesis) and on the edges of the species distribution, but haplotype diversity shows higher genetic diversity in lower latitudes (latitudinal genetic diversity gradient). *Minuca rapax* shows contrasting patterns between nucleotide diversity (no association to latitude, following marine species hypothesis) and haplotype diversity (higher in the Caribbean and lower latitudes, declining in the South side of the species distribution. *Leptuca thayeri* also shows contrasting patterns between nucleotide diversity (no association to latitude, following marine species hypothesis) and haplotype diversity (similar to *A. pisonii* and *U. maracoani* ). *Callinectes ornatus* shows an inverse latitudinal genetic diversity gradient (higher genetic diversity in higher latitudes). *Goniopsis cruentata* follows what the marine species hypothesis expects, and no association is found between latitude and nucleotide/haplotype diversity.

## 4 Discussion

By targeting codistributed Tropical Western Atlantic marine decapods, our results showed a total genetic diversity spatial trend. After accounting for species variation in our models, total genetic diversity depicts a trend of increasing diversity at lower latitudes in both nucleotide and haplotype diversity datasets. Our model explanatory power is similar to values found in the association between latitude and mitochondrial genetic diversity in terrestrial invertebrates (Holoartic butterflies, Schär et al. 2017) and in marine vertebrates (global fishes, Clark and Pinsky 2024). At the species level, multiple scenarios emerged and will be discussed below. We highlight that total genetic diversity is not necessarily the result of the accumulation of intraspecific trends. Although similar mechanisms might act on the intra‐ and interspecific levels, one should not assume variation at different scales follows the same rules. Biological traits, demographic changes, and ecological interactions might drive genetic diversity spatial variation at both levels (Lawrence and Fraser 2020).

### 4.1 Total Genetic Diversity Latitudinal Gradient Can Be Explained by Environmental Factors

A latitudinal genetic diversity gradient is found in terrestrial and marine vertebrates (Hillebrand 2004; Adams and Hadly 2013; Miraldo et al. 2016; Manel et al. 2020). Here, we confirm high genetic diversity at lower latitudes across a marine invertebrate group (decapods). As mentioned before, the species diversity gradient sometimes extends to a genetic diversity gradient. No comprehensive study has investigated decapod species diversity distribution at a global scale, but this pattern has been found in specific regions or groups of decapods (Astorga et al. 2003; Fernández et al. 2009; Pappalardo and Fernández 2014; Sharifian et al. 2020; Teles et al. 2023). Therefore, species diversity and total genetic diversity seem to be connected in decapods, although further studies are necessary.

Temperature is frequently stated as the most important variable explaining latitudinal trends (Astorga et al. 2003; Tittensor et al. 2010; Manel et al. 2020), and the evolutionary speed hypothesis is a commonly evoked hypothesis to explain species diversity (Dowle et al. 2013; Mittelbach et al. 2007; Rohde 1992). Surprisingly, sea surface temperature was not detected as a main driver for total genetic diversity spatial distribution. A previous study looking at species, functional, and phylogenetic diversity of mangrove crabs along the Southwestern Atlantic also did not include this environmental variable as determining spatial trends (Teles et al. 2023). Many studies contradict the association between temperature and mitochondrial genetic diversity (Held 2001; Jetz et al. 2012; Jansson et al. 2013; Schluter and Pennell 2017; Rabosky et al. 2018; Orton et al. 2019). Similarly, recent genomic evidence also suggests that the mutation rate in the nuclear DNA is not correlated with temperature in many taxa (Liu et al. 2023). It is unclear whether temperature might not be an important factor for decapod genetic diversity in general, or if our results represent a taxa‐/region‐dependent result.

In our study, chlorophyll‐a had a positive association with total genetic diversity. Chlorophyll‐a is a proxy for ecosystem productivity; therefore, higher productivity areas can sustain large population sizes (energy richness hypothesis or more‐individuals hypothesis; Storch et al. 2018). Low‐latitude regions, which showed higher total genetic diversity in this study, are likely influenced by the discharge of the Amazon–Orinoco Plume. Phytoplankton biomass (indicated by chlorophyll concentration) is higher in zones near the plume, potentially due to the unique conditions caused by the outflow of nutrients and transition areas of high/low salinity and turbidity (Smith Jr and Demaster 1996). Additionally, the Amazon–Orinoco plume can interact with the North Brazil Current, creating gradients caused by differences in salinity and temperature between the plume and the oceanic current, which drives coastal upwelling, bringing nutrient‐rich deeper water to the surface (McCartney 1993). These scenarios support the energy‐richness hypothesis, which might be extended to genetic diversity. The neutral theory of molecular evolution suggests that genetic diversity is positively associated with the effective population size at neutral sites because of the mutation/drift equilibrium. Because effective population size can correlate to census size, high productivity areas could result in large population sizes (census size), which can also represent large effective population sizes; hence, higher genetic diversity (Wang et al. 2016). Chlorophyll‐a has also been found to be a critical variable in explaining mitochondrial genetic diversity in marine fishes, which might indicate that productivity is an important driver of diversity in marine animals (Clark and Pinsky 2024). Low‐latitude areas are also known to harbor high species diversity of primary producers (e.g., macroalgae; Keith et al. 2014). High species diversity areas can be associated with high genetic diversity in some species (Vellend and Geber 2005). In the case of seagrasses, meadows showing high genetic diversity have been associated with longevity, enhanced productivity, and increased numbers of invertebrates inhabiting these meadows (Reynolds et al. 2012). We suggest that this complex connection between primary producers' species and genetic diversity impacts ecosystem productivity, whose effects can cascade up the trophic network to other taxonomic groups in these regions promoting higher total genetic diversity. Another important variable was dissolved oxygen, which was negatively correlated with total genetic diversity. Low‐oxygen environments often create heterogeneous conditions (e.g., oxygen gradients), which promote a wider range of eco‐physiological strategies to persist in an environment, resulting in higher species diversity (physiological tolerance hypothesis; Currie et al. 2004, Herrera et al. 2023). These stressing conditions could lead to niche diversification, promoting species adapting to different microhabitats within a region and boosting genetic diversity, extending the effects of the physiological tolerance hypothesis from species diversity to total genetic diversity.

Although nucleotide and haplotype diversity show a latitudinal trend, the environmental variables tested could only explain nucleotide diversity variation. We believe this was caused by the two metrics representing different aspects of genetic diversity. As discussed, productivity and environmental gradients can correlate to more mutations in the mitochondrial DNA (detected by the nucleotide diversity), but not necessarily to these DNA sequences' abundance and the probability of their detection in a population (haplotype diversity). Regarding haplotype diversity, we have to consider that other environmental variables might be important for explaining this metric variation. Nonetheless, other factors can be involved in total genetic diversity variation, such as historical, biogeographical, and/or ecological processes (Mittelbach et al. 2007; Lawrence and Fraser 2020). Total genetic diversity may peak in lower latitudes because this region has had more time to evolve, accumulating genetic diversity across species. Simultaneously, the tropics cover a significant part of Earth's surface, and larger areas can support larger population sizes, which tend to show higher genetic diversity. Biotic interactions can also create opportunities for differentiation, leading to higher genetic diversity in tropical regions (Vellend and Geber 2005). Although commonly discussed at the intraspecific level, genetic diversity directly affects communities and ecosystems by influencing productivity, decomposition, pollination, and consumer–resource dynamics (Hughes et al. 2008). Further studies should explore if total genetic diversity can generate the same impact as intraspecific genetic diversity on local communities and ecosystems (Hughes et al. 2008).

### 4.2 Total Genetic Diversity is Not the Result of the Accumulation of Intraspecific Patterns

Although a total genetic diversity spatial trend has been detected, we found different trends at the intraspecific level. Our results indicate that species‐specific trends are variable and depict different latitude versus mitochondrial genetic diversity associations. Most species follow the marine species hypothesis, showing no spatial trend (e.g., *Goniopsis cruentata* , Figure 3E). As mentioned, larger population sizes and dispersal potential could be the factors behind this finding. However, we also detected higher genetic diversity in lower latitudes (e.g., *Aratus pisonii* , *Minuca mordax*, and *Uca maracoani* Figure 3A). *Armases angustipes*, a species restricted to the southern hemisphere, depicts different trends depending on the genetic diversity metric. Haplotype diversity follows a latitudinal genetic diversity gradient, but the nucleotide diversity pattern seems to be the result of a combination of central–marginal and latitudinal genetic diversity gradients, as suggested by Guo (2012) (Figure 3B). Again, this suggests a decoupling from diversity at the nucleotide level (e.g., number of mutations) and the sequence level (e.g., abundance of specific haplotypes and probability of sampling). This is also seen in *Leptuca thayeri* and *Minuca rapax*, but now haplotype diversity shows higher values in lower latitudes, and no pattern emerges when looking at nucleotide diversity (Figure 3C). Lastly, one of the species showed an inverse latitudinal genetic diversity gradient ( *Callinectes ornatus* , Figure 3D). This case might be related to species living near their upper‐temperature limit, so higher temperatures in lower latitudes can affect survivorship (Nguyen et al. 2011); consequently, population sizes can be smaller, resulting in lower genetic diversity.

Overall, no clear pattern explains why species do not share the same genetic diversity trend. These species occupy similar habitats (intertidal and shallow waters), have similar dispersal capacity (presence of larval stages), and some are from the same families as other species analyzed. Previous work has shown that crabs' mitochondrial genetic diversity at the species level is driven by historical demographic changes and life history traits (Peres and Mantelatto 2023). Specifically, higher mitochondrial genetic diversity is found in r‐strategist species that did not show signals of population size changes over time (Peres and Mantelatto 2023). Therefore, it is feasible to say that different spatial trends could emerge according to species‐specific biological traits and how they have been responding to demographic changes in specific regions (Ludt and Rocha 2015; Maggs et al. 2008; Toms et al. 2014). Simultaneously, physiology and ecological interactions (e.g., competition, predation) with other species could affect abundance in some sites (Cannicci et al. 2018; Menge 1995), affecting effective population size and, consequently, mitochondrial genetic diversity (Charlesworth 2009). We should notice that a few species show intraspecific genetic diversity peaks at the same region in which the total genetic diversity peak was detected, potentially indicating connections between intra‐ and interspecific trends. However, with our current dataset, it is hard to determine why only a few species showed this pattern. Ultimately, our results highlight that intraspecific trends can be variable even in codistributed and phylogenetically close groups, and that high genetic diversity in lower latitudes might not be the result of the combination of similar intraspecific trends. Also, we show that different diversity metrics can show different scenarios, suggesting caution when interpreting results.

## 5 Limitations

The connections between total genetic diversity (community) and intraspecific (population) genetic diversity are still unclear and should be the focus of future studies (Lawrence and Fraser 2020). As we previously mentioned, our results potentially indicate some type of connection in a few cases, and our dataset might be limited in the number of species and in the marker used to detect a clearer trend. Although many macrogenetics studies look at mitochondrial DNA genetic diversity (Miraldo et al. 2016; Manel et al. 2020; French et al. 2023; Fonseca et al. 2023; this study), we have to acknowledge this marker has specific limitations due to its nature. It is important to stress that our results are based on COI, and genome‐wide markers (e.g., SNPs) could reveal different trends. Unfortunately, this type of dataset for multiple tropical decapods (or other marine taxa) is lacking. We encourage future studies to explore latitudinal genomic diversity trends and drivers. Finally, our study is limited by the amount of genetic data available for decapods. Because many marine groups are the least studied taxa (Beheregaray 2008), ocean basin‐scale and taxon‐based studies can help us better appraise genetic diversity spatial patterns until more data are available.

## 6 Conclusions

General trends in total genetic diversity might result from various mechanisms acting upon higher levels of biological organization (e.g., community level, not species level) and deserve caution when interpreting macroscale results, as our data show. Further studies should investigate genetic diversity spatial patterns in other marine groups (including those that show inverse latitudinal species diversity patterns, like amphipods—Rivadeneira et al. 2011), different regions, use other molecular markers, and test the generality of our results to other marine species. Our study offers empirical results of genetic diversity spatial trends at different biological organization levels by analyzing all available populational‐level genetic data up to date for a major component of the marine fauna: the decapods. A macrogenetics approach combined with intraspecific analysis allowed us to reveal macroscale trends that are not apparent from intraspecific analyses. Therefore, our results highlight the complexity of genetic diversity spatial distribution and the potential interplay between ecological, historical, and environmental variables.

## Author Contributions

**Pedro A. Peres:** conceptualization (lead), data curation (equal), formal analysis (lead), investigation (equal), methodology (equal), project administration (equal), writing – original draft (lead), writing – review and editing (equal). **Fernando L. Mantelatto:** conceptualization (lead), data curation (equal), formal analysis (supporting), funding acquisition (lead), investigation (supporting), methodology (supporting), project administration (equal), resources (lead), supervision (lead), writing – original draft (supporting), writing – review and editing (equal).

## Ethics Statement

The authors have nothing to report.

## Consent

The authors have nothing to report.

## Conflicts of Interest

The authors declare no conflicts of interest.

## Code Availability

All R packages and software are mentioned in the main text.

## Supporting information


Appendix S1.

## Data Availability

Sequences used in this study are deposited in GenBank. Accession numbers and metadata are provided in the Supplemental Materials.
