# Supplementary material for: Macrogenetics Approach Reveals Spatial Trends and Drivers of Mitochondrial Genetic Diversity at Different Biological Organization Levels in Tropical Western Atlantic Decapods
Source: Ecol Evol. 2025 May 14;15(5):e71372. doi: 10.1002/ece3.71372 (PMC12077758; doi:10.1002/ece3.71372)
Supplement: Supplementary file 1 — Appendix S1. [file ECE3-15-e71372-s001.docx]

**Supplementary Material**

Macrogenetics approach reveal spatial trends and drivers of mitochondrial genetic diversity at different biological organization levels in Tropical Western Atlantic decapods

Pedro A. Peres; Fernando L. Mantelatto

Table S1. Genetic sequences used to investigate the association between latitude and mitochondrial genetic diversity in Tropical Western Atlantic decapods. GB acc. Number: GenBank accession number. Blank spaces: pending GenBank accession numbers.

| Species | GB acc. Number | Location | Latitude | Longitude |
| --- | --- | --- | --- | --- |
| Acanthonyx petiverii | KC695775 | Isla Margarita, Nueva Esparta, Venezuela | 10.6N | 64W |
| Acanthonyx petiverii | pending | Isla Margarita, Nueva Esparta, Venezuela | 10.6N | 64W |
| Acanthonyx petiverii | KC695771 | Isla Margarita, Nueva Esparta, Venezuela | 10.6N | 64W |
| Acanthonyx petiverii | KC695774 | Bocas Del Toro, Panamá | 9N | 82W |
| Acanthonyx petiverii | pending | Bocas Del Toro, Panamá | 9N | 82W |
| Acanthonyx petiverii | KC695776 | Playa de Cahuita, Costa Rica | 9N | 82W |
| Acanthonyx petiverii | pending | Ilhéus, Bahia, Brazil | 14S | 39W |
| Acanthonyx petiverii | pending | Ilhéus, Bahia, Brazil | 14S | 39W |
| Acanthonyx petiverii | pending | Ilhéus, Bahia, Brazil | 14S | 39W |
| Acanthonyx petiverii | pending | Ilhéus, Bahia, Brazil | 14S | 39W |
| Acanthonyx petiverii | pending | Ilhéus, Bahia, Brazil | 14S | 39W |
| Acanthonyx petiverii | pending | Ilhéus, Bahia, Brazil | 14S | 39W |
| Acanthonyx petiverii | KC695769 | Ilhéus, Bahia, Brazil | 14S | 39W |
| Acanthonyx petiverii | pending | Ilhéus, Bahia, Brazil | 14S | 39W |
| Acanthonyx petiverii | pending | Ilhéus, Bahia, Brazil | 14S | 39W |
| Acanthonyx petiverii | pending | Ilhéus, Bahia, Brazil | 14S | 39W |
| Acanthonyx petiverii | pending | Ilhéus, Bahia, Brazil | 14S | 39W |
| Acanthonyx petiverii | pending | Ilhéus, Bahia, Brazil | 14S | 39W |
| Acanthonyx petiverii | pending | Ilhéus, Bahia, Brazil | 14S | 39W |
| Acanthonyx petiverii | pending | Ilhéus, Bahia, Brazil | 14S | 39W |
| Acanthonyx petiverii | pending | Ilhéus, Bahia, Brazil | 14S | 39W |
| Acanthonyx petiverii | pending | Ubatuba, São Paulo, Brazil | 23S | 44W |
| Acanthonyx petiverii | pending | Ubatuba, São Paulo, Brazil | 23S | 44W |
| Acanthonyx petiverii | KC695768 | Ubatuba, São Paulo, Brazil | 23S | 44W |
| Acanthonyx petiverii | KC695772 | Ubatuba, São Paulo, Brazil | 23S | 44W |
| Acanthonyx petiverii | KC695770 | Ubatuba, São Paulo, Brazil | 23S | 44W |
| Acanthonyx petiverii | pending | Ubatuba, São Paulo, Brazil | 23S | 44W |
| Acanthonyx petiverii | pending | Ubatuba, São Paulo, Brazil | 23S | 44W |
| Acanthonyx petiverii | pending | Ubatuba, São Paulo, Brazil | 23S | 44W |
| Acanthonyx petiverii | pending | Ubatuba, São Paulo, Brazil | 23S | 44W |
| Acanthonyx petiverii | pending | Ubatuba, São Paulo, Brazil | 23S | 44W |
| Acanthonyx petiverii | KC695767 | Ubatuba, São Paulo, Brazil | 23S | 44W |
| Acanthonyx petiverii | pending | Ipojuca, Pernambuco, Brazil | 8S | 34W |
| Acanthonyx petiverii | pending | Recife, Pernambuco, Brazil | 8S | 34W |
| Acanthonyx petiverii | pending | Recife, Pernambuco, Brazil | 8S | 34W |
| Acanthonyx petiverii | pending | Recife, Pernambuco, Brazil | 8S | 34W |
| Acanthonyx petiverii | pending | Recife, Pernambuco, Brazil | 8S | 34W |
| Acanthonyx petiverii | pending | Recife, Pernambuco, Brazil | 8S | 34W |
| Acanthonyx petiverii | KC695773 | Recife, Pernambuco, Brazil | 8S | 34W |
| Acanthonyx petiverii | pending | Recife, Pernambuco, Brazil | 8S | 34W |
| Acanthonyx petiverii | pending | Recife, Pernambuco, Brazil | 8S | 34W |
| Aratus pisonii | KY964577 | Isla Margarita, Nueva Esparta, Venezuela | 10.6N | 64W |
| Aratus pisonii | KU313043 | Isla Margarita, Nueva Esparta, Venezuela | 10.6N | 64W |
| Aratus pisonii | KU313048 | Trinidad, Trinidad and Tobago | 10N | 61W |
| Aratus pisonii | KU313046 | Trinidad, Trinidad and Tobago | 10N | 61W |
| Aratus pisonii | KU313044 | Trinidad, Trinidad and Tobago | 10N | 61W |
| Aratus pisonii | KU313047 | Trinidad, Trinidad and Tobago | 10N | 61W |
| Aratus pisonii | KU313045 | Trinidad, Trinidad and Tobago | 10N | 61W |
| Aratus pisonii | MK783687 | Catemaco, Veracruz, Mexico | 18.6N | 95W |
| Aratus pisonii | MK783686 | Catemaco, Veracruz, Mexico | 18.6N | 95W |
| Aratus pisonii | KU313028 | Catemaco, Veracruz, Mexico | 18.6N | 95W |
| Aratus pisonii | MK783683 | Campeche, Campeche, Mexico | 19.5N | 91W |
| Aratus pisonii | KU313030 | Campeche, Campeche, Mexico | 19.5N | 91W |
| Aratus pisonii | KU313031 | Champotón, Campeche, Mexico | 19.5N | 91W |
| Aratus pisonii | KU313027 | Key West, Florida, USA | 24N | 81W |
| Aratus pisonii | MK783682 | Pigeon Key, Florida, USA | 24N | 81W |
| Aratus pisonii | MK783681 | Pigeon Key, Florida, USA | 24N | 81W |
| Aratus pisonii | KY964581 | Pigeon Key, Florida, USA | 24N | 81W |
| Aratus pisonii | KU313026 | Pigeon Key, Florida, USA | 24N | 81W |
| Aratus pisonii | KU313020 | Fort Pierce, Florida, USA | 27N | 80W |
| Aratus pisonii | KU313019 | Fort Pierce, Florida, USA | 27N | 80W |
| Aratus pisonii | KU313022 | Fort Pierce, Florida, USA | 27N | 80W |
| Aratus pisonii | KU313021 | Fort Pierce, Florida, USA | 27N | 80W |
| Aratus pisonii | KY964580 | Tampa, Florida, USA | 27N | 82W |
| Aratus pisonii | KY964579 | Tampa, Florida, USA | 27N | 82W |
| Aratus pisonii | KY964578 | Tampa, Florida, USA | 27N | 82W |
| Aratus pisonii | MN184025 | Bocas Del Toro, Panamá | 9N | 82W |
| Aratus pisonii | KU313042 | Bocas Del Toro, Panamá | 9N | 82W |
| Aratus pisonii | KU313041 | Bocas Del Toro, Panamá | 9N | 82W |
| Aratus pisonii | KU313040 | Bocas Del Toro, Panamá | 9N | 82W |
| Aratus pisonii | KU313039 | Bocas Del Toro, Panamá | 9N | 82W |
| Aratus pisonii | KU313038 | Bocas Del Toro, Panamá | 9N | 82W |
| Aratus pisonii | KU313033 | Laguna de Gandoca, Costa Rica | 9N | 82W |
| Aratus pisonii | KU313034 | Laguna de Gandoca, Costa Rica | 9N | 82W |
| Aratus pisonii | KU313037 | Laguna de Gandoca, Costa Rica | 9N | 82W |
| Aratus pisonii | MK783689 | Laguna de Gandoca, Costa Rica | 9N | 82W |
| Aratus pisonii | MK783688 | Laguna de Gandoca, Costa Rica | 9N | 82W |
| Aratus pisonii | KU313036 | Laguna de Gandoca, Costa Rica | 9N | 82W |
| Aratus pisonii | KU313035 | Laguna de Gandoca, Costa Rica | 9N | 82W |
| Aratus pisonii | KU313055 | Salinópolis, Pará, Brazil | 0 | 47W |
| Aratus pisonii | KU313054 | Salinópolis, Pará, Brazil | 0 | 47W |
| Aratus pisonii | KU313052 | Salinópolis, Pará, Brazil | 0 | 47W |
| Aratus pisonii | KU313051 | Salinópolis, Pará, Brazil | 0 | 47W |
| Aratus pisonii | KU313053 | Salinópolis, Pará, Brazil | 0 | 47W |
| Aratus pisonii | KU313063 | Porto do Sauípe, Bahia, Brazil | 12S | 37W |
| Aratus pisonii | KU313062 | Porto do Sauípe, Bahia, Brazil | 12S | 37W |
| Aratus pisonii | KU313061 | Porto do Sauípe, Bahia, Brazil | 12S | 37W |
| Aratus pisonii | KU313081 | Guarujá, São Paulo, Brazil | 23.46S | 45.58W |
| Aratus pisonii | KU313076 | Ubatuba, São Paulo, Brazil | 23S | 44W |
| Aratus pisonii | KU313083 | Guarujá, São Paulo, Brazil | 23.46S | 45.58W |
| Aratus pisonii | KU313082 | Guarujá, São Paulo, Brazil | 23.46S | 45.58W |
| Aratus pisonii | KU313080 | Guarujá, São Paulo, Brazil | 23.46S | 45.58W |
| Aratus pisonii | KU313071 | Paraty, Rio de Janeiro, Brazil | 23S | 44W |
| Aratus pisonii | KU313072 | Paraty, Rio de Janeiro, Brazil | 23S | 44W |
| Aratus pisonii | KU313070 | Paraty, Rio de Janeiro, Brazil | 23S | 44W |
| Aratus pisonii | KU313079 | Ubatuba, São Paulo, Brazil | 23S | 44W |
| Aratus pisonii | KU313078 | Ubatuba, São Paulo, Brazil | 23S | 44W |
| Aratus pisonii | KU313075 | Ubatuba, São Paulo, Brazil | 23S | 44W |
| Aratus pisonii | KU313077 | Ubatuba, São Paulo, Brazil | 23S | 44W |
| Aratus pisonii | KU313060 | Passo do Camaragibe, Alagoas, Brazil | 9S | 35W |
| Aratus pisonii | KU313058 | Passo do Camaragibe, Alagoas, Brazil | 9S | 35W |
| Aratus pisonii | KU313059 | Passo do Camaragibe, Alagoas, Brazil | 9S | 35W |
| Arenaeus cribrarius | KU905836 | Chesapeake Bay, Virginia, USA | 36N | 76W |
| Arenaeus cribrarius | MH087567 | Chesapeake Bay, Virginia, USA | 36N | 76W |
| Arenaeus cribrarius | MH087581 | Chesapeake Bay, Virginia, USA | 36N | 76W |
| Arenaeus cribrarius | MH087696 | Chesapeake Bay, Virginia, USA | 36N | 76W |
| Arenaeus cribrarius | JX123429 | Macaé, Rio de Janeiro, Brazil | 22S | 41W |
| Arenaeus cribrarius | pending | Macaé, Rio de Janeiro, Brazil | 22S | 41W |
| Arenaeus cribrarius | pending | Macaé, Rio de Janeiro, Brazil | 22S | 41W |
| Arenaeus cribrarius | pending | Macaé, Rio de Janeiro, Brazil | 22S | 41W |
| Arenaeus cribrarius | pending | Macaé, Rio de Janeiro, Brazil | 22S | 41W |
| Arenaeus cribrarius | pending | Macaé, Rio de Janeiro, Brazil | 22S | 41W |
| Arenaeus cribrarius | pending | Ubatuba, São Paulo, Brazil | 23S | 44W |
| Arenaeus cribrarius | JX123434 | Ubatuba, São Paulo, Brazil | 23S | 44W |
| Arenaeus cribrarius | JX123432 | Ubatuba, São Paulo, Brazil | 23S | 44W |
| Arenaeus cribrarius | JX123433 | Ubatuba, São Paulo, Brazil | 23S | 44W |
| Arenaeus cribrarius | JX123431 | Ubatuba, São Paulo, Brazil | 23S | 44W |
| Arenaeus cribrarius | JX123435 | Ubatuba, São Paulo, Brazil | 23S | 44W |
| Arenaeus cribrarius | pending | Ubatuba, São Paulo, Brazil | 23S | 44W |
| Arenaeus cribrarius | pending | Ubatuba, São Paulo, Brazil | 23S | 44W |
| Arenaeus cribrarius | pending | Ubatuba, São Paulo, Brazil | 23S | 44W |
| Arenaeus cribrarius | JX123436 | Ubatuba, São Paulo, Brazil | 23S | 44W |
| Arenaeus cribrarius | JX123430 | Ubatuba, São Paulo, Brazil | 23S | 44W |
| Arenaeus cribrarius | JX123438 | Ilha Comprida, São Paulo, Brazil | 24.7S | 47.5W |
| Arenaeus cribrarius | pending | Ilha comprida, São Paulo, Brazil | 24.7S | 47.5W |
| Arenaeus cribrarius | JX123439 | Ilha Comprida, São Paulo, Brazil | 24.7S | 47.5W |
| Arenaeus cribrarius | JX123437 | Peruíbe, São Paulo, Brazil | 24S | 46W |
| Arenaeus cribrarius | pending | Peruíbe, São Paulo, Brazil | 24S | 46W |
| Arenaeus cribrarius | JX123441 | Cananéia, São Paulo, Brazil | 25S | 47W |
| Arenaeus cribrarius | JX123442 | Cananéia, São Paulo, Brazil | 25S | 47W |
| Arenaeus cribrarius | pending | Cananéia, São Paulo, Brazil | 25S | 47W |
| Arenaeus cribrarius | pending | Cananéia, São Paulo, Brazil | 25S | 47W |
| Arenaeus cribrarius | JX123440 | Cananéia, São Paulo, Brazil | 25S | 47W |
| Arenaeus cribrarius | pending | Cananéia, São Paulo, Brazil | 25S | 47W |
| Arenaeus cribrarius | pending | Cananéia, São Paulo, Brazil | 25S | 47W |
| Arenaeus cribrarius | JX123444 | São Francisco do Sul, Santa Catarina, Brazil | 26S | 48W |
| Arenaeus cribrarius | JX123445 | São Francisco do Sul, Santa Catarina, Brazil | 26S | 48W |
| Arenaeus cribrarius | pending | São Francisco do Sul, Santa Catarina, Brazil | 26S | 48W |
| Arenaeus cribrarius | pending | São Francisco do Sul, Santa Catarina, Brazil | 26S | 48W |
| Arenaeus cribrarius | pending | Parnamirim, Rio Grande do Norte, Brazil | 5S | 35W |
| Arenaeus cribrarius | JX123426 | Parnamirim, Rio Grande do Norte, Brazil | 5S | 35W |
| Arenaeus cribrarius | JX123427 | Parnamirim, Rio Grande do Norte, Brazil | 5S | 35W |
| Arenaeus cribrarius | JX123428 | Maceió, Alagoas, Brazil | 9S | 35W |
| Arenaeus cribrarius | pending | Maragogi, Alagoas, Brazil | 9S | 35W |
| Arenaeus cribrarius | pending | Maragogi, Alagoas, Brazil | 9S | 35W |
| Arenaeus cribrarius | pending | Maragogi, Alagoas, Brazil | 9S | 35W |
| Armases angustipes | KX906637 | Ilhéus, Bahia, Brazil | 14S | 39W |
| Armases angustipes | KX906641 | Ilhéus, Bahia, Brazil | 14S | 39W |
| Armases angustipes | KX906645 | Ilhéus, Bahia, Brazil | 14S | 39W |
| Armases angustipes | KX906646 | Ilhéus, Bahia, Brazil | 14S | 39W |
| Armases angustipes | KX906640 | Ilhéus, Bahia, Brazil | 14S | 39W |
| Armases angustipes | KX906644 | Ilhéus, Bahia, Brazil | 14S | 39W |
| Armases angustipes | KX906647 | Ilhéus, Bahia, Brazil | 14S | 39W |
| Armases angustipes | KX906638 | Ilhéus, Bahia, Brazil | 14S | 39W |
| Armases angustipes | KX906642 | Ilhéus, Bahia, Brazil | 14S | 39W |
| Armases angustipes | KX906639 | Ilhéus, Bahia, Brazil | 14S | 39W |
| Armases angustipes | KX906643 | Ilhéus, Bahia, Brazil | 14S | 39W |
| Armases angustipes | KX906648 | Aracruz, Espírito Santo, Brazil | 19S | 39W |
| Armases angustipes | KX906649 | Aracruz, Espírito Santo, Brazil | 19S | 39W |
| Armases angustipes | KX906651 | Aracruz, Espírito Santo, Brazil | 19S | 39W |
| Armases angustipes | KX906652 | Aracruz, Espírito Santo, Brazil | 19S | 39W |
| Armases angustipes | KX906655 | Aracruz, Espírito Santo, Brazil | 19S | 39W |
| Armases angustipes | KX906657 | Aracruz, Espírito Santo, Brazil | 19S | 39W |
| Armases angustipes | KX906650 | Aracruz, Espírito Santo, Brazil | 19S | 39W |
| Armases angustipes | KX906653 | Aracruz, Espírito Santo, Brazil | 19S | 39W |
| Armases angustipes | KX906654 | Aracruz, Espírito Santo, Brazil | 19S | 39W |
| Armases angustipes | KX906658 | Aracruz, Espírito Santo, Brazil | 19S | 39W |
| Armases angustipes | KX906656 | Aracruz, Espírito Santo, Brazil | 19S | 39W |
| Armases angustipes | KX906660 | Guaratuba, Paraná, Brazil | 25S | 48W |
| Armases angustipes | KX906663 | Guaratuba, Paraná, Brazil | 25S | 48W |
| Armases angustipes | KX906667 | Guaratuba, Paraná, Brazil | 25S | 48W |
| Armases angustipes | KX906668 | Guaratuba, Paraná, Brazil | 25S | 48W |
| Armases angustipes | KX906669 | Guaratuba, Paraná, Brazil | 25S | 48W |
| Armases angustipes | KX906670 | Guaratuba, Paraná, Brazil | 25S | 48W |
| Armases angustipes | KX906661 | Guaratuba, Paraná, Brazil | 25S | 48W |
| Armases angustipes | KX906666 | Guaratuba, Paraná, Brazil | 25S | 48W |
| Armases angustipes | KX906665 | Guaratuba, Paraná, Brazil | 25S | 48W |
| Armases angustipes | KX906664 | Guaratuba, Paraná, Brazil | 25S | 48W |
| Armases angustipes | KX906671 | Guaratuba, Paraná, Brazil | 25S | 48W |
| Armases angustipes | KX906662 | Guaratuba, Paraná, Brazil | 25S | 48W |
| Armases angustipes | KX906614 | São Luís, Maranhão, Brazil | 2S | 44W |
| Armases angustipes | KX906615 | São Luís, Maranhão, Brazil | 2S | 44W |
| Armases angustipes | KX906611 | São Luís, Maranhão, Brazil | 2S | 44W |
| Armases angustipes | KX906612 | São Luís, Maranhão, Brazil | 2S | 44W |
| Armases angustipes | KX906613 | São Luís, Maranhão, Brazil | 2S | 44W |
| Armases angustipes | KX906608 | São Luís, Maranhão, Brazil | 2S | 44W |
| Armases angustipes | KX906659 | São Luís, Maranhão, Brazil | 2S | 44W |
| Armases angustipes | KX906607 | São Luís, Maranhão, Brazil | 2S | 44W |
| Armases angustipes | KX906610 | São Luís, Maranhão, Brazil | 2S | 44W |
| Armases angustipes | KX906606 | São Luís, Maranhão, Brazil | 2S | 44W |
| Armases angustipes | KX906609 | São Luís, Maranhão, Brazil | 2S | 44W |
| Armases angustipes | KX906621 | Natal, Rio Grande do Norte, Brazil | 5S | 35W |
| Armases angustipes | KX906617 | Natal, Rio Grande do Norte, Brazil | 5S | 35W |
| Armases angustipes | KX906616 | Natal, Rio Grande do Norte, Brazil | 5S | 35W |
| Armases angustipes | KX906618 | Natal, Rio Grande do Norte, Brazil | 5S | 35W |
| Armases angustipes | KX906619 | Natal, Rio Grande do Norte, Brazil | 5S | 35W |
| Armases angustipes | KX906620 | Natal, Rio Grande do Norte, Brazil | 5S | 35W |
| Armases angustipes | KX906622 | Natal, Rio Grande do Norte, Brazil | 5S | 35W |
| Armases angustipes | KX906624 | Natal, Rio Grande do Norte, Brazil | 5S | 35W |
| Armases angustipes | KX906625 | Natal, Rio Grande do Norte, Brazil | 5S | 35W |
| Armases angustipes | KX906626 | Natal, Rio Grande do Norte, Brazil | 5S | 35W |
| Armases angustipes | KX906623 | Natal, Rio Grande do Norte, Brazil | 5S | 35W |
| Armases angustipes | KX906627 | Maceió, Alagoas, Brazil | 9S | 35W |
| Armases angustipes | KX906629 | Maceió, Alagoas, Brazil | 9S | 35W |
| Armases angustipes | KX906632 | Maceió, Alagoas, Brazil | 9S | 35W |
| Armases angustipes | KX906633 | Maceió, Alagoas, Brazil | 9S | 35W |
| Armases angustipes | KX906634 | Maceió, Alagoas, Brazil | 9S | 35W |
| Armases angustipes | KX906628 | Maceió, Alagoas, Brazil | 9S | 35W |
| Armases angustipes | KX906630 | Maceió, Alagoas, Brazil | 9S | 35W |
| Armases angustipes | KX906636 | Maceió, Alagoas, Brazil | 9S | 35W |
| Armases angustipes | KX906631 | Maceió, Alagoas, Brazil | 9S | 35W |
| Armases angustipes | KX906635 | Maceió, Alagoas, Brazil | 9S | 35W |
| Callinectes danae | MN184215 | Bocas Del Toro, Panama | 9N | 82W |
| Callinectes danae | KY940213 | Bocas Del Toro, Panama | 9N | 82W |
| Callinectes danae | MN183834 | Bocas Del Toro, Panama | 9N | 82W |
| Callinectes danae | MN184091 | Bocas Del Toro, Panama | 9N | 82W |
| Callinectes danae | MN184052 | Bocas Del Toro, Panama | 9N | 82W |
| Callinectes danae | MN184036 | Bocas Del Toro, Panama | 9N | 82W |
| Callinectes danae | KY940183 | Salvador, Bahia, Brazil | 12S | 37W |
| Callinectes danae | KY940181 | Salvador, Bahia, Brazil | 12S | 37W |
| Callinectes danae | KY940182 | Salvador, Bahia, Brazil | 12S | 37W |
| Callinectes danae | KY940155 | Salvador, Bahia, Brazil | 12S | 37W |
| Callinectes danae | KY940184 | Salvador, Bahia, Brazil | 12S | 37W |
| Callinectes danae | KY940168 | Vitória, Espirito Santo, Brazil | 20S | 40W |
| Callinectes danae | KY940170 | Vitória, Espirito Santo, Brazil | 20S | 40W |
| Callinectes danae | KY940171 | Vitória, Espirito Santo, Brazil | 20S | 40W |
| Callinectes danae | KY940167 | Vitória, Espirito Santo, Brazil | 20S | 40W |
| Callinectes danae | KY940169 | Vitória, Espirito Santo, Brazil | 20S | 40W |
| Callinectes danae | KY940202 | Rio de Janeiro, Rio de Janeiro, Brazil | 22S | 43W |
| Callinectes danae | KY940199 | Rio de Janeiro, Rio de Janeiro, Brazil | 22S | 43W |
| Callinectes danae | KY940200 | Rio de Janeiro, Rio de Janeiro, Brazil | 22S | 43W |
| Callinectes danae | KY940198 | Rio de Janeiro, Rio de Janeiro, Brazil | 22S | 43W |
| Callinectes danae | KY940201 | Rio de Janeiro, Rio de Janeiro, Brazil | 22S | 43W |
| Callinectes danae | MF490073 | Ubatuba, São Paulo, Brazil | 23S | 44W |
| Callinectes danae | KY940147 | Ubatuba, São Paulo, Brazil | 23S | 44W |
| Callinectes danae | KY940148 | Ubatuba, São Paulo, Brazil | 23S | 44W |
| Callinectes danae | KY940149 | Ubatuba, São Paulo, Brazil | 23S | 44W |
| Callinectes danae | KY940216 | Ubatuba, São Paulo, Brazil | 23S | 44W |
| Callinectes danae | KY940150 | Cananéia, São Paulo, Brazil | 25S | 48W |
| Callinectes danae | KY940152 | Cananéia, São Paulo, Brazil | 25S | 48W |
| Callinectes danae | KY940153 | Cananéia, São Paulo, Brazil | 25S | 48W |
| Callinectes danae | KY940154 | Cananéia, São Paulo, Brazil | 25S | 48W |
| Callinectes danae | KY940174 | Pontal do Paraná, Paraná, Brazil | 25S | 48W |
| Callinectes danae | KY940175 | Pontal do Paraná, Paraná, Brazil | 25S | 48W |
| Callinectes danae | KY940176 | Pontal do Paraná, Paraná, Brazil | 25S | 48W |
| Callinectes danae | KY940177 | Pontal do Paraná, Paraná, Brazil | 25S | 48W |
| Callinectes danae | KY940178 | Pontal do Paraná, Paraná, Brazil | 25S | 48W |
| Callinectes danae | KY940179 | Pontal do Paraná, Paraná, Brazil | 25S | 48W |
| Callinectes danae | KY940151 | Cananéia, São Paulo, Brazil | 25S | 48W |
| Callinectes danae | pending | São José do Ribamar, Maranhão, Brazil | 2S | 44W |
| Callinectes danae | pending | São José do Ribamar, Maranhão, Brazil | 2S | 44W |
| Callinectes danae | pending | São José do Ribamar, Maranhão, Brazil | 2S | 44W |
| Callinectes danae | pending | São José do Ribamar, Maranhão, Brazil | 2S | 44W |
| Callinectes danae | pending | São José do Ribamar, Maranhão, Brazil | 2S | 44W |
| Callinectes danae | KY940162 | Rio Grande, Rio Grande do Sul, Brazil | 32S | 52W |
| Callinectes danae | KY940165 | Rio Grande, Rio Grande do Sul, Brazil | 32S | 52W |
| Callinectes danae | KY940166 | Rio Grande, Rio Grande do Sul, Brazil | 32S | 52W |
| Callinectes danae | KY940164 | Rio Grande, Rio Grande do Sul, Brazil | 32S | 52W |
| Callinectes danae | KY940163 | Rio Grande, Rio Grande do Sul, Brazil | 32S | 52W |
| Callinectes danae | KY940203 | Rio Grande, Rio Grande do Sul, Brazil | 32S | 52W |
| Callinectes danae | KY940173 | Ipojuca, Pernambuco, Brazil | 8S | 34W |
| Callinectes danae | KY940187 | Ipojuca, Pernambuco, Brazil | 8S | 34W |
| Callinectes danae | KY940185 | Ipojuca, Pernambuco, Brazil | 8S | 34W |
| Callinectes danae | KY940186 | Ipojuca, Pernambuco, Brazil | 8S | 34W |
| Callinectes danae | KY940188 | Ipojuca, Pernambuco, Brazil | 8S | 34W |
| Callinectes danae | KY940172 | Ipojuca, Pernambuco, Brazil | 8S | 34W |
| Callinectes ornatus | MN811213 | Ile Saint-Joseph, French Guiana | 4.7N | 52W |
| Callinectes ornatus | MN811214 | Ile Saint-Joseph, French Guiana | 4.7N | 52W |
| Callinectes ornatus | MN811215 | Ile Saint-Joseph, French Guiana | 4.7N | 52W |
| Callinectes ornatus | MT272140 | Aracajú, Sergipe, Brazil | 11S | 37W |
| Callinectes ornatus | MT272141 | Aracajú, Sergipe, Brazil | 11S | 37W |
| Callinectes ornatus | MT272148 | Aracajú, Sergipe, Brazil | 11S | 37W |
| Callinectes ornatus | MT272154 | Aracajú, Sergipe, Brazil | 11S | 37W |
| Callinectes ornatus | MT272161 | Aracajú, Sergipe, Brazil | 11S | 37W |
| Callinectes ornatus | MT272162 | Aracajú, Sergipe, Brazil | 11S | 37W |
| Callinectes ornatus | MT272163 | Aracajú, Sergipe, Brazil | 11S | 37W |
| Callinectes ornatus | MT272137 | Vitória, Espirito Santo, Brazil | 20S | 40W |
| Callinectes ornatus | MT272138 | Vitória, Espirito Santo, Brazil | 20S | 40W |
| Callinectes ornatus | MT272169 | Vitória, Espirito Santo, Brazil | 20S | 40W |
| Callinectes ornatus | MT272134 | Ubatuba, São Paulo, Brazil | 23S | 44W |
| Callinectes ornatus | MT272135 | Ubatuba, São Paulo, Brazil | 23S | 44W |
| Callinectes ornatus | MT272136 | Ubatuba, São Paulo, Brazil | 23S | 44W |
| Callinectes ornatus | MT272147 | Ubatuba, São Paulo, Brazil | 23S | 44W |
| Callinectes ornatus | MT272153 | Ubatuba, São Paulo, Brazil | 23S | 44W |
| Callinectes ornatus | MT272164 | Ubatuba, São Paulo, Brazil | 23S | 44W |
| Callinectes ornatus | MT272165 | Ubatuba, São Paulo, Brazil | 23S | 44W |
| Callinectes ornatus | MT272159 | Ubatuba, São Paulo, Brazil | 23S | 44W |
| Callinectes ornatus | MT272160 | Ubatuba, São Paulo, Brazil | 23S | 44W |
| Callinectes ornatus | MF490074 | Ubatuba, São Paulo, Brazil | 23S | 44W |
| Callinectes ornatus | MT272167 | Ubatuba, São Paulo, Brazil | 23S | 44W |
| Callinectes ornatus | MT272168 | Ubatuba, São Paulo, Brazil | 23S | 44W |
| Callinectes ornatus | OP817087 | São José do Ribamar, Maranhão, Brazil | 2S | 44W |
| Callinectes ornatus | OP917090 | São José do Ribamar, Maranhão, Brazil | 2S | 44W |
| Callinectes ornatus | OP817088 | São José do Ribamar, Maranhão, Brazil | 2S | 44W |
| Callinectes ornatus | OP817086 | São José do Ribamar, Maranhão, Brazil | 2S | 44W |
| Callinectes ornatus | OP817089 | São José do Ribamar, Maranhão, Brazil | 2S | 44W |
| Callinectes ornatus | MT272142 | Rio Grande, Rio Grande do Sul, Brazil | 32S | 52W |
| Callinectes ornatus | MT272143 | Rio Grande, Rio Grande do Sul, Brazil | 32S | 52W |
| Callinectes ornatus | MT272144 | Rio Grande, Rio Grande do Sul, Brazil | 32S | 52W |
| Callinectes ornatus | MT272145 | Rio Grande, Rio Grande do Sul, Brazil | 32S | 52W |
| Callinectes ornatus | MT272149 | Rio Grande, Rio Grande do Sul, Brazil | 32S | 52W |
| Callinectes ornatus | MT272150 | Rio Grande, Rio Grande do Sul, Brazil | 32S | 52W |
| Callinectes ornatus | MT272155 | Rio Grande, Rio Grande do Sul, Brazil | 32S | 52W |
| Callinectes ornatus | MT272156 | Rio Grande, Rio Grande do Sul, Brazil | 32S | 52W |
| Callinectes ornatus | MT272157 | Rio Grande, Rio Grande do Sul, Brazil | 32S | 52W |
| Callinectes ornatus | MT272166 | Rio Grande, Rio Grande do Sul, Brazil | 32S | 52W |
| Callinectes ornatus | MT272170 | Rio Grande, Rio Grande do Sul, Brazil | 32S | 52W |
| Callinectes ornatus | MT272139 | Parnamirim, Rio Grande do Norte, Brazil | 5S | 35W |
| Callinectes ornatus | KY940161 | Parnamirim, Rio Grande do Norte, Brazil | 5S | 35W |
| Callinectes ornatus | MT272146 | Parnamirim, Rio Grande do Norte, Brazil | 5S | 35W |
| Eriphia gonagra | pending | Trinidad, Trinidad and Tobago | 10N | 61W |
| Eriphia gonagra | pending | Trinidad, Trinidad and Tobago | 10N | 61W |
| Eriphia gonagra | pending | Trinidad, Trinidad and Tobago | 10N | 61W |
| Eriphia gonagra | pending | Trinidad, Trinidad and Tobago | 10N | 61W |
| Eriphia gonagra | pending | Trinidad, Trinidad and Tobago | 10N | 61W |
| Eriphia gonagra | MN183988 | Bocas Del Toro, Panama | 9N | 82W |
| Eriphia gonagra | pending | Bocas Del Toro, Panama | 9N | 82W |
| Eriphia gonagra | MN184079 | Bocas Del Toro, Panama | 9N | 82W |
| Eriphia gonagra | MN183856 | Bocas Del Toro, Panama | 9N | 82W |
| Eriphia gonagra | pending | Salvador, Bahia, Brazil | 12S | 37W |
| Eriphia gonagra | pending | Salvador, Bahia, Brazil | 12S | 37W |
| Eriphia gonagra | pending | Salvador, Bahia, Brazil | 12S | 37W |
| Eriphia gonagra | pending | Salvador, Bahia, Brazil | 12S | 37W |
| Eriphia gonagra | pending | Ubatuba, São Paulo, Brazil | 23S | 44W |
| Eriphia gonagra | pending | Ubatuba, São Paulo, Brazil | 23S | 44W |
| Eriphia gonagra | MF490083 | Ubatuba, São Paulo, Brazil | 23S | 44W |
| Eriphia gonagra | pending | Ubatuba, São Paulo, Brazil | 23S | 44W |
| Eriphia gonagra | pending | Ubatuba, São Paulo, Brazil | 23S | 44W |
| Eriphia gonagra | pending | Ubatuba, São Paulo, Brazil | 23S | 44W |
| Eriphia gonagra | pending | Ubatuba, São Paulo, Brazil | 23S | 44W |
| Eriphia gonagra | pending | Ubatuba, São Paulo, Brazil | 23S | 44W |
| Eriphia gonagra | pending | Natal, Rio Grande do Norte, Brazil | 5S | 35W |
| Eriphia gonagra | pending | Natal, Rio Grande do Norte, Brazil | 5S | 35W |
| Eriphia gonagra | pending | Natal, Rio Grande do Norte, Brazil | 5S | 35W |
| Eriphia gonagra | pending | Natal, Rio Grande do Norte, Brazil | 5S | 35W |
| Eriphia gonagra | pending | Gaibu, Pernambuco, Brazil | 8S | 34W |
| Eriphia gonagra | pending | Gaibu, Pernambuco, Brazil | 8S | 34W |
| Eriphia gonagra | pending | Gaibu, Pernambuco, Brazil | 8S | 34W |
| Eriphia gonagra | pending | Gaibu, Pernambuco, Brazil | 8S | 34W |
| Eriphia gonagra | pending | Gaibu, Pernambuco, Brazil | 8S | 34W |
| Eriphia gonagra | pending | Gaibu, Pernambuco, Brazil | 8S | 34W |
| Eriphia gonagra | pending | Gaibu, Pernambuco, Brazil | 8S | 34W |
| Eriphia gonagra | pending | Gaibu, Pernambuco, Brazil | 8S | 34W |
| Eriphia gonagra | pending | Gaibu, Pernambuco, Brazil | 8S | 34W |
| Eriphia gonagra | pending | Gaibu, Pernambuco, Brazil | 8S | 34W |
| Eriphia gonagra | pending | Gaibu, Pernambuco, Brazil | 8S | 34W |
| Eriphia gonagra | pending | Gaibu, Pernambuco, Brazil | 8S | 34W |
| Eriphia gonagra | pending | Gaibu, Pernambuco, Brazil | 8S | 34W |
| Eriphia gonagra | pending | Gaibu, Pernambuco, Brazil | 8S | 34W |
| Eriphia gonagra | pending | Ipojuca, Pernambuco, Brazil | 8S | 34W |
| Goniopsis cruentata | KU313286 | Tobago, Trinidad and Tobago | 10N | 61W |
| Goniopsis cruentata | KU313284 | Trinidad, Trinidad and Tobago | 10N | 61W |
| Goniopsis cruentata | KU313285 | Trinidad, Trinidad and Tobago | 10N | 61W |
| Goniopsis cruentata | MK783695 | Água Dulce, Veracruz, Mexico | 18.5N | 94.5W |
| Goniopsis cruentata | KU313268 | Água Dulce, Veracruz, Mexico | 18.5N | 94.5W |
| Goniopsis cruentata | MK783692 | Catemaco, Veracruz, Mexico | 18.6N | 95W |
| Goniopsis cruentata | KU313265 | Catemaco, Veracruz, Mexico | 18.6N | 95W |
| Goniopsis cruentata | MK783693 | San Andrés Tuxtla, Veracruz, Mexico | 18.6N | 95.1W |
| Goniopsis cruentata | KU313266 | San Andrés Tuxtla, Veracruz, Mexico | 18.6N | 95.1W |
| Goniopsis cruentata | KU313292 | Calçoene, Amapá, Brazil | 2.5N | 50.5W |
| Goniopsis cruentata | KU313291 | Calçoene, Amapá, Brazil | 2.5N | 50.5W |
| Goniopsis cruentata | KU313293 | Calçoene, Amapá, Brazil | 2.5N | 50.5W |
| Goniopsis cruentata | KY964583 | Guantamano Bay, Cuba | 20N | 75W |
| Goniopsis cruentata | KY964584 | Guantamano Bay, Cuba | 20N | 75W |
| Goniopsis cruentata | KY964582 | Port Moa, Cuba | 20N | 74W |
| Goniopsis cruentata | KU313290 | Barcadaire du Mahury, French Guiana | 4.5N | 52W |
| Goniopsis cruentata | KU313287 | Plage de Rainuré, French Guiana | 4.7N | 52W |
| Goniopsis cruentata | KU313289 | Plage de Rainuré, French Guiana | 4.7N | 52W |
| Goniopsis cruentata | KU313288 | Plage de Rainuré, French Guiana | 4.7N | 52W |
| Goniopsis cruentata | KU313276 | Lagunda de Gandoca, Costa Rica | 9N | 82W |
| Goniopsis cruentata | KU313277 | Lagunda de Gandoca, Costa Rica | 9N | 82W |
| Goniopsis cruentata | KU313274 | Lagunda de Gandoca, Costa Rica | 9N | 82W |
| Goniopsis cruentata | KU313278 | Lagunda de Gandoca, Costa Rica | 9N | 82W |
| Goniopsis cruentata | KU313280 | Lagunda de Gandoca, Costa Rica | 9N | 82W |
| Goniopsis cruentata | MN183971 | Lagunda de Gandoca, Costa Rica | 9N | 82W |
| Goniopsis cruentata | KU313279 | Lagunda de Gandoca, Costa Rica | 9N | 82W |
| Goniopsis cruentata | KU313275 | Lagunda de Gandoca, Costa Rica | 9N | 82W |
| Goniopsis cruentata | KU313294 | Bragança, Pará, Brazil | 0 | 46W |
| Goniopsis cruentata | KU313296 | Bragança, Pará, Brazil | 0 | 46W |
| Goniopsis cruentata | KU313295 | Bragança, Pará, Brazil | 0 | 46W |
| Goniopsis cruentata | KU313308 | Porto Seguro, Bahia, Brazil | 16S | 39W |
| Goniopsis cruentata | KU313310 | Porto Seguro, Bahia, Brazil | 16S | 39W |
| Goniopsis cruentata | KU313309 | Porto Seguro, Bahia, Brazil | 16S | 39W |
| Goniopsis cruentata | KU313315 | Guarapari, Espíirito Santo, Brazil | 20S | 40W |
| Goniopsis cruentata | KU313316 | Guarapari, Espírito Santo, Brazil | 20S | 40W |
| Goniopsis cruentata | KU313314 | Guarapari, Espírito Santo, Brazil | 20S | 40W |
| Goniopsis cruentata | KU313317 | Guarapari, Espírito Santo, Brazil | 20S | 40W |
| Goniopsis cruentata | KU313329 | Bertioga, São Paulo, Brazil | 23.5S | 45W |
| Goniopsis cruentata | KU313325 | Ubatuba, São Paulo, Brazil | 23S | 44W |
| Goniopsis cruentata | KU313322 | Ubatuba, São Paulo, Brazil | 23S | 44W |
| Goniopsis cruentata | KU313319 | Ubatuba, São Paulo, Brazil | 23S | 44W |
| Goniopsis cruentata | KU313327 | Ubatuba, São Paulo, Brazil | 23S | 44W |
| Goniopsis cruentata | KU313328 | Ubatuba, São Paulo, Brazil | 23S | 44W |
| Goniopsis cruentata | KU313318 | Ubatuba, São Paulo, Brazil | 23S | 44W |
| Goniopsis cruentata | KU313324 | Ubatuba, São Paulo, Brazil | 23S | 44W |
| Goniopsis cruentata | KU313320 | Ubatuba, São Paulo, Brazil | 23S | 44W |
| Goniopsis cruentata | KU313323 | Ubatuba, São Paulo, Brazil | 23S | 44W |
| Goniopsis cruentata | KU313326 | Ubatuba, São Paulo, Brazil | 23S | 44W |
| Goniopsis cruentata | KU313333 | Ilha comprida, São Paulo, Brazil | 25S | 48W |
| Goniopsis cruentata | KU313334 | Ilha comprida, São Paulo, Brazil | 25S | 48W |
| Goniopsis cruentata | KU313332 | Ilha comprida, São Paulo, Brazil | 25S | 48W |
| Goniopsis cruentata | KU313331 | Ilha comprida, São Paulo, Brazil | 25S | 48W |
| Goniopsis cruentata | KU313330 | Ilha comprida, São Paulo, Brazil | 25S | 48W |
| Goniopsis cruentata | KU313300 | Natal, Rio Grande do Norte, Brazil | 5S | 35W |
| Goniopsis cruentata | KU313299 | Natal, Rio Grande do Norte, Brazil | 5S | 35W |
| Goniopsis cruentata | KU313301 | Natal, Rio Grande do Norte, Brazil | 5S | 35W |
| Goniopsis cruentata | KU313302 | Tamandaré, Pernambuco, Brazil | 8.5S | 35W |
| Goniopsis cruentata | KU313304 | Tamandaré, Pernambuco, Brazil | 8.5S | 35W |
| Goniopsis cruentata | KU313303 | Tamandaré, Pernambuco, Brazil | 8.5S | 35W |
| Goniopsis cruentata | KU313307 | Passo do Camaragibe, Alagoas, Brazil | 9S | 35W |
| Goniopsis cruentata | KU313305 | Passo do Camaragibe, Alagoas, Brazil | 9S | 35W |
| Goniopsis cruentata | KU313306 | Passo do Camaragibe, Alagoas, Brazil | 9S | 35W |
| Leptuca leptodactyla | LN610512 | Venezuela | 10.6N | 64W |
| Leptuca leptodactyla | LN610512 | Venezuela | 10.6N | 64W |
| Leptuca leptodactyla | LN610512 | Venezuela | 10.6N | 64W |
| Leptuca leptodactyla | LN610512 | Venezuela | 10.6N | 64W |
| Leptuca leptodactyla | LN610512 | Venezuela | 10.6N | 64W |
| Leptuca leptodactyla | LN610512 | Venezuela | 10.6N | 64W |
| Leptuca leptodactyla | LN610512 | Venezuela | 10.6N | 64W |
| Leptuca leptodactyla | LN610512 | Venezuela | 10.6N | 64W |
| Leptuca leptodactyla | LN610512 | Venezuela | 10.6N | 64W |
| Leptuca leptodactyla | LN610517 | Venezuela | 10.6N | 64W |
| Leptuca leptodactyla | LN610518 | Venezuela | 10.6N | 64W |
| Leptuca leptodactyla | LN610519 | Venezuela | 10.6N | 64W |
| Leptuca leptodactyla | LN610512 | Curaçao | 12N | 68W |
| Leptuca leptodactyla | LN610512 | Curaçao | 12N | 68W |
| Leptuca leptodactyla | LN610512 | Curaçao | 12N | 68W |
| Leptuca leptodactyla | LN610512 | Curaçao | 12N | 68W |
| Leptuca leptodactyla | LN610512 | Curaçao | 12N | 68W |
| Leptuca leptodactyla | LN610512 | Curaçao | 12N | 68W |
| Leptuca leptodactyla | LN610512 | Curaçao | 12N | 68W |
| Leptuca leptodactyla | LN610512 | Curaçao | 12N | 68W |
| Leptuca leptodactyla | LN610512 | Curaçao | 12N | 68W |
| Leptuca leptodactyla | LN610512 | Curaçao | 12N | 68W |
| Leptuca leptodactyla | LN610512 | Curaçao | 12N | 68W |
| Leptuca leptodactyla | LN610512 | Curaçao | 12N | 68W |
| Leptuca leptodactyla | LN610512 | Curaçao | 12N | 68W |
| Leptuca leptodactyla | LN610512 | Curaçao | 12N | 68W |
| Leptuca leptodactyla | LN610512 | Jamaica | 17N | 76W |
| Leptuca leptodactyla | LN610512 | Jamaica | 17N | 76W |
| Leptuca leptodactyla | LN610512 | Jamaica | 17N | 76W |
| Leptuca leptodactyla | LN610512 | Jamaica | 17N | 76W |
| Leptuca leptodactyla | LN610512 | Jamaica | 17N | 76W |
| Leptuca leptodactyla | LN610512 | Jamaica | 17N | 76W |
| Leptuca leptodactyla | LN610512 | Jamaica | 17N | 76W |
| Leptuca leptodactyla | LN610512 | Jamaica | 17N | 76W |
| Leptuca leptodactyla | LN610512 | Jamaica | 17N | 76W |
| Leptuca leptodactyla | LN610512 | Jamaica | 17N | 76W |
| Leptuca leptodactyla | LN610512 | Jamaica | 17N | 76W |
| Leptuca leptodactyla | LN610512 | Jamaica | 17N | 76W |
| Leptuca leptodactyla | LN610512 | Jamaica | 17N | 76W |
| Leptuca leptodactyla | LN610512 | Jamaica | 17N | 76W |
| Leptuca leptodactyla | LN610512 | Jamaica | 17N | 76W |
| Leptuca leptodactyla | LN610512 | Jamaica | 17N | 76W |
| Leptuca leptodactyla | LN610512 | Jamaica | 17N | 76W |
| Leptuca leptodactyla | LN610512 | Jamaica | 17N | 76W |
| Leptuca leptodactyla | LN610512 | Jamaica | 17N | 76W |
| Leptuca leptodactyla | LN610513 | Jamaica | 17N | 76W |
| Leptuca leptodactyla | LN610516 | Saint Martin | 18N | 63W |
| Leptuca leptodactyla | LN610512 | Saint Martin | 18N | 63W |
| Leptuca leptodactyla | LN610512 | Saint Martin | 18N | 63W |
| Leptuca leptodactyla | LN610512 | Saint Martin | 18N | 63W |
| Leptuca leptodactyla | LN610512 | Saint Martin | 18N | 63W |
| Leptuca leptodactyla | LN610512 | Dominican Republic | 19.7N | 70.4W |
| Leptuca leptodactyla | LN610512 | Dominican Republic | 19.7N | 70.4W |
| Leptuca leptodactyla | LN610512 | Dominican Republic | 19.7N | 70.4W |
| Leptuca leptodactyla | LN610512 | Dominican Republic | 19.7N | 70.4W |
| Leptuca leptodactyla | LN610512 | Dominican Republic | 19.7N | 70.4W |
| Leptuca leptodactyla | LN610514 | Dominican Republic | 19.7N | 70.4W |
| Leptuca leptodactyla | LN610515 | Dominican Republic | 19.7N | 70.4W |
| Leptuca leptodactyla | LN610520 | Pará, Brazil | 1S | 48.5W |
| Leptuca leptodactyla | LN610520 | Pará, Brazil | 1S | 48.5W |
| Leptuca leptodactyla | LN610520 | Pará, Brazil | 1S | 48.5W |
| Leptuca leptodactyla | LN610527 | Pará, Brazil | 1S | 48.5W |
| Leptuca leptodactyla | LN610526 | Pará, Brazil | 1S | 48.5W |
| Leptuca leptodactyla | LN610525 | Pará, Brazil | 1S | 48.5W |
| Leptuca leptodactyla | LN610528 | Pará, Brazil | 1S | 48.5W |
| Leptuca leptodactyla | LN610523 | Pará, Brazil | 1S | 48.5W |
| Leptuca leptodactyla | LN610522 | Pará, Brazil | 1S | 48.5W |
| Leptuca leptodactyla | LN610524 | Pará, Brazil | 1S | 48.5W |
| Leptuca leptodactyla | LN610521 | Pará, Brazil | 1S | 48.5W |
| Leptuca leptodactyla | LN610520 | Bahia, Brazil | 13S | 38W |
| Leptuca leptodactyla | LN610520 | Bahia, Brazil | 13S | 38W |
| Leptuca leptodactyla | LN610520 | Bahia, Brazil | 13S | 38W |
| Leptuca leptodactyla | LN610520 | Bahia, Brazil | 13S | 38W |
| Leptuca leptodactyla | LN610527 | Bahia, Brazil | 13S | 38W |
| Leptuca leptodactyla | LN610527 | Bahia, Brazil | 13S | 38W |
| Leptuca leptodactyla | LN610527 | Bahia, Brazil | 13S | 38W |
| Leptuca leptodactyla | LN610534 | Bahia, Brazil | 13S | 38W |
| Leptuca leptodactyla | LN610531 | Bahia, Brazil | 13S | 38W |
| Leptuca leptodactyla | LN610529 | Bahia, Brazil | 13S | 38W |
| Leptuca leptodactyla | LN610533 | Bahia, Brazil | 13S | 38W |
| Leptuca leptodactyla | LN610532 | Bahia, Brazil | 13S | 38W |
| Leptuca leptodactyla | LN610530 | Bahia, Brazil | 13S | 38W |
| Leptuca leptodactyla | LN610535 | São Paulo, Brazil | 23S | 44W |
| Leptuca leptodactyla | LN610537 | São Paulo, Brazil | 23S | 44W |
| Leptuca leptodactyla | LN610538 | São Paulo, Brazil | 23S | 44W |
| Leptuca leptodactyla | LN610536 | São Paulo, Brazil | 23S | 44W |
| Leptuca thayeri | MK783690 | Port Henderson, Jamaica | 17N | 76W |
| Leptuca thayeri | KU313390 | Port Henderson, Jamaica | 17N | 76W |
| Leptuca thayeri | KU313391 | Port Henderson, Jamaica | 17N | 76W |
| Leptuca thayeri | KU313388 | Campeche, Campeche, Mexico | 19.5N | 91W |
| Leptuca thayeri | KU313389 | Campeche, Campeche, Mexico | 19.5N | 91W |
| Leptuca thayeri | MK783691 | Champotón, Campeche, Mexico | 19.5N | 91W |
| Leptuca thayeri | KU313382 | Fort Pierce, Florida, USA | 27N | 80W |
| Leptuca thayeri | KU313381 | Fort Pierce, Florida, USA | 27N | 80W |
| Leptuca thayeri | KU313380 | Fort Pierce, Florida, USA | 27N | 80W |
| Leptuca thayeri | KU313383 | Fort Pierce, Florida, USA | 27N | 80W |
| Leptuca thayeri | KU313384 | Fort Pierce, Florida, USA | 27N | 80W |
| Leptuca thayeri | LC087969 | Hutchinson Island, Florida, USA | 27N | 80W |
| Leptuca thayeri | KU313447 | Florianópolis, Santa Catarina, Brazil | 27S | 48W |
| Leptuca thayeri | KU313448 | Florianópolis, Santa Catarina, Brazil | 27S | 48W |
| Leptuca thayeri | KU313446 | Florianópolis, Santa Catarina, Brazil | 27S | 48W |
| Leptuca thayeri | KU313445 | Florianópolis, Santa Catarina, Brazil | 27S | 48W |
| Leptuca thayeri | KU313394 | Salinópolis, Pará, Brazil | 0 | 47W |
| Leptuca thayeri | KU313395 | Salinópolis, Pará, Brazil | 0 | 47W |
| Leptuca thayeri | KU313396 | Salinópolis, Pará, Brazil | 0 | 47W |
| Leptuca thayeri | KU313397 | Salinópolis, Pará, Brazil | 0 | 47W |
| Leptuca thayeri | KU313398 | Salinópolis, Pará, Brazil | 0 | 47W |
| Leptuca thayeri | KU313419 | Porto Seguro, Bahia, Brazil | 16S | 39W |
| Leptuca thayeri | KU313420 | Porto Seguro, Bahia, Brazil | 16S | 39W |
| Leptuca thayeri | KU313421 | Porto Seguro, Bahia, Brazil | 16S | 39W |
| Leptuca thayeri | KU313427 | Baia do Sepitiba, Rio de Janeiro, Brazil | 22.9S | 43.7W |
| Leptuca thayeri | KU313426 | Baia do Sepitiba, Rio de Janeiro, Brazil | 22.9S | 43.7W |
| Leptuca thayeri | KU313425 | Baia do Sepitiba, Rio de Janeiro, Brazil | 22.9S | 43.7W |
| Leptuca thayeri | KU313429 | Paraty, Rio de Janeiro, Brazil | 23S | 44W |
| Leptuca thayeri | KU313431 | Ubatuba, São Paulo, Brazil | 23S | 44W |
| Leptuca thayeri | KU313435 | Ubatuba, São Paulo, Brazil | 23S | 44W |
| Leptuca thayeri | KU313434 | Ubatuba, São Paulo, Brazil | 23S | 44W |
| Leptuca thayeri | KU313433 | Ubatuba, São Paulo, Brazil | 23S | 44W |
| Leptuca thayeri | KU313432 | Ubatuba, São Paulo, Brazil | 23S | 44W |
| Leptuca thayeri | KU313428 | Ubatuba, São Paulo, Brazil | 23S | 44W |
| Leptuca thayeri | KU313430 | Ubatuba, São Paulo, Brazil | 23S | 44W |
| Leptuca thayeri | KU313440 | Cananéia, São Paulo, Brazil | 25S | 47W |
| Leptuca thayeri | KU313439 | Cananéia, São Paulo, Brazil | 25S | 47W |
| Leptuca thayeri | KU313444 | Guaratuba, Paraná, Brazil | 25S | 48W |
| Leptuca thayeri | KU313442 | Guaratuba, Paraná, Brazil | 25S | 48W |
| Leptuca thayeri | KU313441 | Guaratuba, Paraná, Brazil | 25S | 48W |
| Leptuca thayeri | KU313443 | Guaratuba, Paraná, Brazil | 25S | 48W |
| Leptuca thayeri | KU313438 | Guaratuba, Paraná, Brazil | 25S | 48W |
| Leptuca thayeri | KU313437 | Guaratuba, Paraná, Brazil | 25S | 48W |
| Leptuca thayeri | KU313436 | Guaratuba, Paraná, Brazil | 25S | 48W |
| Leptuca thayeri | KU313402 | Itacu, Maranhão, Brazil | 2S | 44W |
| Leptuca thayeri | KU313400 | Itacu, Maranhão, Brazil | 2S | 44W |
| Leptuca thayeri | KU313401 | Itacu, Maranhão, Brazil | 2S | 44W |
| Leptuca thayeri | KU313399 | Itacu, Maranhão, Brazil | 2S | 44W |
| Leptuca thayeri | KU313404 | Fortaleza, Ceará, Brazil | 3S | 38W |
| Leptuca thayeri | KU313403 | Fortaleza, Ceará, Brazil | 3S | 38W |
| Leptuca thayeri | KU313406 | Fortaleza, Ceará, Brazil | 3S | 38W |
| Leptuca thayeri | KU313405 | Fortaleza, Ceará, Brazil | 3S | 38W |
| Leptuca thayeri | KU313409 | Sirinhaem, Pernambuco, Brazil | 8.5S | 35W |
| Leptuca thayeri | KU313408 | Sirinhaem, Pernambuco, Brazil | 8.5S | 35W |
| Leptuca thayeri | KU313407 | Sirinhaem, Pernambuco, Brazil | 8.5S | 35W |
| Leptuca thayeri | KU313413 | Ilha de Tamaracá, Pernambuco, Brazil | 8S | 34W |
| Leptuca thayeri | KU313411 | Ilha de Tamaracá, Pernambuco, Brazil | 8S | 34W |
| Leptuca thayeri | KU313412 | Ilha de Tamaracá, Pernambuco, Brazil | 8S | 34W |
| Leptuca thayeri | KU313415 | Passo do Camaragibe, Alagoas, Brazil | 9S | 35W |
| Leptuca thayeri | KU313416 | Passo do Camaragibe, Alagoas, Brazil | 9S | 35W |
| Leptuca thayeri | KU313414 | Passo do Camaragibe, Alagoas, Brazil | 9S | 35W |
| Minuca rapax | LC388611 | Belize | 16N | 88W |
| Minuca rapax | LC388612 | Belize | 16N | 88W |
| Minuca rapax | LC388618 | Guadaloupe | 16N | 61W |
| Minuca rapax | LC388614 | Honduras | 16N | 86W |
| Minuca rapax | LC388613 | Honduras | 16N | 86W |
| Minuca rapax | LC087956 | Jamaica | 17N | 76W |
| Minuca rapax | LC087956 | Jamaica | 17N | 76W |
| Minuca rapax | LC087956 | Jamaica | 17N | 76W |
| Minuca rapax | LC388615 | Jamaica | 17N | 76W |
| Minuca rapax | LC087956 | Puerto Rico | 17N | 66W |
| Minuca rapax | LC087956 | Puerto Rico | 17N | 66W |
| Minuca rapax | LC388616 | Puerto Rico | 17N | 66W |
| Minuca rapax | LC087956 | British Virgin Islands | 18N | 64W |
| Minuca rapax | LC388617 | British Virgin Islands | 18N | 64W |
| Minuca rapax | LC087956 | U.S. Virgin Islands | 18N | 64W |
| Minuca rapax | LC388617 | U.S. Virgin Islands | 18N | 64W |
| Minuca rapax | LC087956 | Florida | 28N | 82.6W |
| Minuca rapax | LC087956 | Florida | 28N | 82.6W |
| Minuca rapax | LC388604 | Florida | 28N | 82.6W |
| Minuca rapax | LC388607 | Florida | 28N | 82.6W |
| Minuca rapax | LC388608 | Florida | 28N | 82.6W |
| Minuca rapax | LC087956 | Amapá, Brazil | 2N | 50W |
| Minuca rapax | LC087956 | Amapá, Brazil | 2N | 50W |
| Minuca rapax | LC388619 | Amapá, Brazil | 2N | 50W |
| Minuca rapax | LC087956 | Bahia, Brazil | 12S | 37W |
| Minuca rapax | LC388619 | Bahia, Brazil | 12S | 37W |
| Minuca rapax | LC388626 | Bahia, Brazil | 12S | 37W |
| Minuca rapax | LC388627 | Espírito Santo, Brazil | 19S | 39.7W |
| Minuca rapax | LC388619 | Espírito Santo, Brazil | 19S | 39.7W |
| Minuca rapax | LC388619 | Espírito Santo, Brazil | 19S | 39.7W |
| Minuca rapax | LC388619 | Espírito Santo, Brazil | 19S | 39.7W |
| Minuca rapax | LC388619 | Rio de Janeiro, Brazil | 22.6S | 41.8W |
| Minuca rapax | LC388619 | Rio de Janeiro, Brazil | 22.6S | 41.8W |
| Minuca rapax | LC388619 | Rio de Janeiro, Brazil | 22.6S | 41.8W |
| Minuca rapax | LC388619 | Rio de Janeiro, Brazil | 22.6S | 41.8W |
| Minuca rapax | LC388619 | Rio de Janeiro, Brazil | 22.6S | 41.8W |
| Minuca rapax | LC388619 | Rio de Janeiro, Brazil | 22.6S | 41.8W |
| Minuca rapax | LC388619 | Rio de Janeiro, Brazil | 22.6S | 41.8W |
| Minuca rapax | LC388629 | Rio de Janeiro, Brazil | 22.6S | 41.8W |
| Minuca rapax | LC388619 | Rio de Janeiro, Brazil | 22.6S | 41.8W |
| Minuca rapax | LC388619 | Rio de Janeiro, Brazil | 22.6S | 41.8W |
| Minuca rapax | LC388619 | Rio de Janeiro, Brazil | 22.6S | 41.8W |
| Minuca rapax | LC388619 | Rio de Janeiro, Brazil | 22.6S | 41.8W |
| Minuca rapax | LC388619 | Rio de Janeiro, Brazil | 22.6S | 41.8W |
| Minuca rapax | LC388619 | Rio de Janeiro, Brazil | 22.6S | 41.8W |
| Minuca rapax | LC388619 | Rio de Janeiro, Brazil | 22.6S | 41.8W |
| Minuca rapax | LC388619 | Paraná, Brazil | 25S | 48W |
| Minuca rapax | LC388619 | Paraná, Brazil | 25S | 48W |
| Minuca rapax | LC388619 | Paraná, Brazil | 25S | 48W |
| Minuca rapax | LC388619 | Ceará, Brazil | 2S | 38W |
| Minuca rapax | LC388619 | Ceará, Brazil | 2S | 38W |
| Minuca rapax | LC087956 | Maranhão, Brazil | 2S | 44W |
| Minuca rapax | LC388620 | Maranhão, Brazil | 2S | 44W |
| Minuca rapax | LC388621 | Maranhão, Brazil | 2S | 44W |
| Minuca rapax | LC087956 | Ceará, Brazil | 3S | 38W |
| Minuca rapax | LC388623 | Ceará, Brazil | 3S | 38W |
| Minuca rapax | LC388624 | Ceará, Brazil | 3S | 38W |
| Sesarma rectum | KY964587 | Trinidad, Trinidad and Tobago | 10N | 61W |
| Sesarma rectum | KU313339 | Trinidad, Trinidad and Tobago | 10N | 61W |
| Sesarma rectum | KU313336 | Trinidad, Trinidad and Tobago | 10N | 61W |
| Sesarma rectum | KU313337 | Trinidad, Trinidad and Tobago | 10N | 61W |
| Sesarma rectum | KU313340 | Trinidad, Trinidad and Tobago | 10N | 61W |
| Sesarma rectum | KU313338 | Trinidad, Trinidad and Tobago | 10N | 61W |
| Sesarma rectum | KU313341 | Bragança, Pará, Brazil | 0 | 46W |
| Sesarma rectum | KU313342 | Bragança, Pará, Brazil | 0 | 46W |
| Sesarma rectum | KU313344 | Bragança, Pará, Brazil | 0 | 46W |
| Sesarma rectum | KU313345 | Bragança, Pará, Brazil | 0 | 46W |
| Sesarma rectum | KU313343 | Bragança, Pará, Brazil | 0 | 46W |
| Sesarma rectum | KU313358 | Porto Seguro, Bahia, Brazil | 16S | 39W |
| Sesarma rectum | KU313359 | Porto Seguro, Bahia, Brazil | 16S | 39W |
| Sesarma rectum | KU313360 | Porto Seguro, Bahia, Brazil | 16S | 39W |
| Sesarma rectum | KU313362 | Porto Seguro, Bahia, Brazil | 16S | 39W |
| Sesarma rectum | KU313361 | Porto Seguro, Bahia, Brazil | 16S | 39W |
| Sesarma rectum | KU313371 | Bertioga, São Paulo, Brazil | 23.5S | 45W |
| Sesarma rectum | KU313372 | Bertioga, São Paulo, Brazil | 23.5S | 45W |
| Sesarma rectum | KU313373 | Bertioga, São Paulo, Brazil | 23.5S | 45W |
| Sesarma rectum | KU313374 | Bertioga, São Paulo, Brazil | 23.5S | 45W |
| Sesarma rectum | KU313368 | Ubatuba, São Paulo, Brazil | 23S | 44W |
| Sesarma rectum | KU313369 | Ubatuba, São Paulo, Brazil | 23S | 44W |
| Sesarma rectum | KU313366 | Ubatuba, São Paulo, Brazil | 23S | 44W |
| Sesarma rectum | KU313370 | Ubatuba, São Paulo, Brazil | 23S | 44W |
| Sesarma rectum | KU313367 | Ubatuba, São Paulo, Brazil | 23S | 44W |
| Sesarma rectum | KU313375 | Ilha comprida, São Paulo, Brazil | 24.7S | 47.5W |
| Sesarma rectum | KU313378 | Ilha comprida, São Paulo, Brazil | 24.7S | 47.5W |
| Sesarma rectum | KU313376 | Ilha comprida, São Paulo, Brazil | 24.7S | 47.5W |
| Sesarma rectum | KU313377 | Ilha comprida, São Paulo, Brazil | 24.7S | 47.5W |
| Sesarma rectum | KU313379 | Ilha comprida, São Paulo, Brazil | 24.7S | 47.5W |
| Sesarma rectum | KU313347 | Fortaleza, Ceará, Brazil | 3S | 38W |
| Sesarma rectum | KU313349 | Fortaleza, Ceará, Brazil | 3S | 38W |
| Sesarma rectum | KU313348 | Fortaleza, Ceará, Brazil | 3S | 38W |
| Sesarma rectum | KU313346 | Fortaleza, Ceará, Brazil | 3S | 38W |
| Sesarma rectum | KU313351 | Natal, Rio Grande do Norte, Brazil | 5S | 35W |
| Sesarma rectum | KU313353 | Natal, Rio Grande do Norte, Brazil | 5S | 35W |
| Sesarma rectum | KU313354 | Natal, Rio Grande do Norte, Brazil | 5S | 35W |
| Sesarma rectum | KU313352 | Natal, Rio Grande do Norte, Brazil | 5S | 35W |
| Sesarma rectum | KU313350 | Natal, Rio Grande do Norte, Brazil | 5S | 35W |
| Sesarma rectum | KU313355 | Passo do Camaragibe, Alagoas, Brazil | 9S | 35W |
| Sesarma rectum | KU313356 | Passo do Camaragibe, Alagoas, Brazil | 9S | 35W |
| Sesarma rectum | KU313357 | Passo do Camaragibe, Alagoas, Brazil | 9S | 35W |
| Ucides cordatus | KU313456 | Trinidad, Trinidad and Tobago | 10N | 61W |
| Ucides cordatus | KU313455 | Trinidad, Trinidad and Tobago | 10N | 61W |
| Ucides cordatus | KU313453 | Trinidad, Trinidad and Tobago | 10N | 61W |
| Ucides cordatus | KU313454 | Trinidad, Trinidad and Tobago | 10N | 61W |
| Ucides cordatus | KU313461 | Veracruz, México | 18N | 95W |
| Ucides cordatus | KU313458 | Veracruz, México | 18N | 95W |
| Ucides cordatus | KU313459 | Veracruz, México | 18N | 95W |
| Ucides cordatus | KU313460 | Veracruz, México | 18N | 95W |
| Ucides cordatus | KU313457 | Veracruz, México | 18N | 95W |
| Ucides cordatus | KU313464 | Calçoene, Amapá, Brazil | 2.5N | 50.5W |
| Ucides cordatus | KU313463 | Calçoene, Amapá, Brazil | 2.5N | 50.5W |
| Ucides cordatus | KU313462 | Calçoene, Amapá, Brazil | 2.5N | 50.5W |
| Ucides cordatus | KU313465 | Calçoene, Amapá, Brazil | 2.5N | 50.5W |
| Ucides cordatus | KY964592 | Havana, Cuba | 23.5N | 82W |
| Ucides cordatus | KY964591 | Havana, Cuba | 23.5N | 82W |
| Ucides cordatus | KY964593 | Havana, Cuba | 23.5N | 82W |
| Ucides cordatus | KU313470 | Bragança, Pará, Brazil | 0 | 46W |
| Ucides cordatus | KU313469 | Bragança, Pará, Brazil | 0 | 46W |
| Ucides cordatus | KU313468 | Bragança, Pará, Brazil | 0 | 46W |
| Ucides cordatus | KU313486 | Ilhéus, Bahia, Brazil | 14S | 39W |
| Ucides cordatus | KU313487 | Ilhéus, Bahia, Brazil | 14S | 39W |
| Ucides cordatus | KU313485 | Ilhéus, Bahia, Brazil | 14S | 39W |
| Ucides cordatus | KU313493 | Duque de Caxias, Rio de Janeiro, Brazil | 22.7S | 43.2W |
| Ucides cordatus | KU313494 | Duque de Caxias, Rio de Janeiro, Brazil | 22.7S | 43.2W |
| Ucides cordatus | KU313492 | Duque de Caxias, Rio de Janeiro, Brazil | 22.7S | 43.2W |
| Ucides cordatus | KU313495 | Duque de Caxias, Rio de Janeiro, Brazil | 22.7S | 43.2W |
| Ucides cordatus | KU313491 | Niterói, Rio de Janeiro, Brazil | 22.7S | 43.2W |
| Ucides cordatus | KU313504 | Bertioga, São Paulo, Brazil | 23.5S | 45W |
| Ucides cordatus | KU313501 | Bertioga, São Paulo, Brazil | 23.5S | 45W |
| Ucides cordatus | KU313503 | Bertioga, São Paulo, Brazil | 23.5S | 45W |
| Ucides cordatus | KU313502 | Bertioga, São Paulo, Brazil | 23.5S | 45W |
| Ucides cordatus | KU313499 | Ubatuba, São Paulo, Brazil | 23S | 44W |
| Ucides cordatus | KU313496 | Ubatuba, São Paulo, Brazil | 23S | 44W |
| Ucides cordatus | KU313506 | Ubatuba, São Paulo, Brazil | 23S | 44W |
| Ucides cordatus | KU313498 | Ubatuba, São Paulo, Brazil | 23S | 44W |
| Ucides cordatus | KU313505 | Ubatuba, São Paulo, Brazil | 23S | 44W |
| Ucides cordatus | KU313507 | Ubatuba, São Paulo, Brazil | 23S | 44W |
| Ucides cordatus | KU313497 | Ubatuba, São Paulo, Brazil | 23S | 44W |
| Ucides cordatus | KU313500 | Ubatuba, São Paulo, Brazil | 23S | 44W |
| Ucides cordatus | KU313474 | Caucaia, Ceará, Brazil | 3S | 38W |
| Ucides cordatus | KU313472 | Caucaia, Ceará, Brazil | 3S | 38W |
| Ucides cordatus | KU313471 | Caucaia, Ceará, Brazil | 3S | 38W |
| Ucides cordatus | KU313473 | Fortaleza, Ceará, Brazil | 3S | 38W |
| Ucides cordatus | KU313477 | Natal, Rio Grande do Norte, Brazil | 5S | 35W |
| Ucides cordatus | KU313476 | Natal, Rio Grande do Norte, Brazil | 5S | 35W |
| Ucides cordatus | KU313478 | Natal, Rio Grande do Norte, Brazil | 5S | 35W |
| Ucides cordatus | KU313475 | Natal, Rio Grande do Norte, Brazil | 5S | 35W |
| Ucides cordatus | KU313481 | Ipojuca, Pernambuco, Brazil | 8S | 34W |
| Ucides cordatus | KU313480 | Ipojuca, Pernambuco, Brazil | 8S | 34W |
| Ucides cordatus | KU313479 | Ipojuca, Pernambuco, Brazil | 8S | 34W |
| Ucides cordatus | KU313482 | Ipojuca, Pernambuco, Brazil | 8S | 34W |
| Ucides cordatus | KU313483 | Ipojuca, Pernambuco, Brazil | 8S | 34W |
| Uca maracoani | OM938800 | Dominican Republic: Sanchez | 19N | 69W |
| Uca maracoani | OM938801 | Dominican Republic: Sanchez | 19N | 69W |
| Uca maracoani | OM938802 | Dominican Republic: Sanchez | 19N | 69W |
| Uca maracoani | OM938803 | Dominican Republic: Sanchez | 19N | 69W |
| Uca maracoani | OM938804 | Dominican Republic: Sanchez | 19N | 69W |
| Uca maracoani | OM938805 | Dominican Republic: Sanchez | 19N | 69W |
| Uca maracoani | OM938806 | Dominican Republic: Sanchez | 19N | 69W |
| Uca maracoani | OM938807 | Dominican Republic: Sanchez | 19N | 69W |
| Uca maracoani | OM938808 | Dominican Republic: Sanchez | 19N | 69W |
| Uca maracoani | OM938809 | Dominican Republic: Sanchez | 19N | 69W |
| Uca maracoani | OM938810 | Dominican Republic: Sanchez | 19N | 69W |
| Uca maracoani | KF666992 | Rio Amapá, Amapá, Brazil | 2N | 50W |
| Uca maracoani | KF666952 | Rio Amapá, Amapá, Brazil | 2N | 50W |
| Uca maracoani | KF666952 | Rio Amapá, Amapa, Brazil | 2N | 50W |
| Uca maracoani | KF666952 | Rio Amapa, Amapá, Brazil | 2N | 50W |
| Uca maracoani | KF666952 | Rio Amapá, Amapá, Brazil | 2N | 50W |
| Uca maracoani | KF666952 | Rio Amapá, Amapá, Brazil | 2N | 50W |
| Uca maracoani | KF666991 | Rio Amapá, Amapá, Brazil | 2N | 50W |
| Uca maracoani | KF666975 | Rio Amapá, Amapá, Brazil | 2N | 50W |
| Uca maracoani | KF666974 | Rio Amapá, Amapá, Brazil | 2N | 50W |
| Uca maracoani | KF666962 | Rio Amapá, Amapá, Brazil | 2N | 50W |
| Uca maracoani | KF666994 | Rio Amapá, Amapá, Brazil | 2N | 50W |
| Uca maracoani | KF666959 | Marudá, Pará, Brazil | 0 | 47W |
| Uca maracoani | KF666959 | Marudá, Pará, Brazil | 0 | 47W |
| Uca maracoani | KF666959 | Marudá, Pará, Brazil | 0 | 47W |
| Uca maracoani | KF666952 | Marudá, Pará, Brazil | 0 | 47W |
| Uca maracoani | KF666992 | Marudá, Pará, Brazil | 0 | 47W |
| Uca maracoani | KF666992 | Marudá, Pará, Brazil | 0 | 47W |
| Uca maracoani | KF666992 | Marudá, Pará, Brazil | 0 | 47W |
| Uca maracoani | KF666971 | Marudá, Pará, Brazil | 0 | 47W |
| Uca maracoani | KF666962 | Marudá, Pará, Brazil | 0 | 47W |
| Uca maracoani | KF666962 | Marudá, Pará, Brazil | 0 | 47W |
| Uca maracoani | KF666959 | São Jose do Ribamar, Maranhão, Brazil | 2S | 44W |
| Uca maracoani | KF666978 | São Jose do Ribamar, Maranhão, Brazil | 2S | 44W |
| Uca maracoani | KF666952 | São Jose do Ribamar, Maranhão, Brazil | 2S | 44W |
| Uca maracoani | KF666952 | São Jose do Ribamar, Maranhão, Brazil | 2S | 44W |
| Uca maracoani | KF666952 | São Jose do Ribamar, Maranhão, Brazil | 2S | 44W |
| Uca maracoani | KF666991 | São Jose do Ribamar, Maranhão, Brazil | 2S | 44W |
| Uca maracoani | KF666988 | São Jose do Ribamar, Maranhão, Brazil | 2S | 44W |
| Uca maracoani | KF666971 | São Jose do Ribamar, Maranhão, Brazil | 2S | 44W |
| Uca maracoani | KF666970 | São Jose do Ribamar, Maranhão, Brazil | 2S | 44W |
| Uca maracoani | KF666968 | São Jose do Ribamar, Maranhão, Brazil | 2S | 44W |
| Uca maracoani | KF666974 | São Jose do Ribamar, Maranhão, Brazil | 2S | 44W |
| Uca maracoani | KF666990 | São Jose do Ribamar, Maranhão, Brazil | 2S | 44W |
| Uca maracoani | KF666993 | Fortaleza, Ceará, Brazil | 3S | 38W |
| Uca maracoani | KF666980 | Fortaleza, Ceará, Brazil | 3S | 38W |
| Uca maracoani | KF666952 | Fortaleza, Ceará, Brazil | 3S | 38W |
| Uca maracoani | KF666952 | Fortaleza, Ceará, Brazil | 3S | 38W |
| Uca maracoani | KF666977 | Fortaleza, Ceará, Brazil | 3S | 38W |
| Uca maracoani | KF666973 | Fortaleza, Ceará, Brazil | 3S | 38W |
| Uca maracoani | KF666972 | Fortaleza, Ceará, Brazil | 3S | 38W |
| Uca maracoani | KF666969 | Fortaleza, Ceará, Brazil | 3S | 38W |
| Uca maracoani | KF666967 | Fortaleza, Ceará, Brazil | 3S | 38W |
| Uca maracoani | KF666979 | Fortaleza, Ceará, Brazil | 3S | 38W |
| Uca maracoani | KF666959 | Fortaleza, Ceará, Brazil | 3S | 38W |
| Uca maracoani | KF666959 | Fortaleza, Ceará, Brazil | 3S | 38W |
| Uca maracoani | KF666976 | Fortaleza, Ceará, Brazil | 3S | 38W |
| Uca maracoani | KF666953 | Itapissuma, Pernambuco, Brazil | 7S | 34W |
| Uca maracoani | KF666959 | Itapissuma, Pernambuco, Brazil | 7S | 34W |
| Uca maracoani | KF666952 | Itapissuma, Pernambuco, Brazil | 7S | 34W |
| Uca maracoani | KF666952 | Itapissuma, Pernambuco, Brazil | 7S | 34W |
| Uca maracoani | KF666952 | Itapissuma, Pernambuco, Brazil | 7S | 34W |
| Uca maracoani | KF666964 | Itapissuma, Pernambuco, Brazil | 7S | 34W |
| Uca maracoani | KF666986 | Itapissuma, Pernambuco, Brazil | 7S | 34W |
| Uca maracoani | KF666955 | Itapissuma, Pernambuco, Brazil | 7S | 34W |
| Uca maracoani | KF666994 | Itapissuma, Pernambuco, Brazil | 7S | 34W |
| Uca maracoani | KF666989 | Salvador, Bahia, Brazil | 12S | 38W |
| Uca maracoani | KF666952 | Salvador, Bahia, Brazil | 12S | 38W |
| Uca maracoani | KF666965 | Salvador, Bahia, Brazil | 12S | 38W |
| Uca maracoani | KF666957 | Salvador, Bahia, Brazil | 12S | 38W |
| Uca maracoani | KF666988 | Salvador, Bahia, Brazil | 12S | 38W |
| Uca maracoani | KF666962 | Salvador, Bahia, Brazil | 12S | 38W |
| Uca maracoani | KF666951 | Salvador, Bahia, Brazil | 12S | 38W |
| Uca maracoani | KF666984 | Salvador, Bahia, Brazil | 12S | 38W |
| Uca maracoani | KF666956 | Salvador, Bahia, Brazil | 12S | 38W |
| Uca maracoani | KF666995 | Santa Cruz, Espírito Santo, Brazil | 19.9S | 40.16W |
| Uca maracoani | KF666952 | Santa Cruz, Espírito Santo, Brazil | 19.9S | 40.16W |
| Uca maracoani | KF666952 | Santa Cruz, Espírito Santo, Brazil | 19.9S | 40.16W |
| Uca maracoani | KF666952 | Santa Cruz, Espírito Santo, Brazil | 19.9S | 40.16W |
| Uca maracoani | KF666961 | Santa Cruz, Espírito Santo, Brazil | 19.9S | 40.16W |
| Uca maracoani | KF666982 | Santa Cruz, Espírito Santo, Brazil | 19.9S | 40.16W |
| Uca maracoani | KF666959 | Santa Cruz, Espírito Santo, Brazil | 19.9S | 40.16W |
| Uca maracoani | KF666959 | Santa Cruz, Espírito Santo, Brazil | 19.9S | 40.16W |
| Uca maracoani | KF666958 | Santa Cruz, Espírito Santo, Brazil | 19.9S | 40.16W |
| Uca maracoani | KF666963 | Santa Cruz, Espírito Santo, Brazil | 19.9S | 40.16W |
| Uca maracoani | KF666981 | Paraty, Rio de Janeiro, Brazil | 23S | 44W |
| Uca maracoani | KF666952 | Paraty, Rio de Janeiro, Brazil | 23S | 44W |
| Uca maracoani | KF666952 | Paraty, Rio de Janeiro, Brazil | 23S | 44W |
| Uca maracoani | KF666952 | Paraty, Rio de Janeiro, Brazil | 23S | 44W |
| Uca maracoani | KF666952 | Paraty, Rio de Janeiro, Brazil | 23S | 44W |
| Uca maracoani | KF666952 | Paraty, Rio de Janeiro, Brazil | 23S | 44W |
| Uca maracoani | KF666952 | Paraty, Rio de Janeiro, Brazil | 23S | 44W |
| Uca maracoani | KF666952 | Paraty, Rio de Janeiro, Brazil | 23S | 44W |
| Uca maracoani | KF666952 | Paraty, Rio de Janeiro, Brazil | 23S | 44W |
| Uca maracoani | KF666971 | Paraty, Rio de Janeiro, Brazil | 23S | 44W |
| Uca maracoani | KF666983 | Guaratuba, Paraná, Brazil | 25S | 48W |
| Uca maracoani | KF666952 | Guaratuba, Paraná, Brazil | 25S | 48W |
| Uca maracoani | KF666952 | Guaratuba, Paraná, Brazil | 25S | 48W |
| Uca maracoani | KF666952 | Guaratuba, Paraná, Brazil | 25S | 48W |
| Uca maracoani | KF666960 | Guaratuba, Paraná, Brazil | 25S | 48W |
| Uca maracoani | KF666985 | Guaratuba, Paraná, Brazil | 25S | 48W |
| Uca maracoani | KF666954 | Guaratuba, Paraná, Brazil | 25S | 48W |
| Uca maracoani | KF666987 | Guaratuba, Paraná, Brazil | 25S | 48W |
| Minuca mordax | LC782439 | Calçoene, Amapá, Brazil | 2N | 50W |
| Minuca mordax | LC782440 | Calçoene, Amapá, Brazil | 2N | 50W |
| Minuca mordax | LC782441 | Calçoene, Amapá, Brazil | 2N | 50W |
| Minuca mordax | LC782442 | Calçoene, Amapá, Brazil | 2N | 50W |
| Minuca mordax | LC782443 | Calçoene, Amapá, Brazil | 2N | 50W |
| Minuca mordax | LC782444 | Calçoene, Amapá, Brazil | 2N | 50W |
| Minuca mordax | LC782445 | Calçoene, Amapá, Brazil | 2N | 50W |
| Minuca mordax | LC782446 | Calçoene, Amapá, Brazil | 2N | 50W |
| Minuca mordax | LC782447 | Calçoene, Amapá, Brazil | 2N | 50W |
| Minuca mordax | LC782448 | Calçoene, Amapá, Brazil | 2N | 50W |
| Minuca mordax | LC782449 | Calçoene, Amapá, Brazil | 2N | 50W |
| Minuca mordax | LC782450 | Belém, Pará, Brazil | 1S | 48W |
| Minuca mordax | LC782451 | Belém, Pará, Brazil | 1S | 48W |
| Minuca mordax | LC782452 | Belém, Pará, Brazil | 1S | 48W |
| Minuca mordax | LC782453 | Belém, Pará, Brazil | 1S | 48W |
| Minuca mordax | LC782454 | Belém, Pará, Brazil | 1S | 48W |
| Minuca mordax | LC782455 | Belém, Pará, Brazil | 1S | 48W |
| Minuca mordax | LC782456 | Belém, Pará, Brazil | 1S | 48W |
| Minuca mordax | LC782457 | Belém, Pará, Brazil | 1S | 48W |
| Minuca mordax | LC782458 | Belém, Pará, Brazil | 1S | 48W |
| Minuca mordax | LC782459 | Belém, Pará, Brazil | 1S | 48W |
| Minuca mordax | LC782359 | Rosario, Maranhão, Brazil | 2S | 44W |
| Minuca mordax | LC782360 | Rosario, Maranhão, Brazil | 2S | 44W |
| Minuca mordax | LC782361 | Rosario, Maranhão, Brazil | 2S | 44W |
| Minuca mordax | LC782362 | Rosario, Maranhão, Brazil | 2S | 44W |
| Minuca mordax | LC782363 | Rosario, Maranhão, Brazil | 2S | 44W |
| Minuca mordax | LC782364 | Rosario, Maranhão, Brazil | 2S | 44W |
| Minuca mordax | LC782365 | Rosario, Maranhão, Brazil | 2S | 44W |
| Minuca mordax | LC782366 | Rosario, Maranhão, Brazil | 2S | 44W |
| Minuca mordax | LC782367 | Rosario, Maranhão, Brazil | 2S | 44W |
| Minuca mordax | LC782368 | Rosario, Maranhão, Brazil | 2S | 44W |
| Minuca mordax | LC782369 | Rosario, Maranhão, Brazil | 2S | 44W |
| Minuca mordax | LC782347 | Natal, Rio Grande do Norte, Brazil | 5S | 35W |
| Minuca mordax | LC782348 | Natal, Rio Grande do Norte, Brazil | 5S | 35W |
| Minuca mordax | LC782349 | Natal, Rio Grande do Norte, Brazil | 5S | 35W |
| Minuca mordax | LC782350 | Natal, Rio Grande do Norte, Brazil | 5S | 35W |
| Minuca mordax | LC782351 | Natal, Rio Grande do Norte, Brazil | 5S | 35W |
| Minuca mordax | LC782352 | Natal, Rio Grande do Norte, Brazil | 5S | 35W |
| Minuca mordax | LC782353 | Natal, Rio Grande do Norte, Brazil | 5S | 35W |
| Minuca mordax | LC782354 | Natal, Rio Grande do Norte, Brazil | 5S | 35W |
| Minuca mordax | LC782355 | Natal, Rio Grande do Norte, Brazil | 5S | 35W |
| Minuca mordax | LC782356 | Natal, Rio Grande do Norte, Brazil | 5S | 35W |
| Minuca mordax | LC782357 | Natal, Rio Grande do Norte, Brazil | 5S | 35W |
| Minuca mordax | LC782358 | Natal, Rio Grande do Norte, Brazil | 5S | 35W |
| Minuca mordax | LC782370 | Ilhéus, Bahia, Brazil | 14S | 39W |
| Minuca mordax | LC782371 | Ilhéus, Bahia, Brazil | 14S | 39W |
| Minuca mordax | LC782372 | Ilhéus, Bahia, Brazil | 14S | 39W |
| Minuca mordax | LC782373 | Ilhéus, Bahia, Brazil | 14S | 39W |
| Minuca mordax | LC782374 | Ilhéus, Bahia, Brazil | 14S | 39W |
| Minuca mordax | LC782375 | Ilhéus, Bahia, Brazil | 14S | 39W |
| Minuca mordax | LC782376 | Ilhéus, Bahia, Brazil | 14S | 39W |
| Minuca mordax | LC782377 | Ilhéus, Bahia, Brazil | 14S | 39W |
| Minuca mordax | LC782378 | Ilhéus, Bahia, Brazil | 14S | 39W |
| Minuca mordax | LC782379 | Ilhéus, Bahia, Brazil | 14S | 39W |
| Minuca mordax | LC782380 | Ilhéus, Bahia, Brazil | 14S | 39W |
| Minuca mordax | LC782381 | Ilhéus, Bahia, Brazil | 14S | 39W |
| Minuca mordax | LC782382 | Vitória, Espírito Santo, Brazil | 20S | 40W |
| Minuca mordax | LC782383 | Vitória, Espírito Santo, Brazil | 20S | 40W |
| Minuca mordax | LC782384 | Vitória, Espírito Santo, Brazil | 20S | 40W |
| Minuca mordax | LC782385 | Vitória, Espírito Santo, Brazil | 20S | 40W |
| Minuca mordax | LC782386 | Vitória, Espírito Santo, Brazil | 20S | 40W |
| Minuca mordax | LC782387 | Vitória, Espírito Santo, Brazil | 20S | 40W |
| Minuca mordax | LC782388 | Vitória, Espírito Santo, Brazil | 20S | 40W |
| Minuca mordax | LC782389 | Vitória, Espírito Santo, Brazil | 20S | 40W |
| Minuca mordax | LC782390 | Vitória, Espírito Santo, Brazil | 20S | 40W |
| Minuca mordax | LC782391 | Vitória, Espírito Santo, Brazil | 20S | 40W |
| Minuca mordax | LC782392 | Vitória, Espírito Santo, Brazil | 20S | 40W |
| Minuca mordax | LC782393 | Macaé, Rio de Janeiro, Brazil | 22S | 41W |
| Minuca mordax | LC782394 | Macaé, Rio de Janeiro, Brazil | 22S | 41W |
| Minuca mordax | LC782395 | Macaé, Rio de Janeiro, Brazil | 22S | 41W |
| Minuca mordax | LC782396 | Macaé, Rio de Janeiro, Brazil | 22S | 41W |
| Minuca mordax | LC782397 | Macaé, Rio de Janeiro, Brazil | 22S | 41W |
| Minuca mordax | LC782398 | Macaé, Rio de Janeiro, Brazil | 22S | 41W |
| Minuca mordax | LC782399 | Macaé, Rio de Janeiro, Brazil | 22S | 41W |
| Minuca mordax | LC782400 | Macaé, Rio de Janeiro, Brazil | 22S | 41W |
| Minuca mordax | LC782401 | Macaé, Rio de Janeiro, Brazil | 22S | 41W |
| Minuca mordax | LC782402 | Macaé, Rio de Janeiro, Brazil | 22S | 41W |
| Minuca mordax | LC782403 | Macaé, Rio de Janeiro, Brazil | 22S | 41W |
| Minuca mordax | LC782404 | Guaratuba, Paraná, Brazil | 25S | 48W |
| Minuca mordax | LC782405 | Guaratuba, Paraná, Brazil | 25S | 48W |
| Minuca mordax | LC782406 | Guaratuba, Paraná, Brazil | 25S | 48W |
| Minuca mordax | LC782407 | Guaratuba, Paraná, Brazil | 25S | 48W |
| Minuca mordax | LC782408 | Guaratuba, Paraná, Brazil | 25S | 48W |
| Minuca mordax | LC782409 | Guaratuba, Paraná, Brazil | 25S | 48W |
| Minuca mordax | LC782410 | Guaratuba, Paraná, Brazil | 25S | 48W |
| Minuca mordax | LC782411 | Guaratuba, Paraná, Brazil | 25S | 48W |
| Minuca mordax | LC782412 | Guaratuba, Paraná, Brazil | 25S | 48W |
| Minuca mordax | LC782413 | Guaratuba, Paraná, Brazil | 25S | 48W |
| Minuca mordax | LC782414 | Guaratuba, Paraná, Brazil | 25S | 48W |
| Minuca mordax | LC782415 | Guaratuba, Paraná, Brazil | 25S | 48W |
| Minuca mordax | LC782416 | Palmital, Santa Catarina, Brazil | 26S | 48W |
| Minuca mordax | LC782417 | Palmital, Santa Catarina, Brazil | 26S | 48W |
| Minuca mordax | LC782418 | Palmital, Santa Catarina, Brazil | 26S | 48W |
| Minuca mordax | LC782419 | Palmital, Santa Catarina, Brazil | 26S | 48W |
| Minuca mordax | LC782420 | Palmital, Santa Catarina, Brazil | 26S | 48W |
| Minuca mordax | LC782421 | Palmital, Santa Catarina, Brazil | 26S | 48W |
| Minuca mordax | LC782422 | Palmital, Santa Catarina, Brazil | 26S | 48W |
| Minuca mordax | LC782423 | Palmital, Santa Catarina, Brazil | 26S | 48W |
| Minuca mordax | LC782424 | Palmital, Santa Catarina, Brazil | 26S | 48W |
| Minuca mordax | LC782425 | Palmital, Santa Catarina, Brazil | 26S | 48W |
| Minuca mordax | LC782426 | Palmital, Santa Catarina, Brazil | 26S | 48W |
| Minuca mordax | LC782427 | Palmital, Santa Catarina, Brazil | 26S | 48W |
| Minuca mordax | LC782428 | Torres, Rio Grande do Sul, Brazil | 29S | 49W |
| Minuca mordax | LC782429 | Torres, Rio Grande do Sul, Brazil | 29S | 49W |
| Minuca mordax | LC782430 | Torres, Rio Grande do Sul, Brazil | 29S | 49W |
| Minuca mordax | LC782431 | Torres, Rio Grande do Sul, Brazil | 29S | 49W |
| Minuca mordax | LC782432 | Torres, Rio Grande do Sul, Brazil | 29S | 49W |
| Minuca mordax | LC782433 | Torres, Rio Grande do Sul, Brazil | 29S | 49W |
| Minuca mordax | LC782434 | Torres, Rio Grande do Sul, Brazil | 29S | 49W |
| Minuca mordax | LC782435 | Torres, Rio Grande do Sul, Brazil | 29S | 49W |
| Minuca mordax | LC782436 | Torres, Rio Grande do Sul, Brazil | 29S | 49W |
| Minuca mordax | LC782437 | Torres, Rio Grande do Sul, Brazil | 29S | 49W |
| Minuca mordax | LC782438 | Torres, Rio Grande do Sul, Brazil | 29S | 49W |
| Clibanarius tricolor | MK254020 | Bahia Ronda, Florida, USA | 24N | 81W |
| Clibanarius tricolor | MK254095 | Bahia Ronda, Florida, USA | 24N | 81W |
| Clibanarius tricolor | MK254092 | Bahia Ronda, Florida, USA | 24N | 81W |
| Clibanarius tricolor | MK254062 | Bahia Ronda, Florida, USA | 24N | 81W |
| Clibanarius tricolor | MK254053 | Bahia Ronda, Florida, USA | 24N | 81W |
| Clibanarius tricolor | MK254103 | Bahia Ronda, Florida, USA | 24N | 81W |
| Clibanarius tricolor | MK253982 | Bahia Ronda, Florida, USA | 24N | 81W |
| Clibanarius tricolor | MK253991 | Bahia Ronda, Florida, USA | 24N | 81W |
| Clibanarius tricolor | MK254054 | Bahia Ronda, Florida, USA | 24N | 81W |
| Clibanarius tricolor | MK253978 | Bahia Ronda, Florida, USA | 24N | 81W |
| Clibanarius tricolor | MK254104 | Bahia Ronda, Florida, USA | 24N | 81W |
| Clibanarius tricolor | MK254134 | Bahia Ronda, Florida, USA | 24N | 81W |
| Clibanarius tricolor | MK254109 | Bahia Ronda, Florida, USA | 24N | 81W |
| Clibanarius tricolor | MK253984 | Bahia Ronda, Florida, USA | 24N | 81W |
| Clibanarius tricolor | MK254090 | Bahia Ronda, Florida, USA | 24N | 81W |
| Clibanarius tricolor | MK254135 | Ducke Keys, Florida, USA | 24N | 81W |
| Clibanarius tricolor | MK254071 | Ducke Keys, Florida, USA | 24N | 81W |
| Clibanarius tricolor | MK253992 | Ducke Keys, Florida, USA | 24N | 81W |
| Clibanarius tricolor | MK254063 | Ducke Keys, Florida, USA | 24N | 81W |
| Clibanarius tricolor | MK254100 | Ducke Keys, Florida, USA | 24N | 81W |
| Clibanarius tricolor | MK254016 | Ducke Keys, Florida, USA | 24N | 81W |
| Clibanarius tricolor | MK254040 | Ducke Keys, Florida, USA | 24N | 81W |
| Clibanarius tricolor | MK254059 | Ducke Keys, Florida, USA | 24N | 81W |
| Clibanarius tricolor | MK254083 | Ducke Keys, Florida, USA | 24N | 81W |
| Clibanarius tricolor | MK254094 | Ducke Keys, Florida, USA | 24N | 81W |
| Clibanarius tricolor | MK253994 | Ducke Keys, Florida, USA | 24N | 81W |
| Clibanarius tricolor | MK254102 | Sombrero Key, Florida, USA | 24N | 81W |
| Clibanarius tricolor | MK254035 | Sombrero Key, Florida, USA | 24N | 81W |
| Clibanarius tricolor | MK254066 | Florida Keys, Florida, USA | 25N | 80W |
| Clibanarius tricolor | MK254128 | Florida Keys, Florida, USA | 25N | 80W |
| Clibanarius tricolor | MK254123 | Florida Keys, Florida, USA | 25N | 80W |
| Clibanarius tricolor | MK254082 | Florida Keys, Florida, USA | 25N | 80W |
| Clibanarius tricolor | MK254010 | Florida Keys, Florida, USA | 25N | 80W |
| Clibanarius tricolor | MK254099 | Florida Keys, Florida, USA | 25N | 80W |
| Clibanarius tricolor | MK254067 | Florida Keys, Florida, USA | 25N | 80W |
| Clibanarius tricolor | MK254011 | Florida Keys, Florida, USA | 25N | 80W |
| Clibanarius tricolor | MK254001 | Florida Keys, Florida, USA | 25N | 80W |
| Clibanarius tricolor | MK254061 | Florida Keys, Florida, USA | 25N | 80W |
| Clibanarius tricolor | MK254120 | Florida Keys, Florida, USA | 25N | 80W |
| Clibanarius tricolor | MK254119 | Florida Keys, Florida, USA | 25N | 80W |
| Clibanarius tricolor | MK254042 | Florida Keys, Florida, USA | 25N | 80W |
| Clibanarius tricolor | MK254003 | New Providence Island, Bahamas | 24N | 70W |
| Clibanarius tricolor | MK253986 | New Providence Island, Bahamas | 24N | 70W |
| Clibanarius tricolor | MK254118 | New Providence Island, Bahamas | 24N | 70W |
| Clibanarius tricolor | MK253989 | New Providence Island, Bahamas | 24N | 70W |
| Clibanarius tricolor | MK254106 | New Providence Island, Bahamas | 24N | 70W |
| Clibanarius tricolor | MK254028 | New Providence Island, Bahamas | 24N | 70W |
| Clibanarius tricolor | MK253999 | New Providence Island, Bahamas | 24N | 70W |
| Clibanarius tricolor | MK254073 | New Providence Island, Bahamas | 24N | 70W |
| Clibanarius tricolor | MK254116 | New Providence Island, Bahamas | 24N | 70W |
| Clibanarius tricolor | MK254137 | New Providence Island, Bahamas | 24N | 70W |
| Clibanarius tricolor | MK254124 | New Providence Island, Bahamas | 24N | 70W |
| Clibanarius tricolor | MK254008 | New Providence Island, Bahamas | 24N | 70W |
| Clibanarius tricolor | MK254023 | New Providence Island, Bahamas | 24N | 70W |
| Clibanarius tricolor | MK254132 | New Providence Island, Bahamas | 24N | 70W |
| Clibanarius tricolor | MK254130 | New Providence Island, Bahamas | 24N | 70W |
| Clibanarius tricolor | MK254084 | New Providence Island, Bahamas | 24N | 70W |
| Clibanarius tricolor | MK254129 | New Providence Island, Bahamas | 24N | 70W |
| Clibanarius tricolor | MK254101 | San Salvador Island, Bahamas | 24N | 74W |
| Clibanarius tricolor | MK254058 | San Salvador Island, Bahamas | 24N | 74W |
| Clibanarius tricolor | MK253983 | San Salvador Island, Bahamas | 24N | 74W |
| Clibanarius tricolor | MK254018 | San Salvador Island, Bahamas | 24N | 74W |
| Clibanarius tricolor | MK253985 | San Salvador Island, Bahamas | 24N | 74W |
| Clibanarius tricolor | MK254039 | San Salvador Island, Bahamas | 24N | 74W |
| Clibanarius tricolor | MK254125 | San Salvador Island, Bahamas | 24N | 74W |
| Clibanarius tricolor | MK254034 | San Salvador Island, Bahamas | 24N | 74W |
| Clibanarius tricolor | MK254004 | San Salvador Island, Bahamas | 24N | 74W |
| Clibanarius tricolor | MK254077 | San Salvador Island, Bahamas | 24N | 74W |
| Clibanarius tricolor | MK254121 | San Salvador Island, Bahamas | 24N | 74W |
| Clibanarius tricolor | MK253990 | San Salvador Island, Bahamas | 24N | 74W |
| Clibanarius tricolor | MK254139 | San Salvador Island, Bahamas | 24N | 74W |
| Clibanarius tricolor | MK254079 | San Salvador Island, Bahamas | 24N | 74W |
| Clibanarius tricolor | MK253981 | San Salvador Island, Bahamas | 24N | 74W |
| Clibanarius tricolor | MK253997 | San Salvador Island, Bahamas | 24N | 74W |
| Clibanarius tricolor | MK253974 | San Salvador Island, Bahamas | 24N | 74W |
| Clibanarius tricolor | MK254017 | San Salvador Island, Bahamas | 24N | 74W |
| Clibanarius tricolor | MK254069 | San Salvador Island, Bahamas | 24N | 74W |
| Clibanarius tricolor | MK254097 | San Salvador Island, Bahamas | 24N | 74W |
| Clibanarius tricolor | MK254114 | Arecibo, Puerto Rico | 17N | 66W |
| Clibanarius tricolor | MK254096 | Arecibo, Puerto Rico | 17N | 66W |
| Clibanarius tricolor | MK254033 | Arecibo, Puerto Rico | 17N | 66W |
| Clibanarius tricolor | MK253998 | Arecibo, Puerto Rico | 17N | 66W |
| Clibanarius tricolor | MK254098 | Arecibo, Puerto Rico | 17N | 66W |
| Clibanarius tricolor | MK253975 | Arecibo, Puerto Rico | 17N | 66W |
| Clibanarius tricolor | MK253976 | Arecibo, Puerto Rico | 17N | 66W |
| Clibanarius tricolor | MK254126 | Arecibo, Puerto Rico | 17N | 66W |
| Clibanarius tricolor | MK254029 | Arecibo, Puerto Rico | 17N | 66W |
| Clibanarius tricolor | MK254086 | Arecibo, Puerto Rico | 17N | 66W |
| Clibanarius tricolor | MK254024 | Arecibo, Puerto Rico | 17N | 66W |
| Clibanarius tricolor | MK254091 | Arecibo, Puerto Rico | 17N | 66W |
| Clibanarius tricolor | MK253977 | Arecibo, Puerto Rico | 17N | 66W |
| Clibanarius tricolor | MK254110 | Arecibo, Puerto Rico | 17N | 66W |
| Clibanarius tricolor | MK254080 | Arecibo, Puerto Rico | 17N | 66W |
| Clibanarius tricolor | MK254037 | Laurel, Puerto Rico | 17N | 66W |
| Clibanarius tricolor | MK254072 | Laurel, Puerto Rico | 17N | 66W |
| Clibanarius tricolor | MK254057 | Laurel, Puerto Rico | 17N | 66W |
| Clibanarius tricolor | MK254089 | Laurel, Puerto Rico | 17N | 66W |
| Clibanarius tricolor | MK254065 | Laurel, Puerto Rico | 17N | 66W |
| Clibanarius tricolor | MK254044 | Laurel, Puerto Rico | 17N | 66W |
| Clibanarius tricolor | MK254019 | Laurel, Puerto Rico | 17N | 66W |
| Clibanarius tricolor | MK254076 | Laurel, Puerto Rico | 17N | 66W |
| Clibanarius tricolor | MK254000 | Laurel, Puerto Rico | 17N | 66W |
| Clibanarius tricolor | MK254105 | Laurel, Puerto Rico | 17N | 66W |
| Clibanarius tricolor | MK254051 | Laurel, Puerto Rico | 17N | 66W |
| Clibanarius tricolor | MK254088 | Laurel, Puerto Rico | 17N | 66W |
| Clibanarius tricolor | MK254027 | Bonaire | 12N | 68W |
| Clibanarius tricolor | MK254108 | Bonaire | 12N | 68W |
| Clibanarius tricolor | MK254041 | Bonaire | 12N | 68W |
| Clibanarius tricolor | MK254005 | Bonaire | 12N | 68W |
| Clibanarius tricolor | MK254043 | Bonaire | 12N | 68W |
| Clibanarius tricolor | MK254012 | Bonaire | 12N | 68W |
| Clibanarius tricolor | MK254081 | Bonaire | 12N | 68W |
| Clibanarius tricolor | MK253996 | Bonaire | 12N | 68W |
| Clibanarius tricolor | MK254136 | Bonaire | 12N | 68W |
| Clibanarius tricolor | MK254002 | Bonaire | 12N | 68W |
| Clibanarius tricolor | MK254055 | Coral View Resort, Honduras | 16N | 86W |
| Clibanarius tricolor | MK254009 | Coral View Resort, Honduras | 16N | 86W |
| Clibanarius tricolor | MK254115 | Coral View Resort, Honduras | 16N | 86W |
| Clibanarius tricolor | MK254013 | Coral View Resort, Honduras | 16N | 86W |
| Clibanarius tricolor | MK254025 | Coral View Resort, Honduras | 16N | 86W |
| Clibanarius tricolor | MK254122 | Coral View Resort, Honduras | 16N | 86W |
| Clibanarius tricolor | MK254032 | Coral View Resort, Honduras | 16N | 86W |
| Clibanarius tricolor | MK254015 | Coral View Resort, Honduras | 16N | 86W |
| Clibanarius tricolor | MK254036 | Coral View Resort, Honduras | 16N | 86W |
| Clibanarius tricolor | MK254060 | Coral View Resort, Honduras | 16N | 86W |
| Clibanarius tricolor | MK253993 | Coral View Resort, Honduras | 16N | 86W |
| Clibanarius tricolor | MK254085 | Coral View Resort, Honduras | 16N | 86W |
| Clibanarius tricolor | MK254068 | Coral View Resort, Honduras | 16N | 86W |
| Clibanarius tricolor | MK254049 | Eco Marine Diver, Honduras | 16N | 86W |
| Clibanarius tricolor | MK253979 | Eco Marine Diver, Honduras | 16N | 86W |
| Clibanarius tricolor | MK254117 | Eco Marine Diver, Honduras | 16N | 86W |
| Clibanarius tricolor | MK254014 | Eco Marine Diver, Honduras | 16N | 86W |
| Clibanarius tricolor | MK254133 | Eco Marine Diver, Honduras | 16N | 86W |
| Clibanarius tricolor | MK254070 | Eco Marine Diver, Honduras | 16N | 86W |
| Clibanarius tricolor | MK254064 | Eco Marine Diver, Honduras | 16N | 86W |
| Clibanarius tricolor | MK254093 | Eco Marine Diver, Honduras | 16N | 86W |
| Clibanarius tricolor | MK254107 | Eco Marine Diver, Honduras | 16N | 86W |
| Clibanarius tricolor | MK254111 | Eco Marine Diver, Honduras | 16N | 86W |
| Clibanarius tricolor | MK254052 | Eco Marine Diver, Honduras | 16N | 86W |
| Clibanarius tricolor | MK254056 | Eco Marine Diver, Honduras | 16N | 86W |
| Clibanarius tricolor | MK254047 | Eco Marine Diver, Honduras | 16N | 86W |
| Clibanarius tricolor | MK254131 | Eco Marine Diver, Honduras | 16N | 86W |
| Clibanarius tricolor | MK254038 | Belize | 17N | 88W |
| Clibanarius tricolor | MK254127 | Belize | 17N | 88W |
| Clibanarius tricolor | MK254031 | Belize | 17N | 88W |
| Clibanarius tricolor | MK254045 | Belize | 17N | 88W |
| Clibanarius tricolor | MK254030 | Belize | 17N | 88W |
| Clibanarius tricolor | MK254113 | Belize | 17N | 88W |
| Clibanarius tricolor | MK254021 | Belize | 17N | 88W |
| Clibanarius tricolor | MK254140 | Belize | 17N | 88W |
| Clibanarius tricolor | MK254046 | Belize | 17N | 88W |
| Clibanarius tricolor | MK254078 | Yucatan, Mexico | 21N | 90W |
| Clibanarius tricolor | MK254074 | Yucatan, Mexico | 21N | 90W |
| Clibanarius tricolor | MK254075 | Yucatan, Mexico | 21N | 90W |
| Clibanarius tricolor | MK254022 | Yucatan, Mexico | 21N | 90W |
| Clibanarius tricolor | MK253980 | Yucatan, Mexico | 21N | 90W |
| Clibanarius tricolor | MK253995 | Yucatan, Mexico | 21N | 90W |
| Clibanarius tricolor | MK254050 | Yucatan, Mexico | 21N | 90W |
| Clibanarius tricolor | MK254026 | Yucatan, Mexico | 21N | 90W |
| Clibanarius tricolor | MK253988 | Yucatan, Mexico | 21N | 90W |
| Clibanarius tricolor | MK254112 | Yucatan, Mexico | 21N | 90W |
| Clibanarius tricolor | MK254138 | Mahahual, Mexico | 18N | 87W |
| Clibanarius tricolor | MK254006 | Mahahual, Mexico | 18N | 87W |
| Clibanarius tricolor | MK254087 | Mahahual, Mexico | 18N | 87W |
| Clibanarius tricolor | MK254007 | Mahahual, Mexico | 18N | 87W |
| Clibanarius tricolor | MK253987 | Mahahual, Mexico | 18N | 87W |
| Clibanarius tricolor | MK254048 | Mahahual, Mexico | 18N | 87W |
| Panulirus argus | JF921833 | Abaco, Bahamas | 26N | 77W |
| Panulirus argus | JF921534 | Abaco, Bahamas | 26N | 77W |
| Panulirus argus | JF921607 | Abaco, Bahamas | 26N | 77W |
| Panulirus argus | JF921600 | Abaco, Bahamas | 26N | 77W |
| Panulirus argus | JF921608 | Abaco, Bahamas | 26N | 77W |
| Panulirus argus | JF921609 | Abaco, Bahamas | 26N | 77W |
| Panulirus argus | JF921535 | Abaco, Bahamas | 26N | 77W |
| Panulirus argus | JF921536 | Abaco, Bahamas | 26N | 77W |
| Panulirus argus | JF921610 | Abaco, Bahamas | 26N | 77W |
| Panulirus argus | JF921611 | Abaco, Bahamas | 26N | 77W |
| Panulirus argus | JF921820 | Abaco, Bahamas | 26N | 77W |
| Panulirus argus | JF921823 | Abaco, Bahamas | 26N | 77W |
| Panulirus argus | JF921612 | Abaco, Bahamas | 26N | 77W |
| Panulirus argus | JF921613 | Abaco, Bahamas | 26N | 77W |
| Panulirus argus | JF921824 | Abaco, Bahamas | 26N | 77W |
| Panulirus argus | JF921614 | Abaco, Bahamas | 26N | 77W |
| Panulirus argus | JF921615 | Abaco, Bahamas | 26N | 77W |
| Panulirus argus | JF921616 | Abaco, Bahamas | 26N | 77W |
| Panulirus argus | JF921617 | Abaco, Bahamas | 26N | 77W |
| Panulirus argus | JF921618 | Abaco, Bahamas | 26N | 77W |
| Panulirus argus | JF921855 | Bimini, Bahamas | 25N | 79W |
| Panulirus argus | JF921619 | Bimini, Bahamas | 25N | 79W |
| Panulirus argus | JF921620 | Bimini, Bahamas | 25N | 79W |
| Panulirus argus | JF921621 | Bimini, Bahamas | 25N | 79W |
| Panulirus argus | JF921622 | Bimini, Bahamas | 25N | 79W |
| Panulirus argus | JF921623 | Bimini, Bahamas | 25N | 79W |
| Panulirus argus | JF921856 | Bimini, Bahamas | 25N | 79W |
| Panulirus argus | JF921624 | Bimini, Bahamas | 25N | 79W |
| Panulirus argus | JF921625 | Bimini, Bahamas | 25N | 79W |
| Panulirus argus | JF921626 | Bimini, Bahamas | 25N | 79W |
| Panulirus argus | JF921585 | Bimini, Bahamas | 25N | 79W |
| Panulirus argus | JF921797 | Bimini, Bahamas | 25N | 79W |
| Panulirus argus | JF921798 | Bimini, Bahamas | 25N | 79W |
| Panulirus argus | JF921627 | Bimini, Bahamas | 25N | 79W |
| Panulirus argus | JF921628 | Bimini, Bahamas | 25N | 79W |
| Panulirus argus | JF921629 | Bimini, Bahamas | 25N | 79W |
| Panulirus argus | JF921807 | Bimini, Bahamas | 25N | 79W |
| Panulirus argus | JF921838 | Bimini, Bahamas | 25N | 79W |
| Panulirus argus | JF921630 | Bimini, Bahamas | 25N | 79W |
| Panulirus argus | JF921817 | Bimini, Bahamas | 25N | 79W |
| Panulirus argus | JF921631 | Bimini, Bahamas | 25N | 79W |
| Panulirus argus | JF921839 | Bimini, Bahamas | 25N | 79W |
| Panulirus argus | JF921821 | Bimini, Bahamas | 25N | 79W |
| Panulirus argus | JF921632 | Bimini, Bahamas | 25N | 79W |
| Panulirus argus | JF921633 | Bimini, Bahamas | 25N | 79W |
| Panulirus argus | JF921634 | Bimini, Bahamas | 25N | 79W |
| Panulirus argus | JF921537 | Bimini, Bahamas | 25N | 79W |
| Panulirus argus | JF921566 | Bimini, Bahamas | 25N | 79W |
| Panulirus argus | JF921635 | Bimini, Bahamas | 25N | 79W |
| Panulirus argus | JF921636 | Bimini, Bahamas | 25N | 79W |
| Panulirus argus | JF921637 | Bimini, Bahamas | 25N | 79W |
| Panulirus argus | JF921857 | Bimini, Bahamas | 25N | 79W |
| Panulirus argus | JF921638 | Bimini, Bahamas | 25N | 79W |
| Panulirus argus | JF921639 | Bimini, Bahamas | 25N | 79W |
| Panulirus argus | JF921813 | Bimini, Bahamas | 25N | 79W |
| Panulirus argus | JF921801 | Bimini, Bahamas | 25N | 79W |
| Panulirus argus | JF921640 | Bimini, Bahamas | 25N | 79W |
| Panulirus argus | JF921641 | Bimini, Bahamas | 25N | 79W |
| Panulirus argus | JF921642 | Bimini, Bahamas | 25N | 79W |
| Panulirus argus | JF921643 | Bimini, Bahamas | 25N | 79W |
| Panulirus argus | JF921601 | Bimini, Bahamas | 25N | 79W |
| Panulirus argus | JF921538 | Bimini, Bahamas | 25N | 79W |
| Panulirus argus | JF921858 | Bimini, Bahamas | 25N | 79W |
| Panulirus argus | JF921539 | Bimini, Bahamas | 25N | 79W |
| Panulirus argus | JF921644 | Bimini, Bahamas | 25N | 79W |
| Panulirus argus | JF921584 | Bimini, Bahamas | 25N | 79W |
| Panulirus argus | JF921540 | Bimini, Bahamas | 25N | 79W |
| Panulirus argus | JF921583 | Bimini, Bahamas | 25N | 79W |
| Panulirus argus | JF921645 | Bimini, Bahamas | 25N | 79W |
| Panulirus argus | JF921594 | Bimini, Bahamas | 25N | 79W |
| Panulirus argus | JF921646 | Bimini, Bahamas | 25N | 79W |
| Panulirus argus | JF921647 | Bimini, Bahamas | 25N | 79W |
| Panulirus argus | JF921648 | Bimini, Bahamas | 25N | 79W |
| Panulirus argus | JF921595 | Bimini, Bahamas | 25N | 79W |
| Panulirus argus | JF921649 | Bimini, Bahamas | 25N | 79W |
| Panulirus argus | JF921650 | Bimini, Bahamas | 25N | 79W |
| Panulirus argus | JF921651 | Bimini, Bahamas | 25N | 79W |
| Panulirus argus | JF921652 | Bimini, Bahamas | 25N | 79W |
| Panulirus argus | JF921653 | Bimini, Bahamas | 25N | 79W |
| Panulirus argus | JF921822 | Bimini, Bahamas | 25N | 79W |
| Panulirus argus | JF921602 | Bimini, Bahamas | 25N | 79W |
| Panulirus argus | JF921654 | Bimini, Bahamas | 25N | 79W |
| Panulirus argus | JF921655 | Bimini, Bahamas | 25N | 79W |
| Panulirus argus | JF921656 | Exuma Park, Bahamas | 24N | 76W |
| Panulirus argus | JF921581 | Exuma Park, Bahamas | 24N | 76W |
| Panulirus argus | JF921657 | Exuma Park, Bahamas | 24N | 76W |
| Panulirus argus | JF921859 | Exuma Park, Bahamas | 24N | 76W |
| Panulirus argus | JF921658 | Exuma Park, Bahamas | 24N | 76W |
| Panulirus argus | JF921570 | Exuma Park, Bahamas | 24N | 76W |
| Panulirus argus | JF921831 | Exuma Park, Bahamas | 24N | 76W |
| Panulirus argus | JF921659 | Exuma Park, Bahamas | 24N | 76W |
| Panulirus argus | JF921568 | Exuma Park, Bahamas | 24N | 76W |
| Panulirus argus | JF921576 | Exuma Park, Bahamas | 24N | 76W |
| Panulirus argus | JF921541 | Exuma Park, Bahamas | 24N | 76W |
| Panulirus argus | JF921852 | Exuma Park, Bahamas | 24N | 76W |
| Panulirus argus | JF921582 | Exuma Park, Bahamas | 24N | 76W |
| Panulirus argus | JF921660 | Exuma Park, Bahamas | 24N | 76W |
| Panulirus argus | JF921661 | Exuma Park, Bahamas | 24N | 76W |
| Panulirus argus | JF921827 | Exuma Park, Bahamas | 24N | 76W |
| Panulirus argus | JF921662 | Exuma Park, Bahamas | 24N | 76W |
| Panulirus argus | JF921809 | Exuma Park, Bahamas | 24N | 76W |
| Panulirus argus | JF921810 | Exuma Park, Bahamas | 24N | 76W |
| Panulirus argus | JF921542 | Exuma Park, Bahamas | 24N | 76W |
| Panulirus argus | JF921580 | Exuma Park, Bahamas | 24N | 76W |
| Panulirus argus | JF921543 | Exuma Park, Bahamas | 24N | 76W |
| Panulirus argus | JF921663 | Exuma Park, Bahamas | 24N | 76W |
| Panulirus argus | JF921544 | Florida | 26N | 80W |
| Panulirus argus | JF921803 | Florida | 26N | 80W |
| Panulirus argus | JF921664 | Florida | 26N | 80W |
| Panulirus argus | JF921572 | Florida | 26N | 80W |
| Panulirus argus | JF921854 | Florida | 26N | 80W |
| Panulirus argus | JF921665 | Florida | 26N | 80W |
| Panulirus argus | JF921666 | Florida | 26N | 80W |
| Panulirus argus | JF921667 | Florida | 26N | 80W |
| Panulirus argus | JF921840 | Florida | 26N | 80W |
| Panulirus argus | JF921668 | Florida | 26N | 80W |
| Panulirus argus | JF921669 | Florida | 26N | 80W |
| Panulirus argus | JF921603 | Florida | 26N | 80W |
| Panulirus argus | JF921670 | Florida | 26N | 80W |
| Panulirus argus | JF921671 | Florida | 26N | 80W |
| Panulirus argus | JF921545 | Florida | 26N | 80W |
| Panulirus argus | JF921596 | Florida | 26N | 80W |
| Panulirus argus | JF921672 | Florida | 26N | 80W |
| Panulirus argus | JF921673 | Florida | 26N | 80W |
| Panulirus argus | JF921815 | Florida | 26N | 80W |
| Panulirus argus | JF921819 | Florida | 26N | 80W |
| Panulirus argus | JF921674 | Florida | 26N | 80W |
| Panulirus argus | JF921818 | Florida | 26N | 80W |
| Panulirus argus | JF921675 | Florida | 26N | 80W |
| Panulirus argus | JF921676 | Florida | 26N | 80W |
| Panulirus argus | JF921834 | Florida | 26N | 80W |
| Panulirus argus | JF921677 | Florida | 26N | 80W |
| Panulirus argus | JF921853 | Florida | 26N | 80W |
| Panulirus argus | JF921802 | Florida | 26N | 80W |
| Panulirus argus | JF921678 | Florida | 26N | 80W |
| Panulirus argus | JF921679 | Lee Stocking Island, Bahamas | 23N | 76W |
| Panulirus argus | JF921680 | Lee Stocking Island, Bahamas | 23N | 76W |
| Panulirus argus | JF921681 | Lee Stocking Island, Bahamas | 23N | 76W |
| Panulirus argus | JF921811 | Lee Stocking Island, Bahamas | 23N | 76W |
| Panulirus argus | JF921682 | Lee Stocking Island, Bahamas | 23N | 76W |
| Panulirus argus | JF921812 | Lee Stocking Island, Bahamas | 23N | 76W |
| Panulirus argus | JF921683 | Lee Stocking Island, Bahamas | 23N | 76W |
| Panulirus argus | JF921684 | Lee Stocking Island, Bahamas | 23N | 76W |
| Panulirus argus | JF921685 | Lee Stocking Island, Bahamas | 23N | 76W |
| Panulirus argus | JF921686 | Lee Stocking Island, Bahamas | 23N | 76W |
| Panulirus argus | JF921687 | Lee Stocking Island, Bahamas | 23N | 76W |
| Panulirus argus | JF921688 | Lee Stocking Island, Bahamas | 23N | 76W |
| Panulirus argus | JF921689 | Lee Stocking Island, Bahamas | 23N | 76W |
| Panulirus argus | JF921690 | Lee Stocking Island, Bahamas | 23N | 76W |
| Panulirus argus | JF921691 | Lee Stocking Island, Bahamas | 23N | 76W |
| Panulirus argus | JF921808 | Lee Stocking Island, Bahamas | 23N | 76W |
| Panulirus argus | JF921546 | Lee Stocking Island, Bahamas | 23N | 76W |
| Panulirus argus | JF921832 | Lee Stocking Island, Bahamas | 23N | 76W |
| Panulirus argus | JF921692 | Lee Stocking Island, Bahamas | 23N | 76W |
| Panulirus argus | JF921693 | Lee Stocking Island, Bahamas | 23N | 76W |
| Panulirus argus | JF921694 | Lee Stocking Island, Bahamas | 23N | 76W |
| Panulirus argus | JF921695 | Lee Stocking Island, Bahamas | 23N | 76W |
| Panulirus argus | JF921845 | Lee Stocking Island, Bahamas | 23N | 76W |
| Panulirus argus | JF921591 | Lee Stocking Island, Bahamas | 23N | 76W |
| Panulirus argus | JF921846 | Lee Stocking Island, Bahamas | 23N | 76W |
| Panulirus argus | JF921696 | Lee Stocking Island, Bahamas | 23N | 76W |
| Panulirus argus | JF921697 | Lee Stocking Island, Bahamas | 23N | 76W |
| Panulirus argus | JF921698 | Lee Stocking Island, Bahamas | 23N | 76W |
| Panulirus argus | JF921699 | Lee Stocking Island, Bahamas | 23N | 76W |
| Panulirus argus | JF921567 | Lee Stocking Island, Bahamas | 23N | 76W |
| Panulirus argus | JF921847 | Lee Stocking Island, Bahamas | 23N | 76W |
| Panulirus argus | JF921574 | Lee Stocking Island, Bahamas | 23N | 76W |
| Panulirus argus | JF921841 | Lee Stocking Island, Bahamas | 23N | 76W |
| Panulirus argus | JF921842 | Lee Stocking Island, Bahamas | 23N | 76W |
| Panulirus argus | JF921575 | Lee Stocking Island, Bahamas | 23N | 76W |
| Panulirus argus | JF921700 | Lee Stocking Island, Bahamas | 23N | 76W |
| Panulirus argus | JF921701 | Lee Stocking Island, Bahamas | 23N | 76W |
| Panulirus argus | JF921702 | Lee Stocking Island, Bahamas | 23N | 76W |
| Panulirus argus | JF921586 | Lee Stocking Island, Bahamas | 23N | 76W |
| Panulirus argus | JF921703 | Lee Stocking Island, Bahamas | 23N | 76W |
| Panulirus argus | JF921704 | Lee Stocking Island, Bahamas | 23N | 76W |
| Panulirus argus | JF921705 | Lee Stocking Island, Bahamas | 23N | 76W |
| Panulirus argus | JF921706 | Lee Stocking Island, Bahamas | 23N | 76W |
| Panulirus argus | JF921795 | Lee Stocking Island, Bahamas | 23N | 76W |
| Panulirus argus | JF921707 | Lee Stocking Island, Bahamas | 23N | 76W |
| Panulirus argus | JF921796 | Lee Stocking Island, Bahamas | 23N | 76W |
| Panulirus argus | JF921708 | Lee Stocking Island, Bahamas | 23N | 76W |
| Panulirus argus | JF921709 | Lee Stocking Island, Bahamas | 23N | 76W |
| Panulirus argus | JF921710 | Lee Stocking Island, Bahamas | 23N | 76W |
| Panulirus argus | JF921711 | Lee Stocking Island, Bahamas | 23N | 76W |
| Panulirus argus | JF921712 | Lee Stocking Island, Bahamas | 23N | 76W |
| Panulirus argus | JF921713 | Lee Stocking Island, Bahamas | 23N | 76W |
| Panulirus argus | JF921592 | Lee Stocking Island, Bahamas | 23N | 76W |
| Panulirus argus | JF921547 | Lee Stocking Island, Bahamas | 23N | 76W |
| Panulirus argus | JF921848 | Lee Stocking Island, Bahamas | 23N | 76W |
| Panulirus argus | JF921714 | Lee Stocking Island, Bahamas | 23N | 76W |
| Panulirus argus | JF921715 | Lee Stocking Island, Bahamas | 23N | 76W |
| Panulirus argus | JF921716 | Lee Stocking Island, Bahamas | 23N | 76W |
| Panulirus argus | JF921717 | Lee Stocking Island, Bahamas | 23N | 76W |
| Panulirus argus | JF921590 | Lee Stocking Island, Bahamas | 23N | 76W |
| Panulirus argus | JF921548 | Lee Stocking Island, Bahamas | 23N | 76W |
| Panulirus argus | JF921816 | Lee Stocking Island, Bahamas | 23N | 76W |
| Panulirus argus | JF921549 | Lee Stocking Island, Bahamas | 23N | 76W |
| Panulirus argus | JF921718 | Lee Stocking Island, Bahamas | 23N | 76W |
| Panulirus argus | JF921719 | North Andros, Bahamas | 24N | 78W |
| Panulirus argus | JF921604 | North Andros, Bahamas | 24N | 78W |
| Panulirus argus | JF921720 | North Andros, Bahamas | 24N | 78W |
| Panulirus argus | JF921605 | North Andros, Bahamas | 24N | 78W |
| Panulirus argus | JF921721 | North Andros, Bahamas | 24N | 78W |
| Panulirus argus | JF921589 | North Andros, Bahamas | 24N | 78W |
| Panulirus argus | JF921722 | North Andros, Bahamas | 24N | 78W |
| Panulirus argus | JF921723 | North Andros, Bahamas | 24N | 78W |
| Panulirus argus | JF921835 | North Andros, Bahamas | 24N | 78W |
| Panulirus argus | JF921724 | North Andros, Bahamas | 24N | 78W |
| Panulirus argus | JF921830 | North Andros, Bahamas | 24N | 78W |
| Panulirus argus | JF921725 | North Andros, Bahamas | 24N | 78W |
| Panulirus argus | JF921726 | North Andros, Bahamas | 24N | 78W |
| Panulirus argus | JF921727 | North Andros, Bahamas | 24N | 78W |
| Panulirus argus | JF921728 | North Andros, Bahamas | 24N | 78W |
| Panulirus argus | JF921729 | North Andros, Bahamas | 24N | 78W |
| Panulirus argus | JF921550 | North Andros, Bahamas | 24N | 78W |
| Panulirus argus | JF921730 | North Andros, Bahamas | 24N | 78W |
| Panulirus argus | JF921731 | North Andros, Bahamas | 24N | 78W |
| Panulirus argus | JF921732 | North Andros, Bahamas | 24N | 78W |
| Panulirus argus | JF921733 | North Andros, Bahamas | 24N | 78W |
| Panulirus argus | JF921734 | North Andros, Bahamas | 24N | 78W |
| Panulirus argus | JF921735 | North Andros, Bahamas | 24N | 78W |
| Panulirus argus | JF921551 | North Andros, Bahamas | 24N | 78W |
| Panulirus argus | JF921736 | North Andros, Bahamas | 24N | 78W |
| Panulirus argus | JF921737 | North Andros, Bahamas | 24N | 78W |
| Panulirus argus | JF921843 | North Andros, Bahamas | 24N | 78W |
| Panulirus argus | JF921738 | North Andros, Bahamas | 24N | 78W |
| Panulirus argus | JF921739 | North Andros, Bahamas | 24N | 78W |
| Panulirus argus | JF921740 | North Andros, Bahamas | 24N | 78W |
| Panulirus argus | JF921741 | North Andros, Bahamas | 24N | 78W |
| Panulirus argus | JF921597 | North Andros, Bahamas | 24N | 78W |
| Panulirus argus | JF921742 | North Andros, Bahamas | 24N | 78W |
| Panulirus argus | JF921743 | North Andros, Bahamas | 24N | 78W |
| Panulirus argus | JF921744 | North Andros, Bahamas | 24N | 78W |
| Panulirus argus | JF921606 | North Andros, Bahamas | 24N | 78W |
| Panulirus argus | JF921745 | North Andros, Bahamas | 24N | 78W |
| Panulirus argus | JF921746 | North Andros, Bahamas | 24N | 78W |
| Panulirus argus | JF921828 | North Andros, Bahamas | 24N | 78W |
| Panulirus argus | JF921747 | North Andros, Bahamas | 24N | 78W |
| Panulirus argus | JF921748 | North Andros, Bahamas | 24N | 78W |
| Panulirus argus | JF921849 | North Andros, Bahamas | 24N | 78W |
| Panulirus argus | JF921749 | North Andros, Bahamas | 24N | 78W |
| Panulirus argus | JF921552 | North Andros, Bahamas | 24N | 78W |
| Panulirus argus | JF921750 | North Andros, Bahamas | 24N | 78W |
| Panulirus argus | JF921751 | North Andros, Bahamas | 24N | 78W |
| Panulirus argus | JF921752 | North Andros, Bahamas | 24N | 78W |
| Panulirus argus | JF921804 | Puerto Rico | 17N | 66W |
| Panulirus argus | JF921553 | Puerto Rico | 17N | 66W |
| Panulirus argus | JF921753 | Puerto Rico | 17N | 66W |
| Panulirus argus | JF921577 | Puerto Rico | 17N | 66W |
| Panulirus argus | JF921554 | Puerto Rico | 17N | 66W |
| Panulirus argus | JF921754 | Puerto Rico | 17N | 66W |
| Panulirus argus | JF921555 | Puerto Rico | 17N | 66W |
| Panulirus argus | JF921755 | Puerto Rico | 17N | 66W |
| Panulirus argus | JF921805 | Puerto Rico | 17N | 66W |
| Panulirus argus | JF921756 | Puerto Rico | 17N | 66W |
| Panulirus argus | JF921556 | Puerto Rico | 17N | 66W |
| Panulirus argus | JF921799 | Puerto Rico | 17N | 66W |
| Panulirus argus | JF921757 | Puerto Rico | 17N | 66W |
| Panulirus argus | JF921758 | Puerto Rico | 17N | 66W |
| Panulirus argus | JF921759 | Puerto Rico | 17N | 66W |
| Panulirus argus | JF921760 | Puerto Rico | 17N | 66W |
| Panulirus argus | JF921761 | Puerto Rico | 17N | 66W |
| Panulirus argus | JF921762 | Puerto Rico | 17N | 66W |
| Panulirus argus | JF921763 | Puerto Rico | 17N | 66W |
| Panulirus argus | JF921764 | Puerto Rico | 17N | 66W |
| Panulirus argus | JF921765 | Puerto Rico | 17N | 66W |
| Panulirus argus | JF921593 | Puerto Rico | 17N | 66W |
| Panulirus argus | JF921766 | Puerto Rico | 17N | 66W |
| Panulirus argus | JF921767 | Puerto Rico | 17N | 66W |
| Panulirus argus | JF921768 | Puerto Rico | 17N | 66W |
| Panulirus argus | JF921769 | Puerto Rico | 17N | 66W |
| Panulirus argus | JF921826 | Puerto Rico | 17N | 66W |
| Panulirus argus | JF921770 | Puerto Rico | 17N | 66W |
| Panulirus argus | JF921771 | Puerto Rico | 17N | 66W |
| Panulirus argus | JF921557 | Puerto Rico | 17N | 66W |
| Panulirus argus | JF921772 | San Salvador, El Salvador | 24N | 74W |
| Panulirus argus | JF921773 | San Salvador, El Salvador | 24N | 74W |
| Panulirus argus | JF921578 | San Salvador, El Salvador | 24N | 74W |
| Panulirus argus | JF921774 | San Salvador, El Salvador | 24N | 74W |
| Panulirus argus | JF921850 | San Salvador, El Salvador | 24N | 74W |
| Panulirus argus | JF921599 | San Salvador, El Salvador | 24N | 74W |
| Panulirus argus | JF921579 | San Salvador, El Salvador | 24N | 74W |
| Panulirus argus | JF921571 | San Salvador, El Salvador | 24N | 74W |
| Panulirus argus | JF921775 | San Salvador, El Salvador | 24N | 74W |
| Panulirus argus | JF921776 | San Salvador, El Salvador | 24N | 74W |
| Panulirus argus | JF921777 | San Salvador, El Salvador | 24N | 74W |
| Panulirus argus | JF921598 | San Salvador, El Salvador | 24N | 74W |
| Panulirus argus | JF921558 | San Salvador, El Salvador | 24N | 74W |
| Panulirus argus | JF921778 | San Salvador, El Salvador | 24N | 74W |
| Panulirus argus | JF921559 | San Salvador, El Salvador | 24N | 74W |
| Panulirus argus | JF921779 | San Salvador, El Salvador | 24N | 74W |
| Panulirus argus | JF921780 | San Salvador, El Salvador | 24N | 74W |
| Panulirus argus | JF921781 | San Salvador, El Salvador | 24N | 74W |
| Panulirus argus | JF921782 | San Salvador, El Salvador | 24N | 74W |
| Panulirus argus | JF921814 | San Salvador, El Salvador | 24N | 74W |
| Panulirus argus | JF921560 | San Salvador, El Salvador | 24N | 74W |
| Panulirus argus | JF921783 | San Salvador, El Salvador | 24N | 74W |
| Panulirus argus | JF921784 | San Salvador, El Salvador | 24N | 74W |
| Panulirus argus | JF921588 | San Salvador, El Salvador | 24N | 74W |
| Panulirus argus | JF921785 | San Salvador, El Salvador | 24N | 74W |
| Panulirus argus | JF921806 | San Salvador, El Salvador | 24N | 74W |
| Panulirus argus | JF921786 | South Caicos, Turks and Caicos Islands | 21N | 71W |
| Panulirus argus | JF921800 | South Caicos, Turks and Caicos Islands | 21N | 71W |
| Panulirus argus | JF921851 | South Caicos, Turks and Caicos Islands | 21N | 71W |
| Panulirus argus | JF921844 | South Caicos, Turks and Caicos Islands | 21N | 71W |
| Panulirus argus | JF921561 | South Caicos, Turks and Caicos Islands | 21N | 71W |
| Panulirus argus | JF921569 | South Caicos, Turks and Caicos Islands | 21N | 71W |
| Panulirus argus | JF921562 | South Caicos, Turks and Caicos Islands | 21N | 71W |
| Panulirus argus | JF921787 | South Caicos, Turks and Caicos Islands | 21N | 71W |
| Panulirus argus | JF921788 | South Caicos, Turks and Caicos Islands | 21N | 71W |
| Panulirus argus | JF921789 | South Caicos, Turks and Caicos Islands | 21N | 71W |
| Panulirus argus | JF921837 | South Caicos, Turks and Caicos Islands | 21N | 71W |
| Panulirus argus | JF921563 | South Caicos, Turks and Caicos Islands | 21N | 71W |
| Panulirus argus | JF921836 | South Caicos, Turks and Caicos Islands | 21N | 71W |
| Panulirus argus | JF921790 | South Caicos, Turks and Caicos Islands | 21N | 71W |
| Panulirus argus | JF921564 | South Caicos, Turks and Caicos Islands | 21N | 71W |
| Panulirus argus | JF921791 | South Caicos, Turks and Caicos Islands | 21N | 71W |
| Panulirus argus | JF921587 | South Caicos, Turks and Caicos Islands | 21N | 71W |
| Panulirus argus | JF921792 | South Caicos, Turks and Caicos Islands | 21N | 71W |
| Panulirus argus | JF921573 | South Caicos, Turks and Caicos Islands | 21N | 71W |
| Panulirus argus | JF921829 | South Caicos, Turks and Caicos Islands | 21N | 71W |
| Panulirus argus | JF921825 | South Caicos, Turks and Caicos Islands | 21N | 71W |
| Panulirus argus | JF921565 | South Caicos, Turks and Caicos Islands | 21N | 71W |
| Panulirus argus | JF921793 | South Caicos, Turks and Caicos Islands | 21N | 71W |
| Panulirus argus | JF921794 | South Caicos, Turks and Caicos Islands | 21N | 71W |
| Clibanarius antillensis | MG264471 | Veracruz, Mexico | 18N | 85W |
| Clibanarius antillensis | MT740091 | Veracruz, Mexico | 18N | 85W |
| Clibanarius antillensis | MG264472 | Veracruz, Mexico | 18N | 85W |
| Clibanarius antillensis | MG264474 | Playa Puerto Viejo, Costa Rica | 9N | 82W |
| Clibanarius antillensis | MG264475 | Playa Puerto Viejo, Costa Rica | 9N | 82W |
| Clibanarius antillensis | MG264476 | Playa Puerto Viejo, Costa Rica | 9N | 82W |
| Clibanarius antillensis | MG264477 | Bocas del Toro, Panama | 9N | 82W |
| Clibanarius antillensis | MG264478 | Bocas del Toro, Panama | 9N | 82W |
| Clibanarius antillensis | MN183884 | Bocas del Toro, Panama | 9N | 82W |
| Clibanarius antillensis | MN183927 | Bocas del Toro, Panama | 9N | 82W |
| Clibanarius antillensis | MN184016 | Bocas del Toro, Panama | 9N | 82W |
| Clibanarius antillensis | MG264488 | Touros, Rio Grande do Norte, Brazil | 5S | 35W |
| Clibanarius antillensis | MG264486 | Touros, Rio Grande do Norte, Brazil | 5S | 35W |
| Clibanarius antillensis | MG264487 | Touros, Rio Grande do Norte, Brazil | 5S | 35W |
| Clibanarius antillensis | MG264489 | Ipojuca, Pernambuco, Brazil | 8S | 34W |
| Clibanarius antillensis | MG264490 | Ipojuca, Pernambuco, Brazil | 8S | 34W |
| Clibanarius antillensis | MG264491 | Ipojuca, Pernambuco, Brazil | 8S | 34W |
| Clibanarius antillensis | MG264492 | Ipojuca, Pernambuco, Brazil | 8S | 34W |
| Clibanarius antillensis | MG264493 | Ipojuca, Pernambuco, Brazil | 8S | 34W |
| Clibanarius antillensis | MG264494 | Maragogi, Alagoas, Brazil | 9S | 35W |
| Clibanarius antillensis | MG264495 | Maragogi, Alagoas, Brazil | 9S | 35W |
| Clibanarius antillensis | MG264496 | Maragogi, Alagoas, Brazil | 9S | 35W |
| Clibanarius antillensis | MG264483 | Trairi, Ceará, Brazil | 3S | 38W |
| Clibanarius antillensis | MG264484 | Trairi, Ceará, Brazil | 3S | 38W |
| Clibanarius antillensis | MG264485 | Fortaleza, Ceará, Brazil | 3S | 38W |
| Clibanarius antillensis | MG264501 | Guarapari, Espírito Santo, Brazil | 20S | 40W |
| Clibanarius antillensis | MG264502 | Guarapari, Espírito Santo, Brazil | 20S | 40W |
| Clibanarius antillensis | MG264503 | Guarapari, Espírito Santo, Brazil | 20S | 40W |
| Clibanarius antillensis | MG264504 | Buzios, Rio de Janeiro, Brazil | 22S | 41W |
| Clibanarius antillensis | MG264505 | Buzios, Rio de Janeiro, Brazil | 22S | 41W |
| Clibanarius antillensis | MG264506 | Buzios, Rio de Janeiro, Brazil | 22S | 41W |
| Clibanarius antillensis | MG264508 | Ubatuba, São Paulo, Brazil | 23S | 44W |
| Clibanarius antillensis | MG264507 | Ubatuba, São Paulo, Brazil | 23S | 44W |
| Clibanarius antillensis | MG264509 | Ubatuba, São Paulo, Brazil | 23S | 44W |
| Clibanarius antillensis | MG264510 | Itajaí, Santa Catarina, Brazil | 26S | 48W |
| Clibanarius antillensis | MG264511 | Itajaí, Santa Catarina, Brazil | 26S | 48W |
| Clibanarius antillensis | MG264512 | Itajaí, Santa Catarina, Brazil | 26S | 48W |
| Calcinus tibicen (N) | FJ620372 | Florida, USA | 24N | 81W |
| Calcinus tibicen (N) | FJ620318 | Florida, USA | 27N | 81W |
| Calcinus tibicen (N) | KT897517 | Florida, USA | 27N | 81W |
| Calcinus tibicen (N) | KT897577 | Florida, USA | 27N | 81W |
| Calcinus tibicen (N) | KT897578 | Florida, USA | 27N | 81W |
| Calcinus tibicen (N) | KT897566 | Florida Keys, Florida, USA | 24N | 81W |
| Calcinus tibicen (N) | KT897567 | Florida Keys, Florida, USA | 24N | 81W |
| Calcinus tibicen (N) | KT897579 | Florida Keys, Florida, USA | 24N | 80W |
| Calcinus tibicen (N) | KT897534 | Cozumel, Mexico | 20N | 86W |
| Calcinus tibicen (N) | KT897545 | Cozumel, Mexico | 20N | 86W |
| Calcinus tibicen (N) | KT897581 | Cozumel, Mexico | 20N | 86W |
| Calcinus tibicen (N) | KT897561 | Belize | 16N | 88W |
| Calcinus tibicen (N) | KT897562 | Belize | 16N | 88W |
| Calcinus tibicen (N) | KT897563 | Belize | 16N | 88W |
| Calcinus tibicen (N) | KT897584 | Saint Ann, Jamaica | 17N | 76W |
| Calcinus tibicen (N) | KT897585 | Saint Ann, Jamaica | 17N | 76W |
| Calcinus tibicen (N) | KT897586 | Saint Ann, Jamaica | 17N | 76W |
| Calcinus tibicen (N) | KT897588 | Saint Ann, Jamaica | 17N | 76W |
| Calcinus tibicen (N) | KT897587 | Saint Ann, Jamaica | 17N | 76W |
| Calcinus tibicen (N) | KT897529 | Costa Rica | 9N | 82W |
| Calcinus tibicen (N) | KT897539 | Costa Rica | 9N | 82W |
| Calcinus tibicen (N) | KT897546 | Bocas del Toro, Panama | 9N | 82W |
| Calcinus tibicen (N) | KT897535 | Bocas del Toro, Panama | 9N | 82W |
| Calcinus tibicen (N) | KT897525 | Bocas del Toro, Panama | 9N | 82W |
| Calcinus tibicen (N) | MN183835 | Bocas del Toro, Panama | 9N | 82W |
| Calcinus tibicen (N) | MN183947 | Bocas del Toro, Panama | 9N | 82W |
| Calcinus tibicen (N) | FJ620319 | Tobago, Trinidad & Tobago | 10N | 61W |
| Calcinus tibicen (N) | KT897573 | Bacolet Bay, Trinidad & Tobago | 10N | 61W |
| Calcinus tibicen (N) | KT897574 | Bacolet Bay, Trinidad & Tobago | 10N | 61W |
| Calcinus tibicen (N) | KT897575 | Canoe Bay, Trinidad & Tobago | 10N | 61W |
| Calcinus tibicen (N) | KT897576 | Canoe Bay, Trinidad & Tobago | 10N | 61W |
| Calcinus tibicen (N) | KT897536 | Muelle de la Guardia, Venezuela | 10.6N | 64W |
| Calcinus tibicen (N) | KT897547 | Muelle de la Guardia, Venezuela | 10.6N | 64W |
| Calcinus tibicen (N) | KT897550 | Isla Margarita, Venezuela | 10.6N | 64W |
| Calcinus tibicen (N) | KT897565 | Isla Larga, Venezuela | 10.6N | 64W |
| Calcinus tibicen (N) | KT897564 | Isla Larga, Venezuela | 10.6N | 64W |
| Calcinus tibicen (S) | KT897542 | Praia Meireles, Ceará, Brazil | 3S | 38W |
| Calcinus tibicen (S) | KT897548 | Praia do Pacheco, Ceará, Brazil | 3S | 38W |
| Calcinus tibicen (S) | KT897551 | Praia do Pacheco, Ceará, Brazil | 3S | 38W |
| Calcinus tibicen (S) | KT897543 | Ipojuca, Pernambuco, Brazil | 8S | 34W |
| Calcinus tibicen (S) | KT897549 | Tamandare, Pernambuco, Brazil | 8S | 34W |
| Calcinus tibicen (S) | KT897571 | Recife, Pernambuco, Brazil | 8S | 34W |
| Calcinus tibicen (S) | KT897572 | Recife, Pernambuco, Brazil | 8S | 34W |
| Calcinus tibicen (S) | KT897552 | Recife, Pernambuco, Brazil | 8S | 34W |
| Calcinus tibicen (S) | KT897570 | Recife, Pernambuco, Brazil | 8S | 34W |
| Calcinus tibicen (S) | KT897526 | Maragogi, Alagoas, Brazil | 9S | 35W |
| Calcinus tibicen (S) | KT897540 | Maragogi, Alagoas, Brazil | 9S | 35W |
| Calcinus tibicen (S) | KT897537 | Maragogi, Alagoas, Brazil | 9S | 35W |
| Calcinus tibicen (S) | KT897530 | Guarapari, Espírito Santo, Brazil | 20S | 40W |
| Calcinus tibicen (S) | KT897519 | Guarapari, Espírito Santo, Brazil | 20S | 40W |
| Calcinus tibicen (S) | KT897531 | Iriri, Espírito Santo, Brazil | 20S | 40W |
| Calcinus tibicen (S) | KT897523 | Ilha Anchieta, São Paulo, Brazil | 23.5S | 45W |
| Calcinus tibicen (S) | KT897528 | Ilha Anchieta, São Paulo, Brazil | 23.5S | 45W |
| Calcinus tibicen (S) | KT897544 | Ilha Anchieta, São Paulo, Brazil | 23.5S | 45W |
| Calcinus tibicen (S) | KT897582 | Ilha das Couves, São Paulo, Brazil | 23S | 44W |
| Calcinus tibicen (S) | KT897553 | Ilha da Vitoria, São Paulo, Brazil | 23S | 45W |
| Calcinus tibicen (S) | KT897554 | Ilha da Vitoria, São Paulo, Brazil | 23S | 45W |
| Calcinus tibicen (S) | KT897555 | Ilha da Vitoria, São Paulo, Brazil | 23S | 45W |
| Calcinus tibicen (S) | KT897520 | Ubatuba, São Paulo, Brazil | 23S | 44W |
| Calcinus tibicen (S) | KT897522 | Ubatuba, São Paulo, Brazil | 23S | 44W |
| Calcinus tibicen (S) | KT897527 | Ubatuba, São Paulo, Brazil | 23S | 44W |
| Calcinus tibicen (S) | KT897521 | Bombinhas, Santa Catarina, Brazil | 27S | 48W |
| Calcinus tibicen (S) | KT897524 | Bombinhas, Santa Catarina, Brazil | 27S | 48W |
| Calcinus tibicen (S) | KT897532 | Bombinhas, Santa Catarina, Brazil | 27S | 48W |
| Hippolyte obliquimanus | JF794704 | Playa Cahuita, Limón, Costa Rica | 9N | 82W |
| Hippolyte obliquimanus | JF794705 | Playa Cahuita, Limón, Costa Rica | 9N | 82W |
| Hippolyte obliquimanus | JF794706 | Playa Cahuita, Limón, Costa Rica | 9N | 82W |
| Hippolyte obliquimanus | JF794707 | Playa Cahuita, Limón, Costa Rica | 9N | 82W |
| Hippolyte obliquimanus | JF794708 | Playa Cahuita, Limón, Costa Rica | 9N | 82W |
| Hippolyte obliquimanus | MN183849 | Bocas del Toro, Panama | 9N | 82W |
| Hippolyte obliquimanus | MN183968 | Bocas del Toro, Panama | 9N | 82W |
| Hippolyte obliquimanus | MN184004 | Bocas del Toro, Panama | 9N | 82W |
| Hippolyte obliquimanus | MN184151 | Bocas del Toro, Panama | 9N | 82W |
| Hippolyte obliquimanus | MN184159 | Bocas del Toro, Panama | 9N | 82W |
| Hippolyte obliquimanus | MN184172 | Bocas del Toro, Panama | 9N | 82W |
| Hippolyte obliquimanus | JF794710 | Bocas del Toro, Panama | 9N | 82W |
| Hippolyte obliquimanus | JF794711 | Bocas del Toro, Panama | 9N | 82W |
| Hippolyte obliquimanus | JF794712 | Bocas del Toro, Panama | 9N | 82W |
| Hippolyte obliquimanus | JF794713 | Bocas del Toro, Panama | 9N | 82W |
| Hippolyte obliquimanus | JF794714 | Bocas del Toro, Panama | 9N | 82W |
| Hippolyte obliquimanus | JF794715 | Isla Margarita, Venezuela | 10.6N | 64W |
| Hippolyte obliquimanus | JF794716 | Isla Margarita, Venezuela | 10.6N | 64W |
| Hippolyte obliquimanus | JF794717 | Isla Margarita, Venezuela | 10.6N | 64W |
| Hippolyte obliquimanus | JF794718 | Porto Seguro, Bahia, Brazil | 16S | 39W |
| Hippolyte obliquimanus | JF794719 | Porto Seguro, Bahia, Brazil | 16S | 39W |
| Hippolyte obliquimanus | JF794720 | Porto Seguro, Bahia, Brazil | 16S | 39W |
| Hippolyte obliquimanus | JF794721 | Porto Seguro, Bahia, Brazil | 16S | 39W |
| Hippolyte obliquimanus | JF794722 | Porto Seguro, Bahia, Brazil | 16S | 39W |
| Hippolyte obliquimanus | JF794723 | Ilha Grande, Rio de Janeiro, Brazil | 23S | 44W |
| Hippolyte obliquimanus | JF794724 | Ilha Grande, Rio de Janeiro, Brazil | 23S | 44W |
| Hippolyte obliquimanus | JF794725 | Ilha Grande, Rio de Janeiro, Brazil | 23S | 44W |
| Hippolyte obliquimanus | JF794726 | Ilha Grande, Rio de Janeiro, Brazil | 23S | 44W |
| Hippolyte obliquimanus | JF794727 | Ilha Grande, Rio de Janeiro, Brazil | 23S | 44W |
| Hippolyte obliquimanus | JF794728 | Ubatuba, São Paulo, Brazil | 23S | 44W |
| Hippolyte obliquimanus | JF794729 | Ubatuba, São Paulo, Brazil | 23S | 44W |
| Hippolyte obliquimanus | JF794730 | Ubatuba, São Paulo, Brazil | 23S | 44W |
| Hippolyte obliquimanus | JF794731 | Ubatuba, São Paulo, Brazil | 23S | 44W |
| Hippolyte obliquimanus | JF794732 | Ubatuba, São Paulo, Brazil | 23S | 44W |
| Hippolyte obliquimanus | JF794733 | Florianópolis, Santa Catarina, Brazil | 27S | 48W |
| Hippolyte obliquimanus | JF794734 | Florianópolis, Santa Catarina, Brazil | 27S | 48W |
| Hippolyte obliquimanus | JF794735 | Florianópolis, Santa Catarina, Brazil | 27S | 48W |
| Hippolyte obliquimanus | JF794736 | Florianópolis, Santa Catarina, Brazil | 27S | 48W |
| Hippolyte obliquimanus | JF794737 | Florianópolis, Santa Catarina, Brazil | 27S | 48W |
| Thor amboinensis | MF378828 | Lion Patch, Eleuthera, Bahamas | 24N | 76W |
| Thor amboinensis | MF378829 | Lion Patch, Eleuthera, Bahamas | 24N | 76W |
| Thor amboinensis | MF378830 | Lion Patch, Eleuthera, Bahamas | 24N | 76W |
| Thor amboinensis | MF378831 | Lion Patch, Eleuthera, Bahamas | 24N | 76W |
| Thor amboinensis | MF378832 | Lion Patch, Eleuthera, Bahamas | 24N | 76W |
| Thor amboinensis | MF378833 | Lion Patch, Eleuthera, Bahamas | 24N | 76W |
| Thor amboinensis | MF378834 | Lion Patch, Eleuthera, Bahamas | 24N | 76W |
| Thor amboinensis | MF378835 | Lion Patch, Eleuthera, Bahamas | 24N | 76W |
| Thor amboinensis | MF378836 | Lion Patch, Eleuthera, Bahamas | 24N | 76W |
| Thor amboinensis | MF378837 | Lion Patch, Eleuthera, Bahamas | 24N | 76W |
| Thor amboinensis | MF378838 | Lion Patch, Eleuthera, Bahamas | 24N | 76W |
| Thor amboinensis | MF378839 | Lion Patch, Eleuthera, Bahamas | 24N | 76W |
| Thor amboinensis | MF378840 | Lion Patch, Eleuthera, Bahamas | 24N | 76W |
| Thor amboinensis | MF378841 | Lion Patch, Eleuthera, Bahamas | 24N | 76W |
| Thor amboinensis | MF378842 | Lion Patch, Eleuthera, Bahamas | 24N | 76W |
| Thor amboinensis | MF378843 | Lion Patch, Eleuthera, Bahamas | 24N | 76W |
| Thor amboinensis | MF378844 | Lion Patch, Eleuthera, Bahamas | 24N | 76W |
| Thor amboinensis | MF378845 | Lion Patch, Eleuthera, Bahamas | 24N | 76W |
| Thor amboinensis | MF378846 | Bellairs, Barbados | 13N | 59W |
| Thor amboinensis | MF378847 | Bellairs, Barbados | 13N | 59W |
| Thor amboinensis | MF378848 | Bellairs, Barbados | 13N | 59W |
| Thor amboinensis | MF378849 | Bellairs, Barbados | 13N | 59W |
| Thor amboinensis | MF378850 | Bellairs, Barbados | 13N | 59W |
| Thor amboinensis | MF378851 | Bellairs, Barbados | 13N | 59W |
| Thor amboinensis | MF378852 | Bellairs, Barbados | 13N | 59W |
| Thor amboinensis | MF378853 | Bellairs, Barbados | 13N | 59W |
| Thor amboinensis | MF378854 | Bellairs, Barbados | 13N | 59W |
| Thor amboinensis | MF378855 | Bellairs, Barbados | 13N | 59W |
| Thor amboinensis | MF378856 | Bellairs, Barbados | 13N | 59W |
| Thor amboinensis | MF378857 | Bellairs, Barbados | 13N | 59W |
| Thor amboinensis | MF378858 | Bellairs, Barbados | 13N | 59W |
| Thor amboinensis | MF378859 | Bellairs, Barbados | 13N | 59W |
| Thor amboinensis | MF378860 | Bellairs, Barbados | 13N | 59W |
| Thor amboinensis | MF378861 | Bellairs, Barbados | 13N | 59W |
| Thor amboinensis | MF378862 | Bellairs, Barbados | 13N | 59W |
| Thor amboinensis | MF378863 | Bellairs, Barbados | 13N | 59W |
| Thor amboinensis | MF378864 | Bellairs, Barbados | 13N | 59W |
| Thor amboinensis | MF378865 | Bellairs, Barbados | 13N | 59W |
| Thor amboinensis | MF378866 | Bellairs, Barbados | 13N | 59W |
| Thor amboinensis | MF378867 | Bellairs, Barbados | 13N | 59W |
| Thor amboinensis | MF378868 | Bellairs, Barbados | 13N | 59W |
| Thor amboinensis | MF378869 | Bellairs, Barbados | 13N | 59W |
| Thor amboinensis | MF378870 | Bellairs, Barbados | 13N | 59W |
| Thor amboinensis | MF378871 | Jolly Roger, Barbados | 13N | 59W |
| Thor amboinensis | MF378872 | Jolly Roger, Barbados | 13N | 59W |
| Thor amboinensis | MF378873 | Jolly Roger, Barbados | 13N | 59W |
| Thor amboinensis | MF378874 | Jolly Roger, Barbados | 13N | 59W |
| Thor amboinensis | MF378875 | Jolly Roger, Barbados | 13N | 59W |
| Thor amboinensis | MF378876 | Jolly Roger, Barbados | 13N | 59W |
| Thor amboinensis | MF378877 | Jolly Roger, Barbados | 13N | 59W |
| Thor amboinensis | MF378878 | Jolly Roger, Barbados | 13N | 59W |
| Thor amboinensis | MF378879 | Jolly Roger, Barbados | 13N | 59W |
| Thor amboinensis | MF378880 | Carrie Bow Key, Belize | 16N | 88W |
| Thor amboinensis | MF378881 | Carrie Bow Key, Belize | 16N | 88W |
| Thor amboinensis | MF378882 | Carrie Bow Key, Belize | 16N | 88W |
| Thor amboinensis | MF378883 | Carrie Bow Key, Belize | 16N | 88W |
| Thor amboinensis | MF378884 | Carrie Bow Key, Belize | 16N | 88W |
| Thor amboinensis | MF378885 | Carrie Bow Key, Belize | 16N | 88W |
| Thor amboinensis | MF378886 | Carrie Bow Key, Belize | 16N | 88W |
| Thor amboinensis | MF378887 | Carrie Bow Key, Belize | 16N | 88W |
| Thor amboinensis | MF378888 | Carrie Bow Key, Belize | 16N | 88W |
| Thor amboinensis | MF378889 | Carrie Bow Key, Belize | 16N | 88W |
| Thor amboinensis | MF378890 | Carrie Bow Key, Belize | 16N | 88W |
| Thor amboinensis | MF378891 | Carrie Bow Key, Belize | 16N | 88W |
| Thor amboinensis | MF378892 | Carrie Bow Key, Belize | 16N | 88W |
| Thor amboinensis | MF378915 | Paget Island, Bermuda | 32N | 64W |
| Thor amboinensis | MF378916 | Paget Island, Bermuda | 32N | 64W |
| Thor amboinensis | MF378917 | Paget Island, Bermuda | 32N | 64W |
| Thor amboinensis | MF378918 | Paget Island, Bermuda | 32N | 64W |
| Thor amboinensis | MF378919 | Paget Island, Bermuda | 32N | 64W |
| Thor amboinensis | MF378920 | Paget Island, Bermuda | 32N | 64W |
| Thor amboinensis | MF378893 | Paget Island, Bermuda | 32N | 64W |
| Thor amboinensis | MF378894 | Paget Island, Bermuda | 32N | 64W |
| Thor amboinensis | MF378895 | Paget Island, Bermuda | 32N | 64W |
| Thor amboinensis | MF378896 | Paget Island, Bermuda | 32N | 64W |
| Thor amboinensis | MF378897 | Paget Island, Bermuda | 32N | 64W |
| Thor amboinensis | MF378898 | Paget Island, Bermuda | 32N | 64W |
| Thor amboinensis | MF378899 | Paget Island, Bermuda | 32N | 64W |
| Thor amboinensis | MF378900 | Paget Island, Bermuda | 32N | 64W |
| Thor amboinensis | MF378901 | Paget Island, Bermuda | 32N | 64W |
| Thor amboinensis | MF378902 | Paget Island, Bermuda | 32N | 64W |
| Thor amboinensis | MF378903 | Lagoonal Shelf 2 | 32N | 64W |
| Thor amboinensis | MF378904 | Lagoonal Shelf 2 | 32N | 64W |
| Thor amboinensis | MF378905 | Lagoonal Shelf 2 | 32N | 64W |
| Thor amboinensis | MF378906 | Lagoonal Shelf 2 | 32N | 64W |
| Thor amboinensis | MF378907 | Lagoonal Shelf 2 | 32N | 64W |
| Thor amboinensis | MF378908 | Lagoonal Shelf 5 | 32N | 64W |
| Thor amboinensis | MF378909 | Lagoonal Shelf 5 | 32N | 64W |
| Thor amboinensis | MF378910 | Lagoonal Shelf 5 | 32N | 64W |
| Thor amboinensis | MF378911 | Lagoonal Shelf 5 | 32N | 64W |
| Thor amboinensis | MF378912 | Lagoonal Shelf 5 | 32N | 64W |
| Thor amboinensis | MF378913 | Lagoonal Shelf 5 | 32N | 64W |
| Thor amboinensis | MF378914 | Lagoonal Shelf 5 | 32N | 64W |
| Thor amboinensis | MF378921 | Snake Bay, Curacao | 12N | 68W |
| Thor amboinensis | MF378922 | Snake Bay, Curacao | 12N | 68W |
| Thor amboinensis | MF378923 | Snake Bay, Curacao | 12N | 68W |
| Thor amboinensis | MF378924 | Snake Bay, Curacao | 12N | 68W |
| Thor amboinensis | MF378925 | Snake Bay, Curacao | 12N | 68W |
| Thor amboinensis | MF378926 | Snake Bay, Curacao | 12N | 68W |
| Thor amboinensis | MF378927 | Snake Bay, Curacao | 12N | 68W |
| Thor amboinensis | MF378928 | Snake Bay, Curacao | 12N | 68W |
| Thor amboinensis | MF378929 | Snake Bay, Curacao | 12N | 68W |
| Thor amboinensis | MF378930 | Snake Bay, Curacao | 12N | 68W |
| Thor amboinensis | MF378931 | Snake Bay, Curacao | 12N | 68W |
| Thor amboinensis | MF378932 | Snake Bay, Curacao | 12N | 68W |
| Thor amboinensis | MF378933 | Snake Bay, Curacao | 12N | 68W |
| Thor amboinensis | MF378934 | Snake Bay, Curacao | 12N | 68W |
| Thor amboinensis | MF378935 | Carmabi, Curacao | 12N | 68W |
| Thor amboinensis | MF378936 | Carmabi, Curacao | 12N | 68W |
| Thor amboinensis | MF378937 | Carmabi, Curacao | 12N | 68W |
| Thor amboinensis | MF378938 | Carmabi, Curacao | 12N | 68W |
| Thor amboinensis | MF378939 | Carmabi, Curacao | 12N | 68W |
| Thor amboinensis | MF378940 | Carmabi, Curacao | 12N | 68W |
| Thor amboinensis | MF378941 | Carmabi, Curacao | 12N | 68W |
| Thor amboinensis | MF378942 | Carmabi, Curacao | 12N | 68W |
| Thor amboinensis | MF378943 | Carmabi, Curacao | 12N | 68W |
| Thor amboinensis | MF378944 | Carmabi, Curacao | 12N | 68W |
| Thor amboinensis | MF378945 | Carmabi, Curacao | 12N | 68W |
| Thor amboinensis | MF378946 | Carmabi, Curacao | 12N | 68W |
| Thor amboinensis | MF378947 | Carmabi, Curacao | 12N | 68W |
| Thor amboinensis | MF378948 | Carmabi, Curacao | 12N | 68W |
| Thor amboinensis | MF378949 | Carmabi, Curacao | 12N | 68W |
| Thor amboinensis | MF378950 | Carmabi, Curacao | 12N | 68W |
| Thor amboinensis | MF378951 | Carmabi, Curacao | 12N | 68W |
| Thor amboinensis | MF378952 | Carmabi, Curacao | 12N | 68W |
| Thor amboinensis | MF378953 | Carmabi, Curacao | 12N | 68W |
| Thor amboinensis | MF378954 | Carmabi, Curacao | 12N | 68W |
| Thor amboinensis | MF378955 | Carmabi, Curacao | 12N | 68W |
| Thor amboinensis | MF378956 | Carmabi, Curacao | 12N | 68W |
| Thor amboinensis | MF378957 | Carmabi, Curacao | 12N | 68W |
| Thor amboinensis | MF378958 | Carmabi, Curacao | 12N | 68W |
| Thor amboinensis | MF378959 | Carmabi, Curacao | 12N | 68W |
| Thor amboinensis | MF378960 | Carmabi, Curacao | 12N | 68W |
| Thor amboinensis | MF378961 | Carmabi, Curacao | 12N | 68W |
| Thor amboinensis | MF378962 | Carmabi, Curacao | 12N | 68W |
| Thor amboinensis | MF378963 | Carmabi, Curacao | 12N | 68W |
| Thor amboinensis | KX926335 | Fort Lauderdale, Florida, USA | 26N | 80W |
| Thor amboinensis | KX926336 | Fort Lauderdale, Florida, USA | 26N | 80W |
| Thor amboinensis | KX926337 | Fort Lauderdale, Florida, USA | 26N | 80W |
| Thor amboinensis | KX926338 | Fort Lauderdale, Florida, USA | 26N | 80W |
| Thor amboinensis | KX926392 | Middle Keys, Florida, USA | 24N | 80W |
| Thor amboinensis | KX926393 | Middle Keys, Florida, USA | 24N | 80W |
| Thor amboinensis | KX926394 | Middle Keys, Florida, USA | 24N | 80W |
| Thor amboinensis | KX926395 | Middle Keys, Florida, USA | 24N | 80W |
| Thor amboinensis | KX926396 | Middle Keys, Florida, USA | 24N | 80W |
| Thor amboinensis | KX926397 | Middle Keys, Florida, USA | 24N | 80W |
| Thor amboinensis | KX926398 | Middle Keys, Florida, USA | 24N | 80W |
| Thor amboinensis | KX926399 | Middle Keys, Florida, USA | 24N | 80W |
| Thor amboinensis | KX926400 | Middle Keys, Florida, USA | 24N | 80W |
| Thor amboinensis | KX926401 | Middle Keys, Florida, USA | 24N | 80W |
| Thor amboinensis | KX926402 | Middle Keys, Florida, USA | 24N | 80W |
| Thor amboinensis | KX926403 | Middle Keys, Florida, USA | 24N | 80W |
| Thor amboinensis | KX926404 | Middle Keys, Florida, USA | 24N | 80W |
| Thor amboinensis | KX926405 | Middle Keys, Florida, USA | 24N | 80W |
| Thor amboinensis | KX926406 | Middle Keys, Florida, USA | 24N | 80W |
| Thor amboinensis | KX926407 | Middle Keys, Florida, USA | 24N | 80W |
| Thor amboinensis | KX926408 | Middle Keys, Florida, USA | 24N | 80W |
| Thor amboinensis | KX926409 | Middle Keys, Florida, USA | 24N | 80W |
| Thor amboinensis | KX926410 | Middle Keys, Florida, USA | 24N | 80W |
| Thor amboinensis | KX926411 | Middle Keys, Florida, USA | 24N | 80W |
| Thor amboinensis | KX926412 | Middle Keys, Florida, USA | 24N | 80W |
| Thor amboinensis | KX926413 | Middle Keys, Florida, USA | 24N | 80W |
| Thor amboinensis | KX926414 | Middle Keys, Florida, USA | 24N | 80W |
| Thor amboinensis | KX926339 | Lower Keys, Florida, USA | 24N | 81W |
| Thor amboinensis | KX926340 | Lower Keys, Florida, USA | 24N | 81W |
| Thor amboinensis | KX926341 | Lower Keys, Florida, USA | 24N | 81W |
| Thor amboinensis | KX926342 | Lower Keys, Florida, USA | 24N | 81W |
| Thor amboinensis | KX926343 | Lower Keys, Florida, USA | 24N | 81W |
| Thor amboinensis | KX926344 | Lower Keys, Florida, USA | 24N | 81W |
| Thor amboinensis | KX926345 | Lower Keys, Florida, USA | 24N | 81W |
| Thor amboinensis | KX926346 | Lower Keys, Florida, USA | 24N | 81W |
| Thor amboinensis | KX926347 | Lower Keys, Florida, USA | 24N | 81W |
| Thor amboinensis | KX926348 | Lower Keys, Florida, USA | 24N | 81W |
| Thor amboinensis | KX926349 | Lower Keys, Florida, USA | 24N | 81W |
| Thor amboinensis | KX926350 | Lower Keys, Florida, USA | 24N | 81W |
| Thor amboinensis | KX926351 | Lower Keys, Florida, USA | 24N | 81W |
| Thor amboinensis | KX926352 | Lower Keys, Florida, USA | 24N | 81W |
| Thor amboinensis | KX926353 | Lower Keys, Florida, USA | 24N | 81W |
| Thor amboinensis | KX926354 | Lower Keys, Florida, USA | 24N | 81W |
| Thor amboinensis | KX926355 | Lower Keys, Florida, USA | 24N | 81W |
| Thor amboinensis | KX926356 | Lower Keys, Florida, USA | 24N | 81W |
| Thor amboinensis | KX926357 | Lower Keys, Florida, USA | 24N | 81W |
| Thor amboinensis | KX926358 | Lower Keys, Florida, USA | 24N | 81W |
| Thor amboinensis | KX926359 | Lower Keys, Florida, USA | 24N | 81W |
| Thor amboinensis | KX926360 | Lower Keys, Florida, USA | 24N | 81W |
| Thor amboinensis | KX926361 | Lower Keys, Florida, USA | 24N | 81W |
| Thor amboinensis | KX926362 | Lower Keys, Florida, USA | 24N | 81W |
| Thor amboinensis | KX926363 | Lower Keys, Florida, USA | 24N | 81W |
| Thor amboinensis | KX926364 | Lower Keys, Florida, USA | 24N | 81W |
| Thor amboinensis | KX926365 | Lower Keys, Florida, USA | 24N | 81W |
| Thor amboinensis | KX926366 | Lower Keys, Florida, USA | 24N | 81W |
| Thor amboinensis | KX926367 | Lower Keys, Florida, USA | 24N | 81W |
| Thor amboinensis | KX926368 | Lower Keys, Florida, USA | 24N | 81W |
| Thor amboinensis | KX926369 | Lower Keys, Florida, USA | 24N | 81W |
| Thor amboinensis | KX926370 | Lower Keys, Florida, USA | 24N | 81W |
| Thor amboinensis | KX926371 | Lower Keys, Florida, USA | 24N | 81W |
| Thor amboinensis | KX926372 | Lower Keys, Florida, USA | 24N | 81W |
| Thor amboinensis | KX926373 | Lower Keys, Florida, USA | 24N | 81W |
| Thor amboinensis | KX926374 | Lower Keys, Florida, USA | 24N | 81W |
| Thor amboinensis | KX926375 | Lower Keys, Florida, USA | 24N | 81W |
| Thor amboinensis | KX926376 | Lower Keys, Florida, USA | 24N | 81W |
| Thor amboinensis | KX926377 | Lower Keys, Florida, USA | 24N | 81W |
| Thor amboinensis | KX926378 | Lower Keys, Florida, USA | 24N | 81W |
| Thor amboinensis | KX926379 | Lower Keys, Florida, USA | 24N | 81W |
| Thor amboinensis | KX926380 | Lower Keys, Florida, USA | 24N | 81W |
| Thor amboinensis | KX926381 | Lower Keys, Florida, USA | 24N | 81W |
| Thor amboinensis | KX926382 | Lower Keys, Florida, USA | 24N | 81W |
| Thor amboinensis | KX926383 | Lower Keys, Florida, USA | 24N | 81W |
| Thor amboinensis | KX926384 | Lower Keys, Florida, USA | 24N | 81W |
| Thor amboinensis | KX926385 | Lower Keys, Florida, USA | 24N | 81W |
| Thor amboinensis | KX926386 | Lower Keys, Florida, USA | 24N | 81W |
| Thor amboinensis | KX926387 | Lower Keys, Florida, USA | 24N | 81W |
| Thor amboinensis | KX926388 | Lower Keys, Florida, USA | 24N | 81W |
| Thor amboinensis | KX926389 | Lower Keys, Florida, USA | 24N | 81W |
| Thor amboinensis | MF378964 | Utila, Honduras | 16N | 86W |
| Thor amboinensis | MF378965 | Utila, Honduras | 16N | 86W |
| Thor amboinensis | MF378966 | Utila, Honduras | 16N | 86W |
| Thor amboinensis | MF378967 | Utila, Honduras | 16N | 86W |
| Thor amboinensis | MF378968 | Utila, Honduras | 16N | 86W |
| Thor amboinensis | MF378969 | Utila, Honduras | 16N | 86W |
| Thor amboinensis | MF378970 | Utila, Honduras | 16N | 86W |
| Thor amboinensis | MF378971 | Utila, Honduras | 16N | 86W |
| Thor amboinensis | MF378972 | Utila, Honduras | 16N | 86W |
| Thor amboinensis | MF378973 | Utila, Honduras | 16N | 86W |
| Thor amboinensis | MF378974 | Utila, Honduras | 16N | 86W |
| Thor amboinensis | MF378975 | Utila, Honduras | 16N | 86W |
| Thor amboinensis | MF378976 | Utila, Honduras | 16N | 86W |
| Thor amboinensis | MF378977 | Utila, Honduras | 16N | 86W |
| Thor amboinensis | MF378978 | Utila, Honduras | 16N | 86W |
| Thor amboinensis | MF378979 | Utila, Honduras | 16N | 86W |
| Thor amboinensis | MF378980 | Utila, Honduras | 16N | 86W |
| Thor amboinensis | MF378981 | Utila, Honduras | 16N | 86W |
| Thor amboinensis | MF378982 | Utila, Honduras | 16N | 86W |
| Thor amboinensis | MF378983 | Utila, Honduras | 16N | 86W |
| Thor amboinensis | MF378984 | Utila, Honduras | 16N | 86W |
| Thor amboinensis | MF378985 | Utila, Honduras | 16N | 86W |
| Thor amboinensis | MF378986 | Utila, Honduras | 16N | 86W |
| Thor amboinensis | MF378987 | Cayos Cochinos, Honduras | 16N | 86W |
| Thor amboinensis | MF378988 | Cayos Cochinos, Honduras | 16N | 86W |
| Thor amboinensis | MF378989 | Cayos Cochinos, Honduras | 16N | 86W |
| Thor amboinensis | MF378991 | Mahahual, Mexico | 18N | 87W |
| Thor amboinensis | MF378992 | Mahahual, Mexico | 18N | 87W |
| Thor amboinensis | MF378993 | Mahahual, Mexico | 18N | 87W |
| Thor amboinensis | MF378994 | Mahahual, Mexico | 18N | 87W |
| Thor amboinensis | MF378995 | Mahahual, Mexico | 18N | 87W |
| Thor amboinensis | MF378996 | Mahahual, Mexico | 18N | 87W |
| Thor amboinensis | MF378997 | Mahahual, Mexico | 18N | 87W |
| Thor amboinensis | MF378998 | Mahahual, Mexico | 18N | 87W |
| Thor amboinensis | MF378999 | Mahahual, Mexico | 18N | 87W |
| Thor amboinensis | MF379000 | Mahahual, Mexico | 18N | 87W |
| Thor amboinensis | MF379001 | Mahahual, Mexico | 18N | 87W |
| Thor amboinensis | MF379002 | Mahahual, Mexico | 18N | 87W |
| Thor amboinensis | MF379003 | Mahahual, Mexico | 18N | 87W |
| Thor amboinensis | MF379004 | Mahahual, Mexico | 18N | 87W |
| Thor amboinensis | MF379005 | Mahahual, Mexico | 18N | 87W |
| Thor amboinensis | MF379006 | Mahahual, Mexico | 18N | 87W |
| Thor amboinensis | MF379007 | Mahahual, Mexico | 18N | 87W |
| Thor amboinensis | MF379008 | Mahahual, Mexico | 18N | 87W |
| Thor amboinensis | MF379009 | Mahahual, Mexico | 18N | 87W |
| Thor amboinensis | MF379010 | Mahahual, Mexico | 18N | 87W |
| Thor amboinensis | MF379011 | Mahahual, Mexico | 18N | 87W |
| Thor amboinensis | MF379012 | Mahahual, Mexico | 18N | 87W |
| Thor amboinensis | MF379013 | Mahahual, Mexico | 18N | 87W |
| Thor amboinensis | MF379014 | Mahahual, Mexico | 18N | 87W |
| Thor amboinensis | MF379015 | Mahahual, Mexico | 18N | 87W |
| Thor amboinensis | MF379016 | Mahahual, Mexico | 18N | 87W |
| Thor amboinensis | MF379017 | Mahahual, Mexico | 18N | 87W |
| Thor amboinensis | MF379018 | Mahahual, Mexico | 18N | 87W |
| Thor amboinensis | MF379019 | Villa Nueva, Mexico | 18N | 87W |
| Thor amboinensis | MF379020 | Villa Nueva, Mexico | 18N | 87W |
| Thor amboinensis | MF379022 | Swan Cay, Bocas del Toro, Panama | 9N | 82W |
| Thor amboinensis | MF379023 | Swan Cay, Bocas del Toro, Panama | 9N | 82W |
| Thor amboinensis | MF379024 | Swan Cay, Bocas del Toro, Panama | 9N | 82W |
| Thor amboinensis | MF379025 | Swan Cay, Bocas del Toro, Panama | 9N | 82W |
| Thor amboinensis | MF379026 | Swan Cay, Bocas del Toro, Panama | 9N | 82W |
| Thor amboinensis | MF379027 | Swan Cay, Bocas del Toro, Panama | 9N | 82W |
| Thor amboinensis | MF379028 | Swan Cay, Bocas del Toro, Panama | 9N | 82W |
| Thor amboinensis | MF379029 | Swan Cay, Bocas del Toro, Panama | 9N | 82W |
| Thor amboinensis | MF379030 | Swan Cay, Bocas del Toro, Panama | 9N | 82W |
| Thor amboinensis | MF379031 | Swan Cay, Bocas del Toro, Panama | 9N | 82W |
| Thor amboinensis | MF379032 | Swan Cay, Bocas del Toro, Panama | 9N | 82W |
| Thor amboinensis | MF379033 | Swan Cay, Bocas del Toro, Panama | 9N | 82W |
| Thor amboinensis | MF379034 | Swan Cay, Bocas del Toro, Panama | 9N | 82W |
| Thor amboinensis | MF379035 | Swan Cay, Bocas del Toro, Panama | 9N | 82W |
| Thor amboinensis | MF379036 | Swan Cay, Bocas del Toro, Panama | 9N | 82W |
| Thor amboinensis | MF379037 | Swan Cay, Bocas del Toro, Panama | 9N | 82W |
| Thor amboinensis | MF379038 | Swan Cay, Bocas del Toro, Panama | 9N | 82W |
| Thor amboinensis | MF379039 | Swan Cay, Bocas del Toro, Panama | 9N | 82W |
| Thor amboinensis | MF379040 | Swan Cay, Bocas del Toro, Panama | 9N | 82W |
| Thor amboinensis | MF379041 | Swan Cay, Bocas del Toro, Panama | 9N | 82W |
| Thor amboinensis | MF379042 | Swan Cay, Bocas del Toro, Panama | 9N | 82W |
| Thor amboinensis | MF379043 | Swan Cay, Bocas del Toro, Panama | 9N | 82W |
| Thor amboinensis | MF379044 | Swan Cay, Bocas del Toro, Panama | 9N | 82W |
| Thor amboinensis | MF379045 | Swan Cay, Bocas del Toro, Panama | 9N | 82W |
| Thor amboinensis | MF379046 | Swan Cay, Bocas del Toro, Panama | 9N | 82W |
| Thor amboinensis | MF379047 | Swan Cay, Bocas del Toro, Panama | 9N | 82W |
| Thor amboinensis | MF379048 | Swan Cay, Bocas del Toro, Panama | 9N | 82W |
| Thor amboinensis | MF379049 | Swan Cay, Bocas del Toro, Panama | 9N | 82W |
| Thor amboinensis | MF379050 | Swan Cay, Bocas del Toro, Panama | 9N | 82W |
| Thor amboinensis | MF379051 | Bocas del Drago, Bocas del Toro, Panama | 9N | 82W |
| Thor amboinensis | MF379052 | Bocas del Drago, Bocas del Toro, Panama | 9N | 82W |
| Thor amboinensis | MF379053 | Bocas del Drago, Bocas del Toro, Panama | 9N | 82W |
| Clibanarius symmetricus | JN671540 | Bragança, Pará, Brazil | 0 | 46W |
| Clibanarius symmetricus | JN671541 | Bragança, Pará, Brazil | 0 | 46W |
| Clibanarius symmetricus | JN671542 | Bragança, Pará, Brazil | 0 | 46W |
| Clibanarius symmetricus | JN671543 | Bragança, Pará, Brazil | 0 | 46W |
| Clibanarius symmetricus | JN671544 | Bragança, Pará, Brazil | 0 | 46W |
| Clibanarius symmetricus | JN671550 | Ilhéus, Bahia, Brazil | 14S | 39W |
| Clibanarius symmetricus | JN671545 | Ilhéus, Bahia, Brazil | 14S | 39W |
| Clibanarius symmetricus | JN671546 | Ilhéus, Bahia, Brazil | 14S | 39W |
| Clibanarius symmetricus | JN671548 | Paraty, Rio de Janeiro, Brazil | 23S | 44W |
| Clibanarius symmetricus | JN671547 | Ubatuba, São Paulo, Brazil | 23S | 44W |
| Clibanarius symmetricus | JN671549 | São Sebastiao, São Paulo, Brazil | 23S | 44W |
| Clibanarius symmetricus | JN671551 | Ilha Comprida, São Paulo, Brazil | 24.7S | 47.5W |
| Clibanarius symmetricus | JN671552 | Ilha Comprida, São Paulo, Brazil | 24.7S | 47.5W |
| Clibanarius symmetricus | JN671553 | Ilha Comprida, São Paulo, Brazil | 24.7S | 47.5W |
| Clibanarius symmetricus | JN671554 | Ilha Comprida, São Paulo, Brazil | 24.7S | 47.5W |
| Clibanarius symmetricus | JN671555 | Ilha Comprida, São Paulo, Brazil | 24.7S | 47.5W |
| Clibanarius symmetricus | JN671556 | Guaratuba, Paraná, Brazil | 25S | 48W |
| Clibanarius symmetricus | JN671557 | Guaratuba, Paraná, Brazil | 25S | 48W |
| Clibanarius symmetricus | JX238505 | Guaratuba, Paraná, Brazil | 25S | 48W |
| Clibanarius symmetricus | JN671558 | Florianópolis, Santa Catarina, Brazil | 27S | 48W |
| Clibanarius symmetricus | JN671559 | Florianópolis, Santa Catarina, Brazil | 27S | 48W |
| Clibanarius symmetricus | JN671560 | Florianópolis, Santa Catarina, Brazil | 27S | 48W |

Table S2. Results of the comparison among null, first, second, third, and fourth-order polynomial regressions using latitude as the predictor variable and nucleotide or haplotype diversity as the response variable for each species. Values in bold represent the selected model.

|  |  |  |  |  |  |  |  |
| --- | --- | --- | --- | --- | --- | --- | --- |
| **Acanthonyx petiverii** | | | | | | | |
| *Nucleotide Diversity* | | | | *Haplotype Diversity* | | | |
| Model | df | AIC | Adjusted-R2 | Model | df | AIC | Adjusted-R2 |
| **Null** | **2** | **-53.68986** |  | **Null** | **2** | **-0.9314629** |  |
| Linear | 3 | -52.30402 |  | Linear | 3 | -0.3412298 |  |
| Second order | 4 | -50.63784 |  | Second order | 4 | 0.5939614 |  |
| Third order | 5 | -50.51363 |  | Third order | 5 | 2.5515015 |  |
| Fourth order | 6 | Inf |  | Fourth order | 6 | Inf |  |
|  |  |  |  |  |  |  |  |
| **Aratus pisonii** | | | | | | | |
| *Nucleotide Diversity* | | | | *Haplotype Diversity* | | | |
| Model | df | AIC | Adjusted-R2 | Model | df | AIC | Adjusted-R2 |
| Null | 2 | -101.9799 |  | Null | 2 | 12.58708 |  |
| Linear | 3 | -101.2858 |  | Linear | 3 | 10.857376 |  |
| Second order | 4 | -104.8355 |  | Second order | 4 | 9.586129 |  |
| Third order | 5 | -103.9035 |  | Third order | 5 | 7.702444 |  |
| **Fourth order** | **6** | **-128.4628** | **0.94** | **Fourth order** | **6** | **-12.227023** | **0.93** |
|  |  |  |  |  |  |  |  |
| **Arenaeus cribrarius** | | | | | | | |
| *Nucleotide Diversity* | | | | *Haplotype Diversity* | | | |
| Model | df | AIC | Adjusted-R2 | Model | df | AIC | Adjusted-R2 |
| Null | 2 | -77.18419 |  | Null | 2 | -1.3328638 |  |
| Linear | 3 | -81.29491 |  | Linear | 3 | 0.2777807 |  |
| Second order | 4 | -85.96737 |  | Second order | 4 | 1.057912 |  |
| Third order | 5 | -85.96437 |  | Third order | 5 | -0.3769194 |  |
| **Fourth order** | **6** | **-94.81901** | **0.9** | **Fourth order** | **6** | **-5.6991356** | **0.5** |
|  |  |  |  |  |  |  |  |
| **Armases angustipes** | | | | | | | |
| *Nucleotide Diversity* | | | | *Haplotype Diversity* | | | |
| Model | df | AIC | Adjusted-R2 | Model | df | AIC | Adjusted-R2 |
| Null | 2 | -57.57807 |  | Null | 2 | -6.555272 |  |
| Linear | 3 | -56.95247 |  | **Linear** | **3** | **-10.502949** | **0.53** |
| Second order | 4 | -56.90803 |  | Second order | 4 | -9.305782 |  |
| Third order | 5 | -55.66836 |  | Third order | 5 | -7.436039 |  |
| **Fourth order** | **6** | **-95.6168** | **0.99** | Fourth order | 6 | -5.444697 |  |
|  |  |  |  |  |  |  |  |
| **Callinectes danae** | | | | | | | |
| *Nucleotide Diversity* | | | | *Haplotype Diversity* | | | |
| Model | df | AIC | Adjusted-R2 | Model | df | AIC | Adjusted-R2 |
| **Null** | **2** | **-98.22367** |  | **Null** | **2** | **7.513208** |  |
| Linear | 3 | -96.55475 |  | Linear | 3 | 9.306491 |  |
| Second order | 4 | -95.55912 |  | Second order | 4 | 10.110928 |  |
| Third order | 5 | -94.70784 |  | Third order | 5 | 10.963618 |  |
| Fourth order | 6 | -92.73205 |  | Fourth order | 6 | 11.992194 |  |
|  |  |  |  |  |  |  |  |
| **Callinectes ornatus** | | | | | | | |
| *Nucleotide Diversity* | | | | *Haplotype Diversity* | | | |
| Model | df | AIC | Adjusted-R2 | Model | df | AIC | Adjusted-R2 |
| Null | 2 | -69.53557 |  | Null | 2 | 7.2237573 |  |
| **Linear** | **3** | **-72.13965** | **0.37** | Linear | 3 | 4.8655099 |  |
| Second order | 4 | -72.85096 |  | Second order | 4 | 1.8768227 |  |
| Third order | 5 | -71.16149 |  | **Third order** | **5** | **-2.045832** | **0.77** |
| Fourth order | 6 | -69.79677 |  | Fourth order | 6 | -0.8220308 |  |
|  |  |  |  |  |  |  |  |
| **Eriphia gonagra** | | | | | | | |
| *Nucleotide Diversity* | | | | *Haplotype Diversity* | | | |
| Model | df | AIC | Adjusted-R2 | Model | df | AIC | Adjusted-R2 |
| Null | 2 | -52.09712 |  | Null | 2 | -11.83609 |  |
| Linear | 3 | -59.96521 |  | Linear | 3 | -12.07513 |  |
| **Second order** | **4** | **-66.76465** | **0.92** | Second order | 4 | -10.87535 |  |
| Third order | 5 | -64.82054 |  | Third order | 5 | -23.62041 |  |
| Fourth order | 6 | -62.93756 |  | **Fourth order** | **6** | **-27.73707** | **0.9** |
|  |  |  |  |  |  |  |  |
| **Goniopsis cruentata** | | | | | | | |
| *Nucleotide Diversity* | | | | *Haplotype Diversity* | | | |
| Model | df | AIC | Adjusted-R2 | Model | df | AIC | Adjusted-R2 |
| **Null** | **2** | **-122.8533** |  | **Null** | **2** | **-21.8813** |  |
| Linear | 3 | -123.6374 |  | Linear | 3 | -19.97229 |  |
| Second order | 4 | -122.1046 |  | Second order | 4 | -17.97312 |  |
| Third order | 5 | -122.327 |  | Third order | 5 | -17.66005 |  |
| Fourth order | 6 | -120.4206 |  | Fourth order | 6 | -15.66725 |  |
|  |  |  |  |  |  |  |  |
| **Leptuca leptodactyla** | | | | | | | |
| *Nucleotide Diversity* | | | | *Haplotype Diversity* | | | |
| Model | df | AIC | Adjusted-R2 | Model | df | AIC | Adjusted-R2 |
| **Null** | **2** | **-64.26743** |  | Null | 2 | 10.122621 |  |
| Linear | 3 | -63.5062 |  | Linear | 3 | 4.450461 |  |
| Second order | 4 | -63.39938 |  | Second order | 4 | 6.358959 |  |
| Third order | 5 | -63.40617 |  | Third order | 5 | 6.542061 |  |
| Fourth order | 6 | -61.76649 |  | **Fourth order** | **6** | **-1.675219** | **0.8** |
|  |  |  |  |  |  |  |  |
| **Leptuca thayeri** | | | | | | | |
| *Nucleotide Diversity* | | | | *Haplotype Diversity* | | | |
| Model | df | AIC | Adjusted-R2 | Model | df | AIC | Adjusted-R2 |
| **Null** | **2** | **-138.0471** |  | Null | 2 | -10.212098 |  |
| Linear | 3 | -136.8894 |  | Linear | 3 | -8.917338 |  |
| Second order | 4 | -135.3706 |  | Second order | 4 | -7.680812 |  |
| Third order | 5 | -133.4015 |  | Third order | 5 | -9.627194 |  |
| Fourth order | 6 | -134.4861 |  | **Fourth order** | **6** | **-16.371971** | **0.49** |
|  |  |  |  |  |  |  |  |
| **Sesarma rectum** | | | | | | | |
| *Nucleotide Diversity* | | | | *Haplotype Diversity* | | | |
| Model | df | AIC | Adjusted-R2 | Model | df | AIC | Adjusted-R2 |
| **Null** | **2** | **-78.17578** |  | **Null** | **2** | **12.08951** |  |
| Linear | 3 | -77.18203 |  | Linear | 3 | 13.38492 |  |
| Second order | 4 | -75.49098 |  | Second order | 4 | 15.13348 |  |
| Third order | 5 | -74.02897 |  | Third order | 5 | 17.07046 |  |
| Fourth order | 6 | -72.39785 |  | Fourth order | 6 | 17.15116 |  |
|  |  |  |  |  |  |  |  |
| Ucides cordatus | | | | | | | |
| *Nucleotide Diversity* | | | | *Haplotype Diversity* | | | |
| Model | df | AIC | Adjusted-R2 | Model | df | AIC | Adjusted-R2 |
| **Null** | **2** | **-105.55223** |  | **Null** | **2** | **-27.61555** |  |
| Linear | 3 | -103.62317 |  | Linear | 3 | -25.64462 |  |
| Second order | 4 | -102.51066 |  | Second order | 4 | -24.7534 |  |
| Third order | 5 | -100.57684 |  | Third order | 5 | -23.66716 |  |
| Fourth order | 6 | -99.71914 |  | Fourth order | 6 | -23.20692 |  |
|  |  |  |  |  |  |  |  |
| **Clibanarius antillensis** | | | | | | | |
| *Nucleotide Diversity* | | | | *Haplotype Diversity* | | | |
| Model | df | AIC | Adjusted-R2 | Model | df | AIC | Adjusted-R2 |
| **Null** | **2** | **-82.74027** |  | Null | 2 | Inf |  |
| Linear | 3 | -81.72046 |  | Linear | 3 | Inf |  |
| Second order | 4 | -80.11734 |  | Second order | 4 | Inf |  |
| Third order | 5 | -78.11805 |  | Third order | 5 | Inf |  |
| Fourth order | 6 | -76.49258 |  | Fourth order | 6 | Inf |  |
|  |  |  |  |  |  |  |  |
| **Clibanarius symmetricus** | | | | | | | |
| *Nucleotide Diversity* | | | | *Haplotype Diversity* | | | |
| Model | df | AIC | Adjusted-R2 | Model | df | AIC | Adjusted-R2 |
| Null | 2 | -48.96026 |  | Null | 2 | 9.182776 |  |
| Linear | 3 | -47.24441 |  | Linear | 3 | 10.194137 |  |
| Second order | 4 | -45.29442 |  | Second order | 4 | 10.13298 |  |
| Third order | 5 | -49.21451 |  | Third order | 5 | 2.222791 |  |
| **Fourth order** | **6** | **-54.89519** | **0.5** | **Fourth order** | **6** | **-14.15062** | **0.97** |
|  |  |  |  |  |  |  |  |
| **Calcinus tibicen (N)** | | | | | | | |
| *Nucleotide Diversity* | | | | *Haplotype Diversity* | | | |
| Model | df | AIC | Adjusted-R2 | Model | df | AIC | Adjusted-R2 |
| **Null** | **2** | **-63.09881** |  | Null | 2 | -1.308238 |  |
| Linear | 3 | -62.71025 |  | Linear | 3 | -1.433818 |  |
| Second order | 4 | -63.16938 |  | Second order | 4 | -18.867829 |  |
| Third order | 5 | -61.17151 |  | Third order | 5 | -26.163701 |  |
| Fourth order | 6 | -61.14909 |  | **Fourth order** | **6** | **-35.225286** | **0.99** |
|  |  |  |  |  |  |  |  |
| **Calcinus tibicen (S)** | | | | | | | |
| *Nucleotide Diversity* | | | | *Haplotype Diversity* | | | |
| Model | df | AIC | Adjusted-R2 | Model | df | AIC | Adjusted-R2 |
| Null | 2 | -62.39245 |  | Null | 2 | -14.7204 |  |
| Linear | 3 | -60.66298 |  | Linear | 3 | -13.024383 |  |
| Second order | 4 | -60.17475 |  | Second order | 4 | -11.42831 |  |
| **Third order** | **5** | **-68.80273** | **0.68** | Third order | 5 | -9.648155 |  |
| Fourth order | 6 | -69.59413 |  | **Fourth order** | **6** | **-32.626805** | **0.93** |
|  |  |  |  |  |  |  |  |
| **Clibanarius tricolor** | | | | | | | |
| *Nucleotide Diversity* | | | | *Haplotype Diversity* | | | |
| Model | df | AIC | Adjusted-R2 | Model | df | AIC | Adjusted-R2 |
| **Null** | **2** | **-51.26902** |  | Null | 2 | -70.34671 |  |
| Linear | 3 | -50.31491 |  | Linear | 3 | -68.3961 |  |
| Second order | 4 | -49.01693 |  | Second order | 4 | -67.07248 |  |
| Third order | 5 | -48.44831 |  | Third order | 5 | -66.99232 |  |
| Fourth order | 6 | -46.77335 |  | **Fourth order** | **6** | **-86.39288** | **0.9** |
|  |  |  |  |  |  |  |  |
| **Hippolyte obliquimanus** | | | | | | | |
| *Nucleotide Diversity* | | | | *Haplotype Diversity* | | | |
| Model | df | AIC | Adjusted-R2 | Model | df | AIC | Adjusted-R2 |
| Null | 2 | -40.95618 |  | Null | 2 | -2.288661 |  |
| Linear | 3 | -43.71241 |  | Linear | 3 | -2.965997 |  |
| Second order | 4 | -44.45164 |  | **Second order** | **4** | **-7.093111** | **0.65** |
| **Third order** | **5** | **-48.53018** | **0.73** | Third order | 5 | -6.263086 |  |
| Fourth order | 6 | Inf |  | Fourth order | 6 | Inf |  |
|  |  |  |  |  |  |  |  |
| **Minuca mordax** | | | | | | | |
| *Nucleotide Diversity* | | | | *Haplotype Diversity* | | | |
| Model | df | AIC | Adjusted-R2 | Model | df | AIC | Adjusted-R2 |
| Null | 2 | -90.97666 |  | Null | 2 | -24.18076 |  |
| **Linear** | **3** | **-102.70915** | **0.71** | **Linear** | **3** | **-30.76188** | **0.52** |
| Second order | 4 | -100.79377 |  | Second order | 4 | -31.90712 |  |
| Third order | 5 | -100.06298 |  | Third order | 5 | -30.78231 |  |
| Fourth order | 6 | -100.79276 |  | Fourth order | 6 | -29.70367 |  |
|  |  |  |  |  |  |  |  |
| **Minuca rapax** | | | | | | | |
| *Nucleotide Diversity* | | | | *Haplotype Diversity* | | | |
| Model | df | AIC | Adjusted-R2 | Model | df | AIC | Adjusted-R2 |
| **Null** | **2** | **-106.6856** |  | Null | 2 | 9.966956 |  |
| Linear | 3 | -104.8035 |  | Linear | 3 | 8.055279 |  |
| Second order | 4 | -106.8789 |  | Second order | 4 | 4.648715 |  |
| Third order | 5 | -108.5929 |  | **Third order** | **5** | **-2.969765** | **0.74** |
| Fourth order | 6 | -107.7804 |  | Fourth order | 6 | -1.691523 |  |
|  |  |  |  |  |  |  |  |
| **Panulirus argus** | | | | | | | |
| *Nucleotide Diversity* | | | | *Haplotype Diversity* | | | |
| Model | df | AIC | Adjusted-R2 | Model | df | AIC | Adjusted-R2 |
| Null | 2 | -47.70957 |  | Null | 2 | -26.50811 |  |
| Linear | 3 | -46.86006 |  | Linear | 3 | -26.58798 |  |
| Second order | 4 | -48.83612 |  | Second order | 4 | -27.00545 |  |
| **Third order** | **5** | **-49.95218** | **0.36** | **Third order** | **5** | **-28.88305** | **0.38** |
| Fourth order | 6 | -50.34157 |  | Fourth order | 6 | -32.41508 |  |
|  |  |  |  |  |  |  |  |
| **Thor amboinensis** | | | | | | | |
| *Nucleotide Diversity* | | | | *Haplotype Diversity* | | | |
| Model | df | AIC | Adjusted-R2 | Model | df | AIC | Adjusted-R2 |
| **Null** | **2** | **-100.38423** |  | Null | 2 | -2.844201 |  |
| Linear | 3 | -100.57193 |  | Linear | 3 | -3.679666 |  |
| Second order | 4 | -98.57277 |  | Second order | 4 | -1.717896 |  |
| Third order | 5 | -101.93931 |  | **Third order** | **5** | **-5.085498** | **0.37** |
| Fourth order | 6 | -100.67524 |  | Fourth order | 6 | -5.874269 |  |
|  |  |  |  |  |  |  |  |
| Uca maracoani | | | | | | | |
| *Nucleotide Diversity* | | | | *Haplotype Diversity* | | | |
| Model | df | AIC | Adjusted-R2 | Model | df | AIC | Adjusted-R2 |
| Null | 2 | -100.20489 |  | Null | 2 | 9.984868 |  |
| Linear | 3 | -99.08201 |  | Linear | 3 | 11.468717 |  |
| Second order | 4 | -102.0634 |  | Second order | 4 | 2.889483 |  |
| Third order | 5 | -100.33169 |  | Third order | 5 | 2.424693 |  |
| **Fourth order** | **6** | **-105.39206** | **0.51** | **Fourth order** | **6** | **-5.583127** | **0.82** |

Figure S1. Environmental variables values in the Tropical Western Atlantic region used in this study.


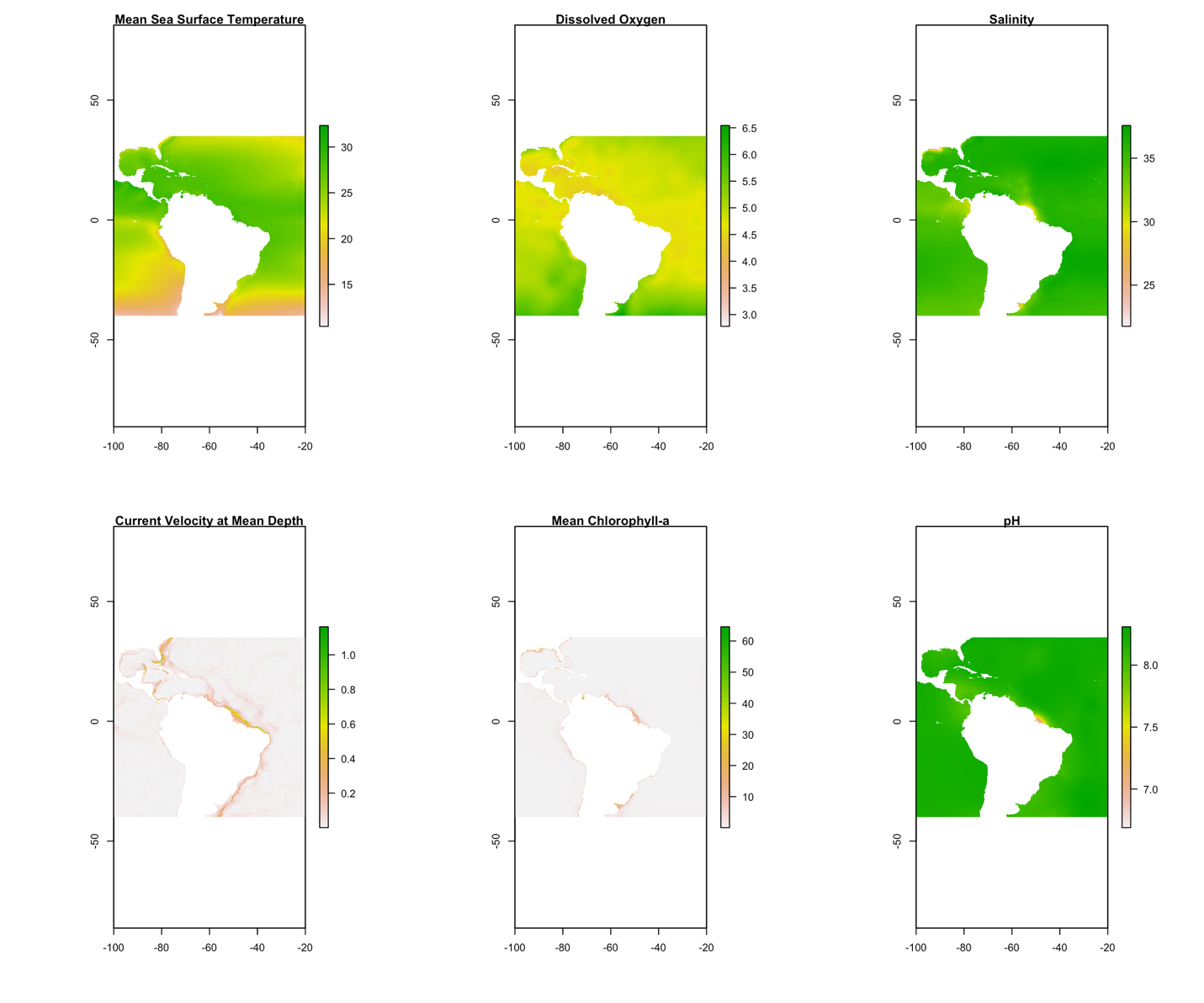


Figure S2. Intraspecific analyses testing the association between mitochondrial genetic diversity (nucleotide and haplotype diversity) vs. latitude
